# Climate change beliefs and their correlates in Latin America

**DOI:** 10.1038/s41467-023-42729-x

**Published:** 2023-11-09

**Authors:** Matias Spektor, Guilherme N. Fasolin, Juliana Camargo

**Affiliations:** 1 School of International Relations, Fundação Getulio Vargas, Avenida Paulista 542, São Paulo, 01310-000 Brazil; 2 Department of Political Science, Vanderbilt University, 230 Appleton PI, Nashville, TN 37203 USA

**Keywords:** Earth and environmental sciences, Environmental social sciences

## Abstract

The ability of climate skeptics to block climate action depends on prevailing beliefs among the public. Research in advanced democracies has shown skepticism about the existence, the causes, and the consequences of climate change to be associated with socio-demographic features and political ideology. Yet, little is known about climate-related beliefs elsewhere. We address this gap by mapping beliefs in climate change and their correlates in Latin America. We show skepticism over the existence and anthropogenic origins of climate change to be limited, but identify a high number of skeptics around the severity of its consequences. Furthermore, we show skepticism to be correlated with psychological rather than socio-political factors: individualistic worldviews in particular drive disbelief in the severe consequences of climate change, a worrying finding in contexts where social trust is low. These findings offer a starting point for better addressing the constraining effects of climate skepticism in the Global South.

## Abstract (web summary)

The authors map beliefs in climate change and their correlates in Latin America. The study shows skepticism over the existence and anthropogenic origins of climate change to be limited, but identifies a high number of skeptics around the severity of its consequences. Results also show individualistic worldviews to be the most powerful driver of climate change beliefs in the region.

## Introduction

Climate skepticism has driven scholars to investigate the range of factors influencing public perceptions of climate change^1,2^. Research suggests that political ideology and demography play an outsized role in shaping people’s beliefs about climate change, when compared to other social and psychological factors^3,4^. However, since the bulk of existing work focuses on advanced democracies like United States^5,6^, the United Kingdom^7,8^, Germany^9^, and Australia^10^, the question remains so to whether similar patterns occur in the developing world^11^.

In the case of Latin America, home to some of the most diverse and threatened biomes on the planet, the study of climate psychology has focused on climate change concern^12^ and climate risk perception^13^ rather than climate change beliefs, defined as propositional cognitions about the existence, causes, and consequences of climate change. A single study has inspected the factors associated with the belief in the anthropogenic origins of climate change, but not the critical question of beliefs in its existence and its consequences^14^. Other studies have explored the effect of country-level variables (e.g., prosperity and democracy) on climate change beliefs, but not the individual-level variables shaping such public perceptions^15,16^. These limitations are compounded by the fact that these studies draw on general surveys that lack dedicated climate sections in their questionnaires (e.g., The Gallup World Poll, Latinobarometer, and Latin American Public Opinion Project), leaving out a range of theoretically relevant individual-level variables.

This gap in knowledge is problematic because public beliefs in climate change condition climate action^17,18^, and Latin America is likely to be disproportionately affected by climate change^19^. Understanding the factors that are associated with public perceptions of climate change in developing countries^20–22^ is necessary if scholars and policy makers are to be able to mount effective strategies to push back against climate skepticism^23^ and tailor effective communication strategies to promote positive change in the context of the current climate crisis.

Here, we test the individual-level factors that shape people’s perceptions of the existence, the anthropogenic causes, and the severe consequences of climate change, three different but complementary mental constructs that scholars have shown to constitute climate change beliefs^24–26^. By fielding surveys to nationally diverse samples in Argentina, Brazil, Colombia, Chile, Ecuador, Peru, and Mexico (*N* = 5338), we are able to study a group of countries that account for more than 80% of regional carbon emissions in Latin America. We investigate key factors that previous literatures have shown to correlate with climate change beliefs. We first test the role for political ideology, a variable known to matter across advanced democracies^24,27,28^. We then turn to socio-demographic variables such as sex, age, education, religion, income, and race, correlates which previous studies have shown to be relevant in some settings but no others^3,29^. In addition, we test psychological variables such as individualism and egalitarianism worldviews, which previous literature has found to be impactful globally^27^ but not in Latin America^14^. We also assess the New Ecological Paradigm (NEP), a set of values that is strongly associated with climate change perceptions in countries as diverse as U.S., China, Germany and, Netherlands^30–32^. Finally, in order to ensure the comparability between our findings and other important works in the field, we assess the association between climate change beliefs and other psychological factors theorized as antecedents of climate change perceptions, namely; objective^33^ and subjective^34^ knowledge, trust in scientists^35^, scientific consensus^36^, and personal experience of extreme weather events^37,38^.

## Results

The overwhelming majority of people in Latin America perceive climate change to be happening (over 90% in all countries) (Supplementary Fig. 1) as a result of human activity (93% on average) (Supplementary Fig. 3). Yet, public opinion in the region is more divided about the severity of its consequences (an average of 65% of respondents believe climate change’s impact to be negative) (Supplementary Fig. 5). The remainder of this section summarizes our findings about the association between political and socio-demographic and psychological variables with beliefs about the existence, origins, and consequences of climate change. Supplementary Tables 1 and 4 present mean and standard deviations within each dependent and independent variable across the sample, while Supplementary Tables 2 and 6 display a summary of correlations among the measures in this study.

### Belief in the existence of climate change

Considering Latin America as a whole, psychological factors are the most associated with belief in the existence of climate change. The strongest correlate is perceived scientific consensus (respondents who hold this perception scored, on average, 0.49 points more on the climate change existence scale (0-8) than those who do not, holding all else constant (*p*-value < 0.01)), followed by NEP (*β* = 0.305, *p*-value < 0.01) and personal experience with extreme weather events (*β* = 0.232, *p*-value < 0.01) (Supplementary Table 12, column viii). These results are consistent across almost all countries at the 5% significance level (Supplementary Table 12, columns i to vii). However, these results should be taken cautiously because we find the reliability of the NEP scale to be low (*α* = 0.53 fails to meet the conventional 0.60 criterion for an acceptable Cronbach’s alpha).

**Fig. 1:**
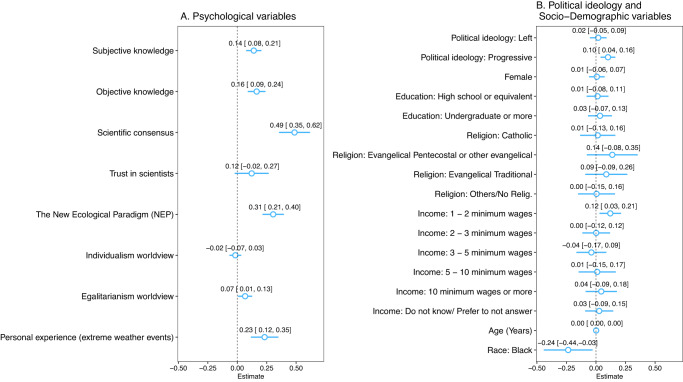
Correlates of Belief in the Existence of Climate Change in Latin America. Results from an ordinary least squares model regressing climate change existence on a set of independent variables (**A**) and socio-demographic characteristics (**B**). Respondents from all countries (*n* = 2887 observations) are included in the model. For each independent variable, point estimates of the coefficients and confidence intervals, in brackets, are reported. Coefficients are changes in the climate change existence scale (i.e., 0–8) given a unit increase in the covariates. The higher the scale, the greater the confidence that climate change is happening. The width of the confidence intervals for each coefficient is 95% with heteroskedasticity-robust standard errors. Reference baseline for Education is “Elementary (Primary) or less”, Religion is “Atheist” and Income is “0–1 minimum wages”.

Other psychological antecedents matter to a lesser extent (Fig. 1). For example, at the regional level belief in the existence of climate change has a positive correlation with people’s subjective and objective knowledge (*β* = 0.140, *p*-value < 0.01 and *β* = 0.165, *p*-value < 0.01, respectively) (Supplementary Table 12, column viii). Yet, at the country level subjective knowledge is only statistically significant in Ecuador, Mexico, and Peru (Supplementary Table 12, columns v, vi and vii) and objective knowledge is only statistically significant in Colombia, Ecuador and Peru (Supplementary Table 12, columns iv, v and vii). Furthermore, Latin Americans who trust scientists tend to believe climate change is happening more than those who do not, a positive association that we only observe for the overall sample at the 10% significance level (Supplementary Table 12, column viii). In addition, we find a positive association between egalitarian values and belief in the existence of climate change but it is small in magnitude and weakly consistent across countries (*β* = 0.067, *p*-value < 0.05, Supplementary Table 12, column viii). Finally, we find no statistically significant correlation between holding individualistic worldviews and belief in the reality of climate change. This result is consistent across countries.

Turning to socio-political factors, we find that political ideology is weakly associated with the belief in the existence of climate change in the overwhelming majority of countries (Fig. 1). We define political ideology alongside right-left and conservative-progressive scales which highlight contrasting individual beliefs over social and political change (see Methods). Taken as a whole, participants who self-identify as progressives tend to express less skepticism about the existence of climate change than their conservative co-nationals (*β* = 0.102, *p*-value < 0.05, Supplementary Table 12, column viii). At the country level, however, the relationship is statistically significant only for Brazil and Chile (Supplementary Table 12, columns ii and iii). Furthermore, results show that the left-right divide has no statistically significant correlation with the belief in the existence of climate change (*β* = 0.019, *p*-value > 0.1, Supplementary Table 12, column viii).

Socio-demographic variables too are weak correlates of the perception that climate change is happening (Fig. 1). For instance, when comparing respondents with no formal schooling or primary education to those with secondary (e.g., high school) and post-secondary education (e.g., undergraduate), there is not significant difference in their likelihood of believing in the existence of climate change. In addition, age (in years) fails to be a significant factor, as each additional year of age is not associated with a stronger belief in the reality of climate change. Moreover, our findings indicate that an increase in income, measured by each additional increment in minimum wages (ranging from 1 to 10 or more), is not correlated to a higher likelihood of believing in climate change. Similarly, the results demonstrate that female participants are not less likely to be skeptical about the existence of climate change than male co-nationals, dispelling any gender-based skepticism about this issue. These results hold regardless of our estimation approach (Supplementary Tables 25 and 26, column i). This said, race seems to be a potentially relevant factor: Fig. 1 reveals that black Latin Americans are more skeptical about the reality of climate change than their non-black co-nationals. We read these results cautiously, however, because the negative association is statistically significant in Argentina alone (Supplementary Table 12, column i).

### Belief in anthropogenic climate change

**Fig. 2:**
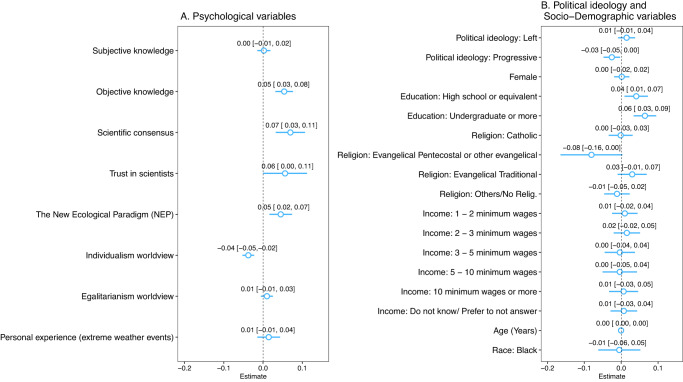
Correlates of Belief in the Anthropogenic Causes of Climate Change in Latin America. Results from an ordinary least squares (linear probability) model regressing the perception of climate change anthropogenic causes on a set of independent variables (**A**) and socio-demographic characteristics (**B**). Respondents from all countries (*n* = 2,883 observations) are included in the model. For each independent variable, point estimates of the coefficients and confidence intervals, in brackets, are reported. Coefficients multiplied by one hundred are percentage point changes in the probability of believing climate change is mainly caused by human activity given a unit increase in the covariates. The width of the confidence intervals for each coefficient is 95% with heteroskedasticity-robust standard errors. Reference baseline for Education is “Elementary (Primary) or less”, Religion is “Atheist” and Income is “0–1 minimum wages”.

Figure 2 displays the main correlates of the belief that human activity is the major cause of climate change. Among the psychological variables, individualistic worldviews stand out as the only negative, statistically significant correlate of skepticism about the anthropogenic origins of climate change. Considering Latin America as a whole, a one-unit increase in the individualistic values scale is associated, on average, with a reduction of 3.8 percentage points in the probability of believing in the anthropogenic nature of climate change (*p*-value < 0.01). With the exception of Ecuador and Mexico, the association is statistically significant across the board (Supplementary Table 13, columns v and vi).

Other psychological variables are positively associated with the belief in the anthropogenic nature of climate change: objective knowledge (*β* = 0.054, *p*-value < 0.01), perceived scientific consensus (*β* = 0.070, *p*-value < 0.01), and trust in scientists (*β* = 0.056, *p*-value < 0.05) (Supplementary Table 13, column viii). However, results are not consistent across all countries. Furthermore, endorsement of the NEP scale is positively correlated with belief in anthropogenic climate change in the entire sample (*β* = 0.045, *p*-value < 0.01, Supplementary Table 13, column viii), although we recall that the low internal validity of this scale prevents us from making reliable inferences. Overall, in terms of magnitude, these psychological variables have similar correlation coefficients with the perception of anthropogenic climate change in the model including all countries in the sample. The linear combination assessment of coefficients reveals they are not significantly different from each other at the 5% level and, thus, no psychological variable has a stronger association than the others (Supplementary Table 23).

We find political ideology to be weakly associated with skepticism about the anthropogenic origins of climate change (Fig. 2). The positive association between left-right and anthropogenic climate change is not statistically significant when we estimate our model for Latin America as a whole (*β* = 0.015, *p*-value > 0.1), (Supplementary Table 13, column viii). Analyzing each country separately, only Ecuador and Mexico have a positive statistically significant association between these variables (*β* = 0.072, *p*-value < 0.1 and *β* = 0.068, *p*-value < 0.05, respectively) (Supplementary Table 13, columns v and vii). We find that progressivism (as compared to conservatism) is negatively associated with skepticism in anthropogenic climate change (*β* = −0.025, *p*-value < 0.05) (Supplementary Table 13, column viii). The association is not statistically significant in 6 out of the 7 participating countries (Supplementary Table 13, columns i, ii, iv, v, vi and vii).

Finally, results show socio-demographic variables to be weakly correlated with the belief in anthropogenic climate change. For example, we find that sex, religion, income, age and race are not statistically correlated with the belief that climate change is caused by humans (Fig. 2). The exception—albeit one that is weakly uniform across countries—is our finding that more highly educated respondents are less skeptical about the causal role of human activity in causing climate change (Supplementary Table 13, column viii).

### Belief in the consequences of climate change

**Fig. 3:**
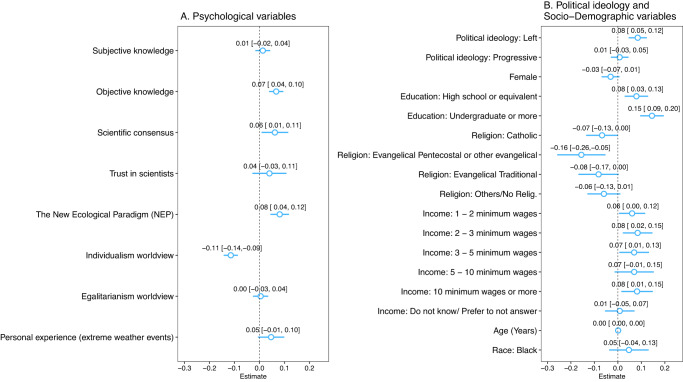
Correlates of Belief in the Consequences of Climate Change in Latin America. Results from an ordinary least squares (linear probability) model regressing the perception of climate change consequences on a set of independent variables (**A**) and socio-demographic characteristics (**B**). Respondents from all countries (*n* = 2887 observations) are included in the model. For each independent variable, point estimates of the coefficients and confidence intervals, in brackets, are reported. Coefficients multiplied by one hundred are percentage point changes in the probability of believing climate change consequences will be negative given a unit increase in the covariates. The width of the confidence intervals for each coefficient is 95% with heteroskedasticity-robust standard errors. Reference baseline for Education is “Elementary (Primary) or less”, Religion is “Atheist” and Income is “0–1 minimum wages”.

Among the set of psychological variables, individualistic worldviews has one of the strongest and statistically significant associations with beliefs about the consequences of climate change: people who score higher on individualistic values tend to see climate change’s impact as less negative than their co-nationals who score lower on that scale (Fig. 3). Specifically, considering the participating countries as a whole, a one-unit increase in the individualistic value scale is associated, on average, with an 11.5 percentage points (*p*-value < 0.01) reduction in the probability of an individual believing climate change will have negative impacts. This relationship is statistically significant, similar in size, and consistent across all countries in the sample (Supplementary Table 14, columns i through vii), suggesting that individualistic values shape public perceptions of climate impact across the region.

We find that objective knowledge also correlates with the perception of the consequences of climate change. Our analysis of the entire sample shows that the perception of more negative impacts of climate change is positively and statistically significantly associated with objective knowledge (*β* = 0.067, *p*-value < 0.01, Supplementary Table 14, column viii), a result observed in 4 out of the 7 countries in the sample (Supplementary Table 14, columns i, ii, iv and vii). While similar in magnitude, the relationships of other psychological variables are more heterogeneous across countries. For example, considering our sample as a whole, perceived scientific consensus has a positive and statistically significant association with belief in the severity of climate change (*β* = 0.062, *p*-value < 0.05, Supplementary Table 14, column viii), but, analyzing each country separately, it is statistically significant only for Chile (Supplementary Table 14, column iii). Furthermore, when assessing the entire sample, the association between the NEP scale and the perception of serious consequences is positive and statistically significant (*β* = 0.081, *p*-value < 0.01, Supplementary Table 14, column viii), but once again we take these results with caution due to our concerns about the scale’s reliability. Finally, we find that at the 5% significance level, subjective knowledge, trust in scientists, perceived personal experience with extreme weather events, and egalitarian values do not show a statistically significant association with the perception of climate change impacts (Fig. 3 and Supplementary Table 14, column viii).

Furthermore, results for the entire region show the association between political ideology and perceptions of climate change impact to be positive and statistically significant (Fig. 3). Specifically, we find that individuals on the left are more likely to perceive the impact of climate change as negative comparted to their co-nationals on the right (*β* = 0.085, *p*-value < 0.01) (Supplementary Table 14, column viii). Yet, at the country level, we find this relationship to be statistically significant only in 3 out of the 7 countries surveyed (Supplementary Table 14, columns ii, ii, and vi). In addition, we find no statistically significant association between conservatism and skepticism about the impact of climate change (*p*-value > 0.1) (Fig. 3).

Last but not least, the analysis of socio-demographic variables reveals two patterns (Fig. 3). First, education is the most consistent demographic correlate of perceived impacts of climate change. We find that individuals with secondary education (e.g., high school or equivalent), and particularly those with undergraduate education, are less doubtful about the negative impacts of climate change compared to individuals with only primary education or no formal education, suggesting that level of education may moderate the individual’s ability to concretize the effects of climate change (Supplementary Table 14, column viii). Second, in terms of statistical significance, demographic variables such as age, sex, race, income, and religion either have a moderate relationship with the perceived impacts of climate change or none at all. In a minority of countries, we find religious denominations such as Catholic, Evangelical Pentecostal, and Evangelical traditional to be associated with minimizing the severity of climate change (Supplementary Table 14, columns iii, iv and vii).

## Discussion

This study presents unique data on the individual-level factors that shape climate change perceptions in Latin America. Overall, mass publics across the region are highly concerned about climate change. We find only limited room for skepticism over the existence of climate change or its anthropogenic origins, but identify a high number of skeptics around the severity of its consequences. These results raise questions as to what factors might be behind these trends. We identify psychological variables—particularly individualistic values—as the most influential correlates of climate skepticism across the region, while we find political ideology and socio-demographic factors not to be as impactful. Taken together, these findings underscore the relevance of psychological factors in understanding climate change perceptions in Latin America and hold significant implications for the crafting of effective pro-climate communication strategies aimed at populations across this region of the Global South.

In the present study, individualistic values are the most powerful determinant of climate skepticism over the origins and consequences of climate change, but not its existence. These findings challenge previous literatures, which found no role for individualistic worldviews on the human origins of climate change in Latin America^14^. Furthermore, our results offer novel evidence that such worldviews are correlated with public perceptions of climate change consequences, an association of high political salience due to the known links between individual-level perceptions about the consequences of climate change and support for climate action^32^.

There are theoretical and empirical reasons for the outsized role of individualistic values as a driver of climate skepticism. Theoretically, it has been long established that people holding individualistic values downplay the human causes and the severity of climate change, since acknowledging them would demand changes in personal lifestyles that individualistic people typically resist^39^. Furthermore, research has shown that people holding more individualistic values are inclined to minimize climate risk scenarios because accepting them could lead to limitations on activities they tend to strongly support, such as commerce, industry, and free enterprise^40^. Since members of the public ranking high on individualistic values tend to be motivated reasoners^41^ (i.e., they are more likely to seek out information that reinforces their existing beliefs), an important question rising from this study that future research could explore is how best to persuade them to recognize the severity of climate change without triggering backlash^42,43^.

Empirically, the outsized role of individualistic values as determinant of climate skepticism mirrors wider social trends in Latin America. The region has seen a surge in independent self-construal, personal autonomy, self-expression, and achievement orientation, trends that may be contributing to the activation and intensification of individualism over reliance on state institutions^44^. Furthermore, Latin America has experienced rapid erosion in levels of social trust^45^, meaning the decline of dense community interactions around shared norms^46^. This erosion may in turn reinforce individualistic tendencies, as people become less likely to rely on collective action and institutions than on their own resources to resolve social problems. Future research could empirically test these ideas, in particular the relationship between social trust and individualistic values in shaping climate-related attitudes^47^.

Our finding that individualistic values do not correlate with the belief in the existence of climate change also requires explanation. Although the present study cannot adjudicate why this is the case, existing theoretical literatures provide useful foundations for a tentative interpretation. We know that beliefs in the causes and consequences of climate change can highlight the need for mitigation and adaptation policies more intensely than the belief in the mere existence of climate change, thereby raising the prospect of government intervention to cope with the effects of climate change^34^. In turn, this may trigger a negative reaction from highly individualistic people, who normally push back against big-government intrusion in their way of life^48^. These findings are consistent with new literature suggesting that climate skeptics are intuitively aware of the distinct dimensions of climate change beliefs, and have intentionally ceased to target belief in the existence of climate change (where scientific evidence is plentiful and increasingly hard to rebuke), questioning instead its human causes and the severity of its consequences^23,32^.

Two other psychological factors in this study also play a role in shaping climate change perceptions, although their influence proved to be limited and inconsistent across the sample: scientific consensus and objective knowledge. Scientific consensus is the most influential correlate of the belief in the existence of climate change, but it weakly correlates with beliefs in causes and consequences. One plausible explanation is that in the low-trust societies of Latin America the perception of consensus among the scientific community might in itself be reassuring, counterbalancing personal doubts about the credibility of climate science. It remains unclear, however, why this same logic does not apply to beliefs in causes and consequences. The lack of correlation between scientific consensus and beliefs on causes and consequences therefore casts a shadow of doubt on the notion established in existing literatures that scientific consensus should be seen as a ‘gateway belief’ that opens the door to other related beliefs, such as the anthropogenic causes and worrisome consequences of climate change^49,50^. Furthermore, it is worth noting that while scientific consensus in our study somewhat shapes beliefs in climate change, trust in scientists does not. The lack of association may indicate that people view consensus among scientists as more tangible evidence of the truth of climate change, compared to more abstract notions of trustworthiness.

Objective knowledge in this study is significant across all dimensions of climate change beliefs, albeit in a minority of sampled countries. This result confirms previous research showing positive relationships between climate beliefs and objective knowledge^34^. It also dovetails with existing literature noting that domain-specific knowledge is more useful than subjective knowledge measures in explaining climate change perceptions^51^. Although the effect of objective knowledge on beliefs varies across countries, investing in climate communication centered around it may be valuable to increase levels of belief in the existence, causes and consequences of climate change and, by implication, facilitating climate-friendly policies support and behavioral change^52^.

Other psychological variables in this study only had small or null impact in shaping climate change perceptions. For example, subjective knowledge, a factor that ranks consistently high in other studies on climate change perceptions, exhibited significant variation in our study on Latin America^27^. Moreover, mirroring empirical research conducted in the Global North^53^, we found limited and inconsistent associations between personal experience with extreme weather events and climate change beliefs. Overall, these heterogeneous results suggest that climate change beliefs in Latin America may be associated with a unique set of variables, in line with existing multi-countries studies on the topic^13,15^. A proper examination of climate change dynamics in the region requires more within-case studies and in-depth comparisons to understand why these factors vary cross-nationally. Future research should explore the contextual factors that contribute to differences in individual-level effects and their interaction with country-level conditions to explain these variations.

Political ideology and socio-demographic variables have limited influence on climate change perceptions in our sample. Unlike it is the case in the US^27,54^ and Western Europe^55^, in Latin America the divide between climate change skeptics and non-skeptics is not primarily driven by political ideology. Instead, the pattern here is more similar to findings from Central and Eastern Europe^56^, where political cleavages play a minor role in shaping climate change perceptions. One possible explanation is that people in less developed countries find it hard to see climate change through the prism of left-right or progressive-conservative divides, since the issue has been mostly restricted to intra-elite debates due to its relatively low salience compared to other pressing social problems^57^. These results bode well for the emergence of broad pro-climate coalitions in Latin America, since pro-climate action can garner public support from individuals across the political spectrum.

Our study also found weak associations between socio-demographic factors and perceptions of climate change. This result contradicts the theoretical literature suggesting that younger, more educated, higher income, and female individuals are less likely to be climate skeptics^29^. Empirically, the results also diverge from findings from the U.S^58^. and Western Europe (and to a lesser extent Eastern Europe)^3^, where demographics are important factors shaping climate change beliefs. The limited effects of certain demographic variables (e.g., education and sex) even differ from findings from other developing areas of the world, in particular Africa, where education and gender are important correlates of the belief in anthropogenic climate change^59^. Although future research needs to investigate what explains the weak socio-demographic associations in Latin America, these results highlight that demographic factors can carry different meanings in different contexts, with important implications for understanding beliefs about climate change. Finally, we are confident that our results are not driven by our convenience sample, as they align with studies using nationally representative samples in Latin America to assess other climate dimensions such as climate risk perception^13^ and climate change concern^12^.

Taken together, these results about the determinants of climate change beliefs can potentially help improve communication strategies aimed at promoting climate action. Although our findings only allow us to be speculative, existing theories in the scholarly literature can illuminate the path forward. First and foremost, since beliefs in Latin America in anthropogenic climate change and its consequences are influenced by individualistic values, messages are likely to be more effective when they are respectful of these values and align with these types of motivations^60^. For example, one strategy might be to advance climate solutions by highlighting the economic benefits (e.g., promoting green jobs) that arise from fighting climate change^61^. Emphasizing benefits may be particularly effective in garnering support for pro-environmental action not only because individualistic people are more likely to embrace solutions consistent with pro-market values, but also because these solutions help address broader development challenges in Latin America.

Second, the sources of pro-climate messages can play an important role in providing effective communication to the general public. Since individualistic people tend to be more influenced by their in-group, calls for climate action coming from in-group members should be prioritized at the expense of messages coming from sources who emphasize collective action and the common good^62,63^. This may include pro-climate messages coming from sources that are not typically seen as environment-friendly but who have in recent years nonetheless become invested in good governance in the climate sphere (e.g., bankers, business leaders, agribusiness). Future research should explore the effects of such voices on the public in general and on those members of the public who rank high in individualistic values and tend to exhibit a strong disdain or mistrust towards government interventions. Research to date in this field has been scant and primarily centered on advanced democracies alone.

Third, tailored communication techniques that leverage scientific consensus around the existence of climate change may nudge mass publics to engage in pro-climate action. While the existing literature emphasizes the positive downstream influence of scientific consensus on climate-friendly policies and behavior^64^, the challenge lies in effectively conveying this consensus to the public in societies characterized by low literacy rates and limited access to information on climate change. Across Latin America, the costs of accessing information are high and climate change is not politically salient. Taking these factors into account, communicating the existence of scientific consensus should be done through media that is easily accessible and seen as largely reliable, as it is the case of radio and television^65^. As recent research has shown, if communicators using these media were to be able to show how individuals can contribute to solving the climate challenge, their messages may help drive public climate action (e.g., voting, donating, protesting)^66^.

Last but not least, the current study is not without limitations. First, the cross-sectional nature of this analysis does not allow for the identification of causal relationships between variables, and there may be endogenous relationships that were not accounted for. Further research using experimental designs is necessary to establish causality and fully explore the implications of the findings presented here. Second, we acknowledge that our definition of individualistic values as personal attitudes towards the role of government in society is narrow, even if it is widely used in the field^4,67^. Many other aspects of this complex construct may influence climate change perceptions. Future studies should therefore explore broader conceptualizations of individualistic values, such as the extent to which individuals prioritize their personal interests over the interests of society as a whole. This approach may shed valuable light on the specific psychological mechanisms that underlie the relationship between individualism and climate skepticism, as well as inform the development of more targeted interventions to promote pro-climate behaviors. Third, drawing on the insight from the present study that individual perceptions of the role of government in society shape climate change beliefs, future work should explore the role of ideological liberalism, adding an extra layer to the study of political orientations in climate psychology. Fourth, our finding showing that personal experience with extreme weather is weakly associated with climate change beliefs may result from the fact that our question did not attribute weather events to climate change. As noted in Supplementary Information, we have some reason to believe individuals make this attribution intuitively in Latin America, but future work should follow recent research^68^ and explicitly test the consistency of these results.

In conclusion, this study has shed light on the correlates of climate change perceptions in Latin America. Now, armed with a clearer understanding of the variables associated with climate change perceptions in the region, pro-climate actors can develop more effective policies, strategies, and campaigns to confront climate skepticism head-on, a crucial step towards mitigating the impacts of climate change and promoting a more sustainable future. Ultimately, by building upon the findings of this study and continuing to investigate the correlates of climate change perceptions in diverse contexts, we can deepen our understanding of the social and psychological processes that shape beliefs not only in this particular region but across the Global South.

## Methods

### Participants

The study included nationally diverse participants from seven Latin American countries (Argentina, Brazil, Chile, Colombia, Ecuador, Peru, and Mexico), surveyed by the Netquest, an internet polling company, between October and November 2021. Together, these countries account for over 80% of greenhouse gas emissions (GHF) in Latin America. Netquest builds its online panels through an opt-in recruitment method, where respondents are randomly selected for survey invitation, using population quotas to produce nationally diverse samples. Netquest is certified with ISO 26362, an international high-quality standard for online panels, and complies with the European Society for Opinion and Market Research (ESOMAR). In general, this means that Netquest engages in a number of quality control steps, including the exclusion of speeders or preventing duplicate responders. Additional information on data quality and sampling is available in Netquest technical information reports^69^.

We chose Netquest because it provides comprehensive national panels in Latin America, which allow us to relatively approximate the national representativeness of the participating countries. In this study, respondents were recruited to match the demographic composition (e.g., quotas), particularly of sex, age, and education, laid out by the national census of each country surveyed. The final sample comprises 5400 participants, with all respondents above 18 years of age. Each country surveyed had roughly 830 respondents. The only exception was Ecuador, where just 421 respondents were interviewed due to panel coverage constraints. For demographic information broken for each of the countries, see Supplementary Table 8. Overall, the sample of each country is balanced with respect to sex and age, approximating closely to their respective official statistics (Supplementary Table 11). Moreover, one main concern of online samples is that their respondents tend to be more educated than the general population, a tendency that can be exacerbated in both lower and upper and middle-income countries^70^. In general terms, our sample is well-distributed in terms of education, with the overrepresentation of educated participants more pronounced in Ecuador and Peru (Supplementary Table 10). Nonetheless, we are cautious and all the models estimated in the study include sample weights to adjust for sample representativeness. Finally, our country samples are also broadly diverse in other important demographic indicators, including race, religion, and income level (Supplementary Table 8), which are known to correlate with important dimensions of climate change perceptions. More generally, online surveys in Latin America have been shown to by and large replicate results from nationally representative samples^71^, reinforcing the reliability of our sample.

### Materials

Questionnaires were first elaborated in Portuguese and then translated into Spanish by a native speaker and taking into account translation/back-translation procedures. Concerns about comprehension and translatability were addressed by testing and piloting of questionnaires in all seven countries contemplated in the survey.

### Dependent variable measures (climate change perceptions)

Building on previous research, we measure climate change perceptions along three theoretically distinct but complementary dimensions^24,32^. The distinction is relevant because scholars have shown individuals to hold inconsistent cognitions around the existence, the causes, and the consequences of climate change^32^, and evidence suggests each belief may be influenced by different antecedents^32,34^. To measure *belief in the existence of climate change*^17^^[,72^ participants were asked, “You may have heard the idea that the world’s climate is changing due to increases in average temperatures over the past 150 years. What’s your personal opinion on this? Do you think that the world’s climate is changing?” Respondents could then choose one of the following responses: “Yes (1), No (0), Don’t know.” Respondents who answered “yes” or “no” were then asked: “How sure are you that climate change is [not] happening?” (0 = not sure at all, 3 = extremely sure). Responses to these questions were recoded to create an eight-point certainty scale (0 = extremely sure global warming is not happening, 4 = don’t know, 8 = extremely sure global warming is happening). Belief in the *anthropogenic causes of climate change* was assessed with the question, “Assuming climate change is happening, do you think it is….” Respondents selected one of the four options: “Caused mostly by human activities, Caused by human activities and natural changes, Caused mostly by natural changes in the environment, Neither because global warming isn’t happening.” Following standard practice, respondents who answered “caused mostly by human activities” were coded as 1, while all others as 0^31,73^. Finally, we measure belief in the *consequences of climate change* with the standard question used in the literature^3^: “How good or bad do you think the impact of climate change will be on people across the world?” The question was measured using an 11-point scale, ranging from 0 (extremely bad) to 10 (extremely good).

### Independent variables

Knowledge about climate change was measured using one question that embraces participants’ subjective perception of their own expertise (subjective knowledge), and another that focuses on respondents’ knowledge about the human causes of climate change (objective knowledge*)*. We measured subjective knowledge by asking respondents the following question: “How much do you feel you know about climate change?” Participants rated their perceived amount of knowledge on a four-point scale (1 = nothing, 2 = a little, 3 = a moderate amount, 4 = a lot) but with a fifth option for people who “don’t know”. Objective knowledge was measured by asking participants, “Indicate whether you think each of the following is a major cause of climate change, or not a cause at all.” Six items were adopted from Guy et al.^34^, which include three true causes (“Pollution/emissions from business and industry”, “People driving their cars”, “Destruction of tropical forests”) and three false causes (“Use of aerosol spray cans”, “Use of chemicals to destroy insect pests”, and “Nuclear power generation”). Responses of items were coded as correct (1) or incorrect (0) and summed to create a total score ranging from 0 to 6.

Beliefs about science were measured through a question about scientific consensus around climate change (“consensus heuristic”), and another about trust in scientists (“source heuristic”). These two measures comprise the two main heuristics about science and climate change that have been implicated in the existing literature^27^. In the case of scientific consensus about climate change, participants were asked, “Which comes closest to your own view?” The response options were: “Most scientists think global warming is happening”, “A lot of disagreement among scientists about whether or not global warming is happening”, “Most scientists think global warming is not happening”, and “I do not know enough to say.” Respondents who answered “Most scientists think global warming is happening” were coded as 1, and all other answers as 0^73^. We measured trust in scientists by asking, “How much do you trust scientists as a source of information about climate change?” Participants rate their level of trust on a four-point scale (1 = strongly distrust; 4 = strongly trust)^35^.

To measure environmental values, we used a 4-item revised version from the New Ecological Paradigm scale based on previous research^74^. The items include “Humans are severely abusing the environment”, “The so-called ‘ecological crisis’ facing humankind has been greatly exaggerated”, “The earth is like a spaceship with very limited room and resources”, and “If things continue on their present course, we will soon experience a major ecological catastrophe.” Items were responded to on a four-point scale (1 = strongly disagree; 4 = strongly agree). There was also a fifth option for people who “don’t know”. The participants who chose the “don’t know” option were treated as missing values on this scale. The NEP scale was constructed by taking the mean scores across these four items. The original scale showed poor internal reliability across the whole sample (*α* = 0.37), which a closer analysis revealed to be driven by the answers to the second item, “The so-called ‘ecological crisis’ facing humankind has been greatly exaggerated”. Consequently, we conducted the analyses on the three positively worded items only, which makes the scale more reliable. This said, NEP continues to carry limitations, given its alpha (0.53) did not exceed the conventional 0.60 criteria. For this reason, we suggest caution in interpreting the results based on the NEP scale.

Values were measured using the individualism and egalitarianism cultural worldviews derived from cultural cognition theory. We follow the standard definition of worldviews as mental constructs pertaining to the role of the state in society rather than more expansive understandings of the relationship between individuals and society as a whole^4,67,75^. We operationalized our individualism scale on the basis of five items^76^, including the following: “If the government spent less time trying to fix everyone’s problems, we’d all be a lot better off”, “Our government tries to do too many things for too many people. We should just let people take care of themselves”, “The government interferes too much in our everyday lives”, “Government regulation of business usually does more harm than good”, and “People should be allowed to make as much money as they can, even if it means some make millions while others live in poverty.” All items were responded to a four point-scale (1 = strongly disagree; 4 = strongly agree). The individualism scale is constructed by taking the mean scores across these 5 items, which together showed acceptable internal reliability (*α* = 0.67). Note that the individualism scale has been reported in other studies in the Global South with similar internal consistency, underscoring the reliability of this measure in our study^67^.

Egalitarianism worldview is also a composite measure based on five items^76^. Items include: “The world would be a more peaceful place if its wealth were divided more equally among nations,” “In my ideal society, all basic needs (food, housing, health care, education) would be guaranteed by the government for everyone,” “I support government programs to get rid of poverty,” “Discrimination against minorities is still a very serious problem in our society.” All items were responded to a four point-scale (1 = strongly disagree; 4 = strongly agree). As in the case of the individualism scale, the scale of egalitarianism was created by taking the mean scores across these five items. The scale showed relatively strong internal reliability across the overall sample (*α* = 0.72).

Personal experience with extreme weather events was assessed by asking respondents to recall how often in the last five years they experienced extreme weather events (e.g., severe heat waves, droughts, freak storms, flooding etc.) while residing in their home country. Following previous studies, items were measured on a five-point scale (1 = never, 2 = once, 3 = twice, 4 = more than three, 5 = can’t remember) and responses were combined and dichotomized to form a dummy describing personal experience (0 = no experience, 1 = experience)^77^.

Political ideology was measured using two different questions. First, respondents were asked to rate how right or left they are on a ten-point scale (1 = left, 10 = right). Right-wing individuals generally advocate for limited government intervention in the economy, and traditional social values, while left-wing individuals typically support government intervention to address economic inequality, social justice issues, and expanded social welfare programs. Second, respondents were asked to choose the option that better characterizes their political values in a conservative-progressive dimension. The response options were rated on a five-point scale (1 = very progressive, 5 = very conservative). Conservatism can be defined as a political ideology that advocates for traditional values, limited government intervention, and the preservation of established institutions and social norms. By contrast, progressivism is a political ideology that promotes social and political change, often through government intervention, to address social injustices and improve the well-being of society. We opted to use the word “progressive” rather than “liberal” because “liberal” in Latin America can be associated with the orthodox economic policy preferences of the political right. “Progressive” (*progresista* in Spanish or *progressist* in Portuguese) provides a better characterization of what the existing literature labels as “liberal” in this context.

The demographic variables include sex (binary: male or female), age (in years), education level (ordinal: elementary (primary) or less; high school or equivalent; and undergraduate or more), religion (Evangelical Christian/Traditional; Evangelical Protestant; Evangelical Pentecostal; Evangelical Neo-Pentecostal; Other Evangelical denominations; Catholic; Kardecist/Spiritualist; Jewish; Agnostic; Atheist; Other Religion.), race (White; Black or *Pardo*); Indigenous; Other. In all countries (except for Brazil), we also include “Mestizo” as an option choice, given that it is a racial classification present in these countries), income-based on minimum wages (from 1 to 10 minimum wages or more).

### Statistical analysis

To evaluate what factors determine climate change beliefs in Latin America we estimated the following equation by ordinary least squares for each country and our whole sample:

1 $${y}_{i} \,=	\, {\beta }_{0}+{{{{{\rm{Psychological}}}}}}\,{{{{{{\rm{Variables}}}}}}}_{i}^{{\prime} }\gamma+{{{{{\rm{Political}}}}}}\,{{{{{\rm{Ideology}}}}}}\,{{{{{{\rm{Variables}}}}}}}_{i}^{{\prime} }\alpha \\ 	\,+\,{{{{{\rm{SocioDemographic}}}}}}\,{{{{{{\rm{Variables}}}}}}}_{i}^{{\prime} }\mu+{\varepsilon }_{i}$$

where ${y}_{i}$ is one of our three dependent variables (belief in the existence of climate change, belief in the anthropogenic causes of climate change, and belief in the consequences of climate change) for individual $i$; ${{{{{\rm{Psychological}}}}}}\; {{{{{\rm{Variables}}}}}}_{i}$ is a vector of eight psychological variables of individual $i$; ${{{{{\rm{Political}}}}}}\; {{{{{\rm{Ideology}}}}}}\; {{{{{\rm{Variables}}}}}}_{i}$ is a vector of two political ideology variables of individual $i$; and ${{{{{\rm{SocioDemographic}}}}}}\; {{{{{\rm{Variables}}}}}}_{i}$ is a vector of six population-related characteristics of individual $i$. Reference baseline for education is “elementary (primary) or less”, for religion is “atheist” and for income is “0–1 minimum wages”. Standard errors presented in the results are robust to heteroscedasticity and all estimations include sample weights.

For belief in the existence of climate change, the vector of parameters of interest, $\gamma,$ $\alpha$ and $\mu$, can be interpreted as changes in the climate change belief scale (0–8) given a unit increase in the covariates. For belief in the anthropogenic causes of climate change, each parameter of the vectors $\gamma,$ $\alpha$ and $\mu$ multiplied by one hundred can be interpreted as percentage point changes in the probability of believing climate change is mainly caused by human activity given a unit increase in the binary covariates. For belief in the consequences of climate change, $\gamma,$ $\alpha$ and $\mu$ multiplied by one hundred can be interpreted as percentage point changes in the probability of believing climate change impacts will be negative given a unit increase in the binary covariates.

To gauge the robustness of our ordinary least squares estimates we performed several tests by employing alternative methods to estimate the parameters of our main equation. First, we estimated three multilevel (hierarchical) models, one for each dependent variable, with a random intercept specified at the country-level (Supplementary Table 25). Second, we estimated three fixed-effects models, one for each dependent variable, by including country-level effects (Supplementary Table 26). Both of these approaches allow us to control for possible time-invariant unobserved heterogeneity between respondents of each country. Third, we used an ordinal logistic model to regress belief in the existence of climate change on the three vectors of correlates (Supplementary Table 27). Finally, we estimated two binary logistics models regressing the belief in the anthropogenic causes of climate change and belief in the consequences of climate change also on the three vectors (Supplementary Tables 28 and 29). Overall, the results are substantially similar irrespective of the alternative specifications of our main equation.

### Robustness checks

Besides employing alternative methods to estimate the parameters of our main equation (see Methods section), several complementary tests were performed to gauge the robustness of our findings. First, we provide evidence of the absence of multicollinearity. Supplementary Table 16 reveals that the variance inflation factor (VIF)—a measure of the amount of multicollinearity in regression analysis—is low for all our independent variables. All the VIPs are below 1.5, which relieves our concern about the potential correlation between the main variables.

Second, even after finding correlation among the independent variables to be low, we performed F-tests to mitigate concerns that the independent variables might not be jointly statistically significant. Supplementary Table 17 shows the results of the estimations of the joint significance test for all the models we ran in the study, considering all independent variables. We find that all independent variables are jointly significant in each of the four main OLS models estimated for the whole sample. We also ran the F-test of joint significance for different groups of independent variables separately (Supplementary Tables 18–20). For each different group of independent variables, we found that they are jointly significant in each of the four main OLS models estimated for the whole sample. These results indicate the models fit the data well and suggest that the independent variables included in the model are not irrelevant.

Finally, to attenuate concerns that some of our results might be statistically significant by chance, we ran a Benjamini-Hochberg test to adjust *p*-values for multiple hypothesis tests^78^. As shown in Supplementary Table 21, the overall results of the estimations using the adjusted *p*-values corroborate the results using standard *p*-values. All the statistical interpretations remain roughly the same, even after accounting for the multiple hypothesis tests. This suggests that our inferences about statistical significance are not an artifact of the number of predictors in our regression models.

### Ethics statement

This study was approved by the Ethical Review Committee of the Fundação Getulio Vargas (FGV) (Ethics no. 053/2021). All participants informed voluntary consent with an IRB-approved consent protocol before being allowed to proceed to the full questionnaire. The survey did not collect identifying information about respondents and/or use any type of deception. The panel provider modestly compensated the respondents with points they accumulate and then exchange for goods or cellphone minutes.

### Reporting summary

Further information on research design is available in the Nature Portfolio Reporting Summary linked to this article.

### Supplementary information

Supplementary Information

Reporting Summary

## Data Availability

The processed data used in this study are available at the Harvard Dataverse as Spektor, Matias; Fasolin, Guilherme; Camargo, Juliana, 2022, “Replication Data for: Climate Change Beliefs and their Correlates in Latin America”, 10.7910/DVN/F4KNNS, **Harvard Dataverse, V2**. The following software has been used to process the data: the R software (version 4.2.2) The integrated development environment (IDE) of choice was RStudio.

## Code availability

The code used in this study are available at the Harvard Dataverse as Spektor, Matias; Fasolin, Guilherme; Camargo, Juliana, 2022, “Replication Data for: Climate Change Beliefs and their Correlates in Latin America”, 10.7910/DVN/F4KNNS, **Harvard Dataverse, V2**.

## References

[1] Capstick, S. & Whitmarsh, L. Perceptions of climate change. In (eds Clayton, S. & Manning, C.) *Psychology and Climate Change: Human Perceptions, Impacts, and Responses*. 13–33 (Elsevier Academic Press, 2018).

[2] Clayton S (2015). Psychological research and global climate change. Nat. Clim. Chang..

[3] Poortinga W, Whitmarsh L, Steng L, Bohm G, Fisher S (2019). Climate change perceptions and their individual-level determinants: a cross-European analysis. Glob. Environ. Change.

[4] Hornsey MJ (2021). The role of worldviews in shaping how people appraise climate change. Curr. Opin. Behav. Sci..

[5] Howe P, Mildenberger M, Marlon J, Leiserowitz T (2015). Geographic variation in opinion on climate change at state and local scales in the USA. Nat. Clim. Chang..

[6] Leiserowitz, A. et al. *Climate change in the American mind, September 2021*. (Yale University and George Mason University. Yale Program on Climate Change Communication, New Haven, CT, 2021).

[7] Capstick SB, Pidgeon NF (2014). What is climate change scepticism? Examination of the concept using a mixed methods study of the UK public. Glob. Environ. Change.

[8] Whitmarsh L (2011). Scepticism and uncertainty about climate change: dimensions, determinants and change over time. Glob. Environ. Change.

[9] Engels A, Huther O, Schafer M, Held H (2013). Public climate-change scepticism, energy preferences and political participation. Glob. Environ. Change.

[10] Leviston, Z., Leitch, A., Greenhill, M., Leonard, R. & Walker, I. *Australians' Views of Climate Change* (Commonwealth Scientific and Industrial Research Organisation, 2011).

[11] Capstick S, Whitmarsh L, Poortinga W, Pidgeon N, Upham P (2015). International trends in public perceptions of climate change over the past quarter century. WIREs Clim. Change.

[12] Lewis GB, Palm R, Feng B (2019). Cross-national variation in determinants of climate change concern. Environ. Politics.

[13] Lee TM, Markowitz EM, Howe PD, Ko CY, Leiserowitz AA (2015). Predictors of public climate change awareness and risk perception around the world. Nat. Clim. Chang..

[14] Hornsey MJ, Harris EA, Fielding KS (2018). Relationships among conspiratorial beliefs, conservatism and climate scepticism across nations. Nat. Clim. Chang..

[15] Levi S (2021). Country-level conditions like prosperity, democracy, and regulatory culture predict individual climate change belief. Commun. Earth Environ..

[16] Azócar G (2021). Climate change perception, vulnerability, and readiness: inter-country variability and emerging patterns in Latin America. J. Environ. Stud. Sci..

[17] Goldberg M, Gustafson A, Ballew M, Rosenthal S, Leiserowitz A (2021). Identifying the most important predictors of support for climate policy in the United States. Behav. Public Policy.

[18] Spence A, Poortinga W, Butler C, Pidgeon NS (2011). Perceptions of climate change and willingness to save energy related to flood experience. Nat. Clim. Chang..

[19] IPCC. Summary for Policymakers (eds Pörtner, H.-O. et al.) in Climate Change 2022: Impacts, Adaptation, and Vulnerability. Contribution of Working Group II to the Sixth Assessment Report of the Intergovernmental Panel on Climate Change. (Cambridge University Press, Cambridge, UK and New York, USA, 2022).

[20] Hopkins D (2015). Country comparisons. Nat. Clim. Chang..

[21] Crona B, Wutich A, Brewis A, Garint M (2013). Perceptions of climate change: linking local and global perceptions through a cultural knowledge approach. Climatic Change.

[22] Editorial. (2022). Replication studies hold the key to generalization. Nat. Commun..

[23] Hornsey MJ, Fielding KL (2020). Understanding (and reducing) inaction on climate change. Soc. Issue Policy Rev..

[24] Poortinga W, Spence A, Whitmarsh L, Capstick S, Pidgeon NF (2011). Uncertain climate: an investigation into public scepticism about anthropogenic climate change. Glob. Environ. Change.

[25] Heath Y, Gifford R (2006). Free-market ideology and environmental degradation: the case of belief in global climate change. Environ. Behav..

[26] Bostrom A (2012). Causal thinking and support for climate change policies: International survey findings. Glob. Environ. Change.

[27] Hornsey MJ, Harris EA, Bain PG, Fielding KS (2016). Meta-analyses of the determinants and outcomes of belief in climate change. Nat. Clim. Chang..

[28] McCright A, Dunlap RE (2011). The politization of climate change and polarization in the American public’s views of global warming, 2001–2010. Sociological Q..

[29] Milfont TL, Milojev P, Greaves LM, Sibley CG (2015). Socio-structural and psychological foundations of climate change beliefs. N.Z. J. Psychol..

[30] Dunlap RE, Van Liere KD, Mertig AG, Jones RE (2000). Measuring endorsement of the new ecological paradigm: a revised NEP scale. J. Soc. Issues.

[31] Ziegler A (2017). Political orientation, environmental values, and climate change beliefs and attitudes: an empirical cross-country analysis. Energy Econ..

[32] Van Valkengoed AM, Steg L, Perlaviciute G (2021). Development and validation of a climate change perceptions scale. J. Environ. Psychol..

[33] Bord RJ, O’Connor RE, Fisher A (2000). In what sense does the public need to understand global climate change?. Public Underst. Sci..

[34] Guy S, Kashima Y, Walker I, O’Neill S (2014). Investigating the effects of knowledge and ideology on climate change beliefs. Eur. J. Soc. Psychol..

[35] Hmielowski JD, Feldman L, Myers TA, Leiserowitz A, Maibach E (2014). An attack on science? Media use, trust in scientists, and perceptions of global warming. Public Underst. Sci..

[36] McCright AM, Dunlap RE, Xiao C (2013). Perceived scientific agreement and support for government action on climate change in the USA. Climatic Change.

[37] Demski C, Capstick S, Pidgeon N, Sposato RG, Spence A (2017). Experience of extreme weather affects climate change mitigation and adaptation responses. Climatic Change.

[38] Myers TA, Maibach EW, Roser-Renouf C, Akerlof K, Leiserowitz AA (2013). The relationship between personal experience and belief in the reality of global warming. Nat. Clim. Chang..

[39] Kahan D (2010). Fixing the communication failure. Nature.

[40] Kahan, D. M. in *Handbook of Risk Theory* (eds Roeser, S. et al.) 725–759 (Springer, Dordrecht, 2012).

[41] Kahan, D. M. et al. *The Tragedy of the Risk-Perception Commons: Culture Conflict, Rationality Conflict, and Climate Change*. Temple University Legal Studies Research Paper No. 2011-26 (2011).

[42] Druckman JN, McGrath MC (2019). The evidence for motivated reasoning in climate change preference formation. Nat. Clim. Chang..

[43] Bayes R, Druckman JN, Goods A, Molden DC (2020). When and how different motives can drive motivated political reasoning. Political Psychol..

[44] Krys K, Vignoles VL, de Almeida I, Uchida Y (2022). Outside the “cultural binary”: understanding why latin american collectivist societies foster independent selves. Perspect. Psychol. Sci..

[45] Keefer, P. & Scartascini, C. Trust: the key to social cohesion and growth in Latin America and the Caribbean. *Inter American Development Bank*, (2022).

[46] Fukuyama, F. Trust: The Social Virtues and the Creation of Prosperity. (Free Press, 1995).

[47] Bodor A, Varjú V, Grunhut Z (2020). The effect of trust on the various dimensions of climate change attitudes. Sustainability.

[48] Kahan DM, Jenkins-Smith H, Braman D (2011). Cultural cognition of scientific consensus. J. Risk Res..

[49] Lewandowsky S, Gignac GE, Vaughan S (2013). The pivotal role of perceived scientific consensus in acceptance of science. Nat. Clim. Change.

[50] Cook J (2016). Consensus on consensus: a synthesis of consensus estimates on human-caused global warming. Environ. Res. Lett..

[51] Shi J (2016). Knowledge as a driver of public perceptions about climate change reassessed. Nat. Clim. Change.

[52] Ajzen I, Joyce N, Sheikh S, Cote NG (2011). Knowledge and the prediction of behavior: the role of information accuracy in the theory of planned behavior. Basic Appl. Soc. Psychol..

[53] Howe PD, Marlon JD, Mildenberger M, Shield BS (2019). How will climate change shape perceptions?. Environ. Res. Lett..

[54] Campbell E, Kotcher J, Maibach E, Rosenthal S, Leiserowitz A (2021). Predicting the importance of global warming as a voting issue among the registered voters in the United States. Curr. Res. Ecol. Soc. Psychol..

[55] McCright AM, Dunlap RE, Marquart-Pyatt ST (2016). Political ideology and views about climate change in the European Union. Environ. Politics.

[56] Chaisty P, Whiteeld S (2015). Attitudes towards the environment: Are post-communist societies (still) different?. Environ. Politics.

[57] Zechmeister E, Corral M (2013). Individual and contextual constraints on ideological labels in Latin America. Comp. Political Stud..

[58] McCright AM, Dunlap RE (2011). Cool dudes: the denial of climate change among conservative white males in the United States. Glob. Environ. Change.

[59] Simpson NP (2021). ClimateChange Literacy Africa. Nat. Clim. Chang..

[60] Hornsey MJ, Lewandowsky S (2022). A toolkit for understanding and addressing climate scepticism. Nat. Hum. Behav..

[61] Bain PG (2016). Co-benefits of addressing climate change can motivate action around the world. Nat. Clim. Change.

[62] Boon-Falleur M (2022). Leveraging social cognition to promote effective climate change mitigation. Nat. Clim. Chang..

[63] Goldberg MH, Gustafson A, Rosenthal SA, Leiserowitz A (2021). Shifting Republican views on climate change through targeted advertising. Nat. Clim. Change.

[64] Bayes R, Bolsen T, Druckman JN (2020). A research agenda for climate change communication and public opinion: the role of scientific consensus messaging and beyond. Environ. Commun..

[65] Goldberg MH (2019). The experience of consensus: video as an effective medium to communicate scientific agreement on climate change. Sci. Commun..

[66] Doherty K, Webler T (2016). Social norms and efficacy beliefs drive the Alarmed segment’s public-sphere climate actions. Nat. Clim. Change.

[67] Thaker J, Smith N, Leiserowitz A (2020). Global Warming Risk Perceptions in India. Risk Anal..

[68] Ogunbode CA, Demski C, Capstick SB, Gennaro R (2019). Attribution matters: revisiting the link between extreme weather experience and climate change mitigation responses. Glob. Environ. Change.

[69] *Netquest answers to ESOMAR 28 questions to help online research buyers* (Netquest, 2015).

[70] The World Bank. World Bank Country and Lend Groups. https://datahelpdesk.worldbank.org/knowledgebase/articles/906519-world-bank-country-and-lending-groups#:~:text=For%20the%20current%202024%20fiscal,those%20with%20a%20GNI%20per. (2023).

[71] Bush, S. S., & Clayton, A. Facing Gender: Gender and Climate Change Attitudes Worldwide. *Am. Political Sci. Rev*. First View, 1–18 (Forthcoming).

[72] Bergquist P, Mildernberg M, Stokes LC (2020). Combining climate, economic, and social policy builds up support for climate action in the US. Environ. Res. Lett..

[73] Howe P, Mildenberger M, Marlon JR, Leiserowitz A (2015). Geographic variation in opinions on climate change at state and local scales in the USA. Nat. Clim. Chang..

[74] Budescu D, Por HH, Broomell SB, Smithson M (2014). The interpretation of IPCC probabilistic statements around the world. Nat. Clim. Chang..

[75] Kahan DM (2012). The polarizing impact of science literacy and numeracy on perceived climate change risks. Nat. Clim. Chang..

[76] Leiserowitz A (2006). Climate change risk perception and policy preferences: the role of affect, imagery, and values. Climatic Change.

[77] Van der Linden S (2015). The social-psychological determinants of climate change risk perceptions: towards a comprehensive model. J. Environ. Psychol..

[78] Benjamini Y, Hochberg Y (1995). Controlling the false discovery rate: a practical and powerful approach to multiple testing. J. R. Stat. Soc.: Ser. B (Methodol.).

