# Supplementary material for: Climate change beliefs and their correlates in Latin America
Source: Nat Commun. 2023 Nov 9;14:7241. doi: 10.1038/s41467-023-42729-x (PMC10636181; doi:10.1038/s41467-023-42729-x)
Supplement: Supplementary file 1 — Supplementary Information [file 41467_2023_42729_MOESM1_ESM.pdf]

Climate Change Beliefs and their Correlates in Latin America

October 17, 2023

Contents

1 Descriptive Statistics **2**

1.1 Dependent Variables 2

1.2 Independent Variables: Psychological 11

1.3 Independent Variables: Political ideology 12

1.4 Independent Variables: Socio-Demographic 13

2 Regression Models **16**

3 Robustness Checks **29**

3.1 Multicollinearity 29

3.2 Multiple Hypotheses Testing 29

3.3 Linear Combination of Coefficients 32

3.4 Alternative Specifications 50

1 Descriptive Statistics

This section contains the descriptive statistics of the dependent and independent variables used in the paper. All estimates include weights to adjust sample representativeness.

1.1 Dependent Variables

Table 1: Descriptive statistics - Dimensions of belief in climate change

	Overall	Argentina	Brazil	Chile	Colombia	Ecuador	Mexico	Peru
**Existence of climate change**								
Mean	7.43	7.33	7.18	7.51	7.57	7.50	7.43	7.45
Standard Deviation	1.04	1.16	1.41	0.93	0.83	0.87	0.97	0.97
**Anthropogenic climate change**								
Mean	93.26%	93.81%	91.17%	93.45%	96.09%	93.21%	92.98%	92.10%
Standard Deviation	0.25	0.24	0.28	0.25	0.19	0.25	0.26	0.27
**Consequences of climate change**								
Mean	65.72%	66.46%	56.04%	71.09%	75.12%	65.13%	61.25%	64.95%
Standard Deviation	0.47	0.47	0.50	0.45	0.43	0.48	0.49	0.48
**Belief in climate change (Index)**								
Mean	0.01	0.01	-0.10	0.05	0.11	0.01	-0.01	-0.01
Standard Deviation	0.62	0.63	0.75	0.57	0.50	0.60	0.63	0.65

Notes: This table reports the weighted average (or proportion) and standard deviation for the three dependent variables and the aggregated index. Belief in the *existence of climate change* is an ordinal scale of 0-8. The higher the scale, the greater the respondent is confident that climate change is happening. Belief in the anthropogenic *causes of climate change* is a binary variable equal to 1 for respondents who believe climate change is primarily caused by human activity and eventual natural causes, and 0 for respondents who believe that it is caused primarily by natural causes or neither. Belief in the severity of the *consequences of climate change* is a binary variable equal to 1 for respondents who believe climate change impacts will be negative, and 0 if they believe it will be positive. *Belief in climate change* is an aggregated index of the three dependent variables. It was constructed using factor analysis. *Existence of climate change* was transformed into a binary variable for this purpose (respondents less or equal to 4 on the scale were coded as 0, and 1 otherwise). Descriptive statistics of the factor scores are presented in this table. The higher the factor score, the greater the confidence that climate change is happening, and the higher the perception that it is caused by human activity and that the impacts will be negative.

Table 2: Correlation matrix - Dimensions of belief in climate change

	Existence of climate change	Anthropogenic climate change	Consequences of climate change	Belief in climate change (Index)
**Existence of climate change**	1	.	.	.
**Anthropogenic climate change**	0.22***	1	.	.
**Consequences of climate change**	0.20***	0.18***	1	.
**Belief in climate change (Index)**	0.51***	0.86***	0.52***	1

Notes: This table reports the Pearson correlation coefficients between all dependent variables. Signif. Codes: ***: 0.01, **: 0.05, *: 0.1. p-values from standard two-sided t-tests.

Table 3: Internal consistency of the Index

Variable	Cronbach Alpha [95% CI]
**Belief in climate change (Index)**	0.30 [0.26 ; 0.32]

Notes: This table reports the raw Cronbach Alpha for *Belief in climate change (Index)* and the 95% confidence interval.

Figure 1: Belief in the Existence of Climate Change in Latin America

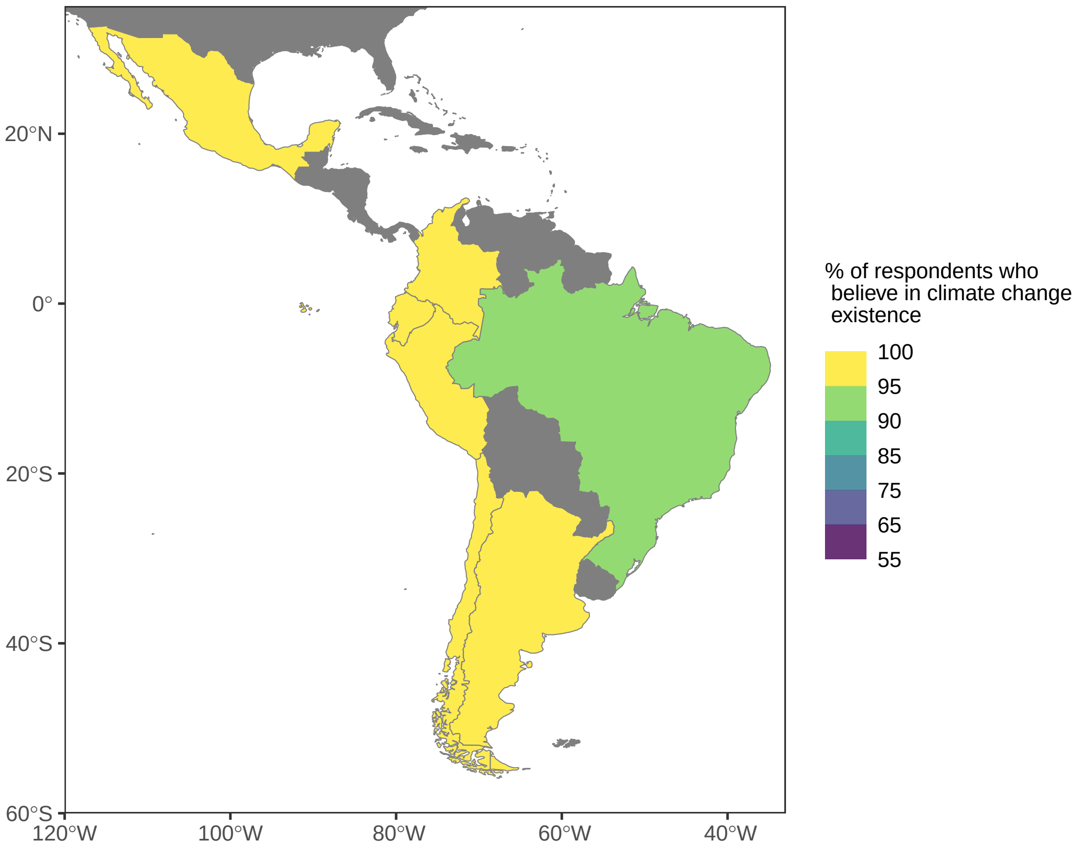

Notes: *Existence of climate change* is an ordinal scale of 0-8. The higher the scale, the greater the respondent is confident that climate change is happening. The weighted proportions presented on the figure are of the respondents who are classified as 5-8 on the scale. Thus, the figure illustrates the % of respondents who believe that the climate is changing. The figure was created by the authors using the *geobr* package in R.

Figure 2: Belief in the Existence of Climate Change in Latin America - Distribution

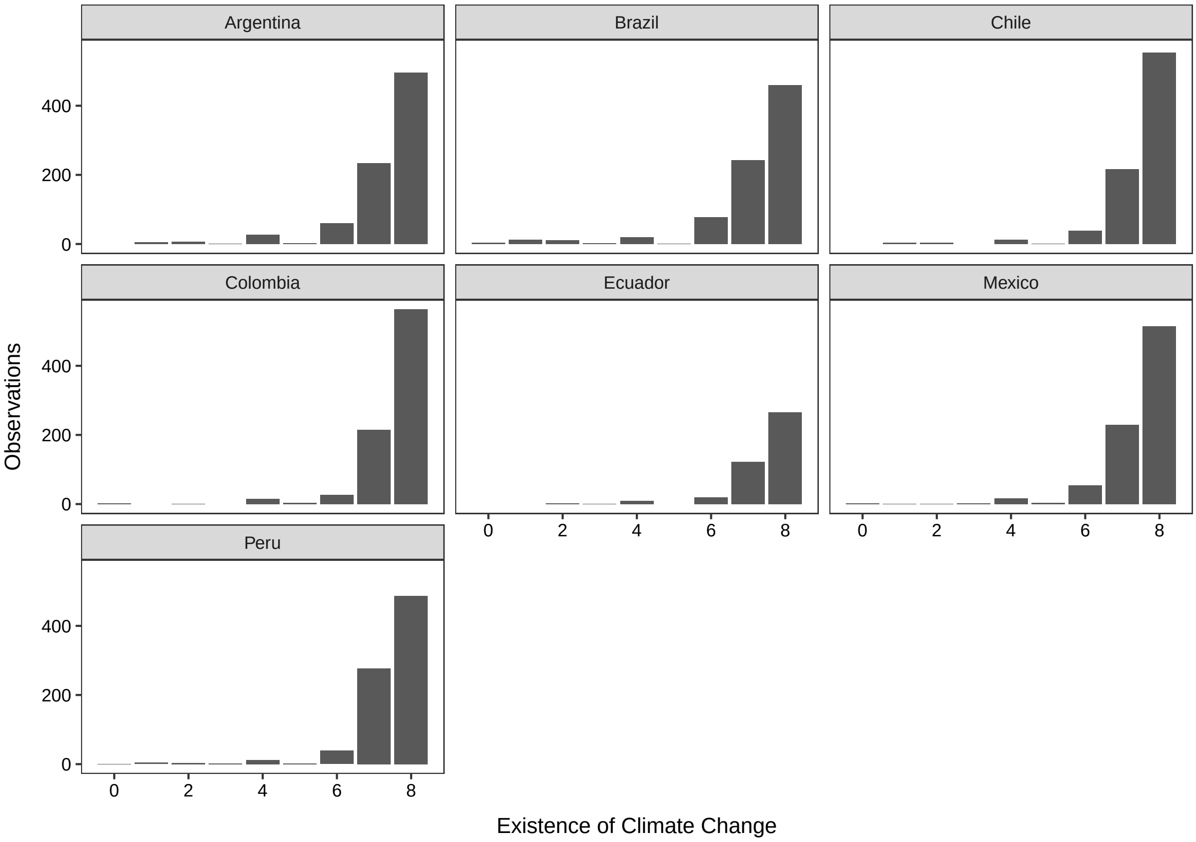

Notes: This plot presents the distribution of the *Existence of climate change* scale by country surveyed. *Existence of climate change* is an ordinal scale of 0-8. The higher the scale, the greater the respondent is confident that climate change is happening.

Figure 3: Belief in the Anthropogenic Causes of Climate Change in Latin America

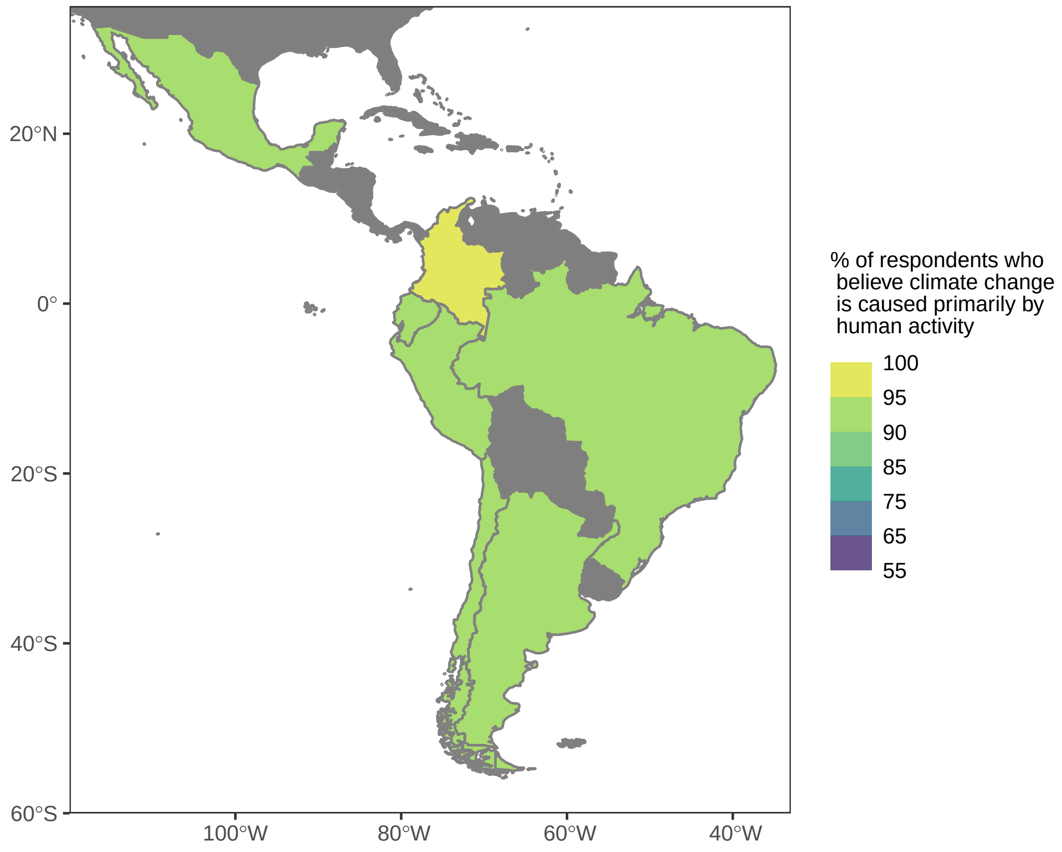

Notes: *Anthropogenic climate change* is a binary variable equal to 1 for respondents who believe climate change is primarily caused by human activity and eventual natural causes, and 0 for respondents who believe that it is caused primarily by natural causes or neither. The weighted proportions presented on the figure are of respondents who are classified as 1. The figure was created by the authors using the *geobr* package in R.

Figure 4: Belief in the Anthropogenic Causes of Climate Change in Latin America - Distribution

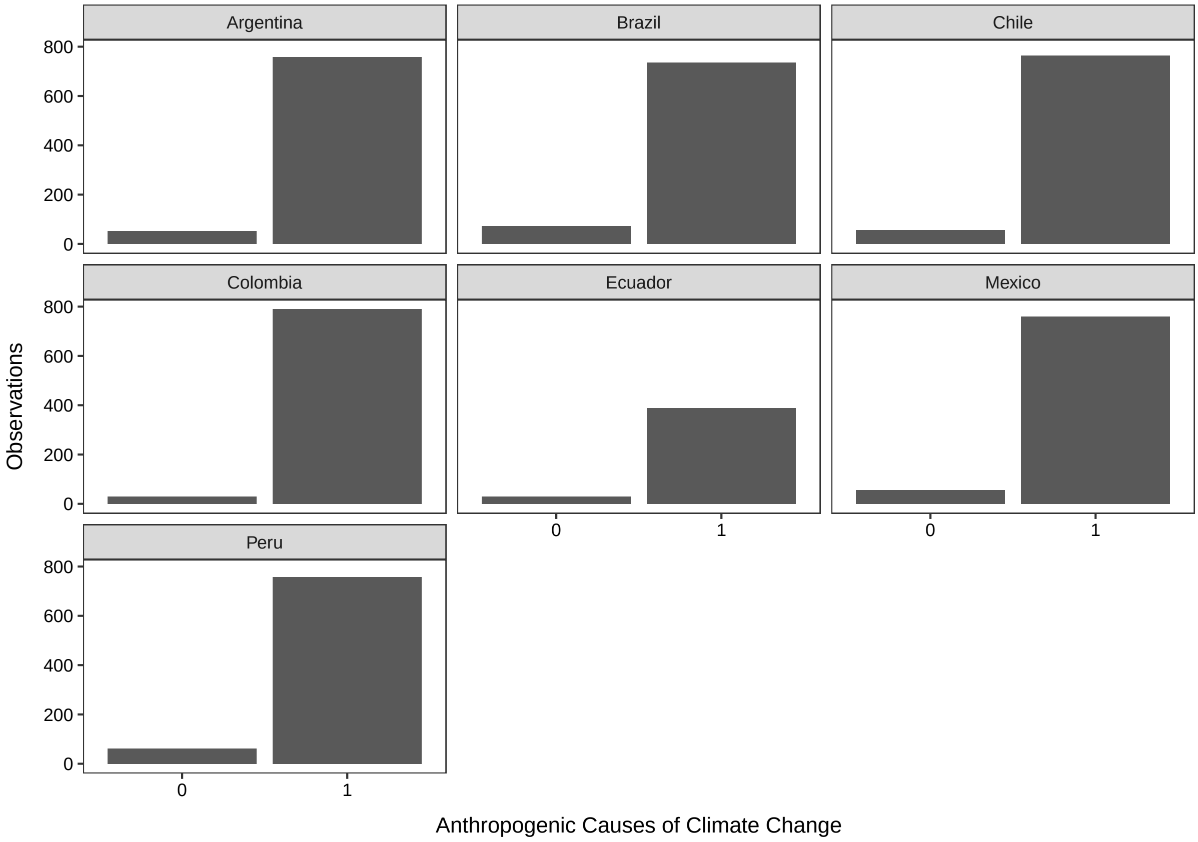

Notes: This plot presents the distribution of the *Anthropogenic climate change* scale by country surveyed. *Anthropogenic climate change* is a binary variable equal to 1 for respondents who believe climate change is primarily caused by human activity and eventual natural causes, and 0 for respondents who believe that it is caused primarily by natural causes or neither. The weighted proportions presented on the figure are of respondents who are classified as 1.

Figure 5: Belief in the Consequences of Climate Change in Latin America

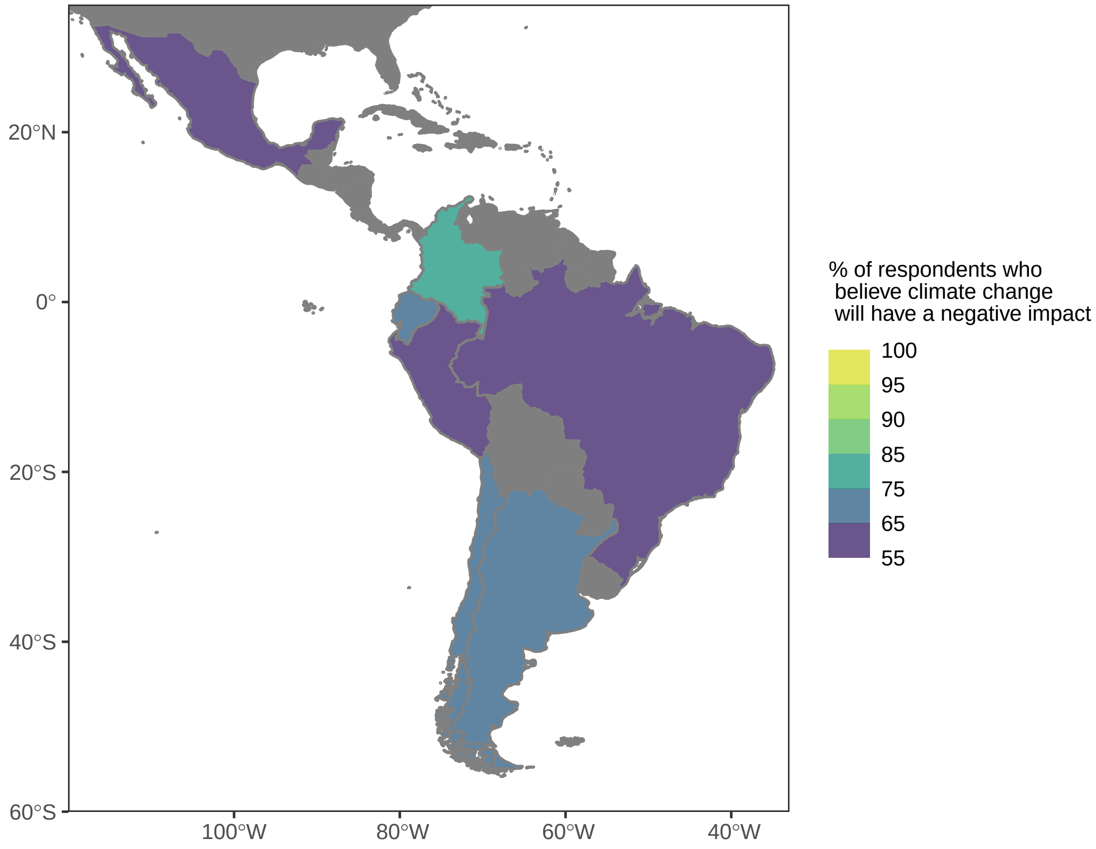

Notes: *Consequences of climate change* is a binary variable equal to 1 for respondents who believe that the impacts of climate change will be negative, and 0 if they believe it will be positive. The weighted proportions presented in the figure are of respondents who are classified as 1. The figure was created by the authors using the *geobr* package in R.

Figure 6: Belief in the Consequences of Climate Change in Latin America - Distribution

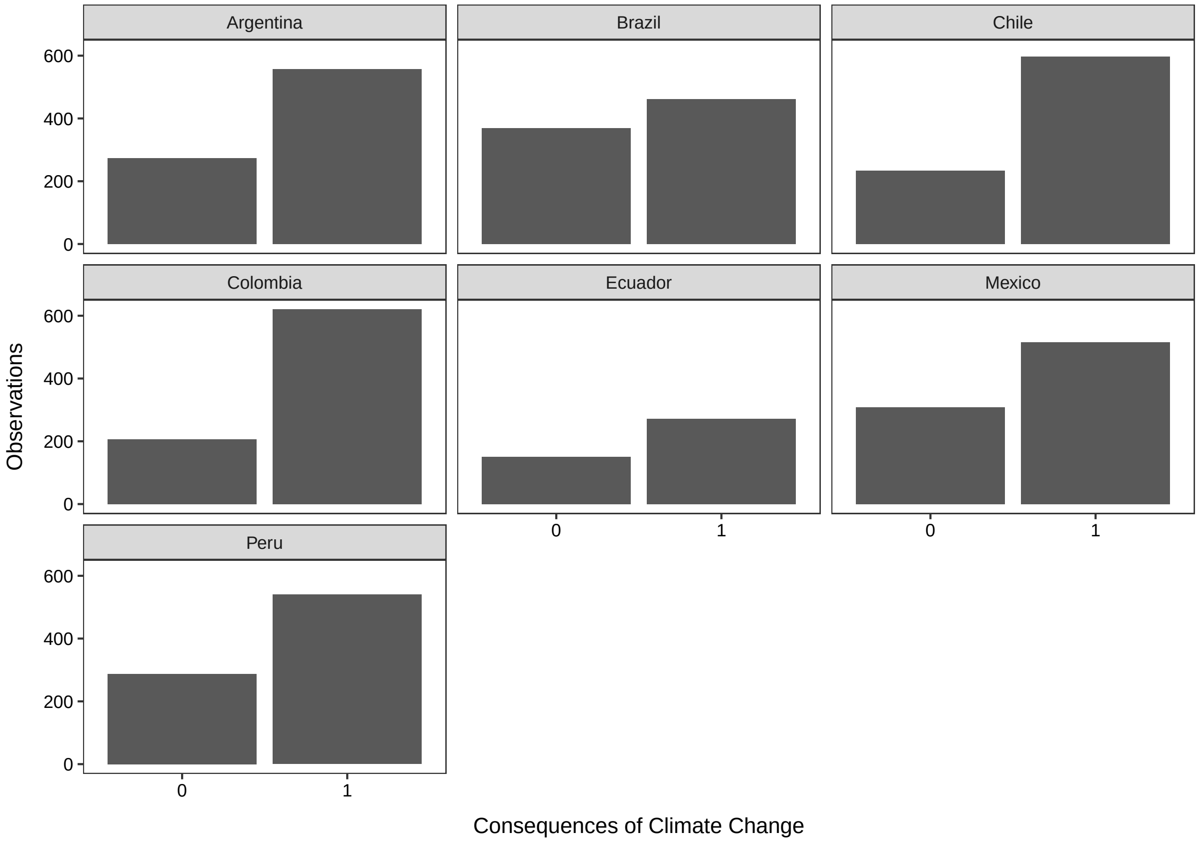

Notes: This plot presents the distribution of the *Consequences of climate change* scale by country surveyed. *Consequences of climate change* is a binary variable equal to 1 for respondents who believe climate change impacts will be negative, and 0 if they believe it will be positive. The weighted proportions presented in the figure are of respondents who are classified as 1.

Figure 7: Belief in Climate Change (Index) in Latin America

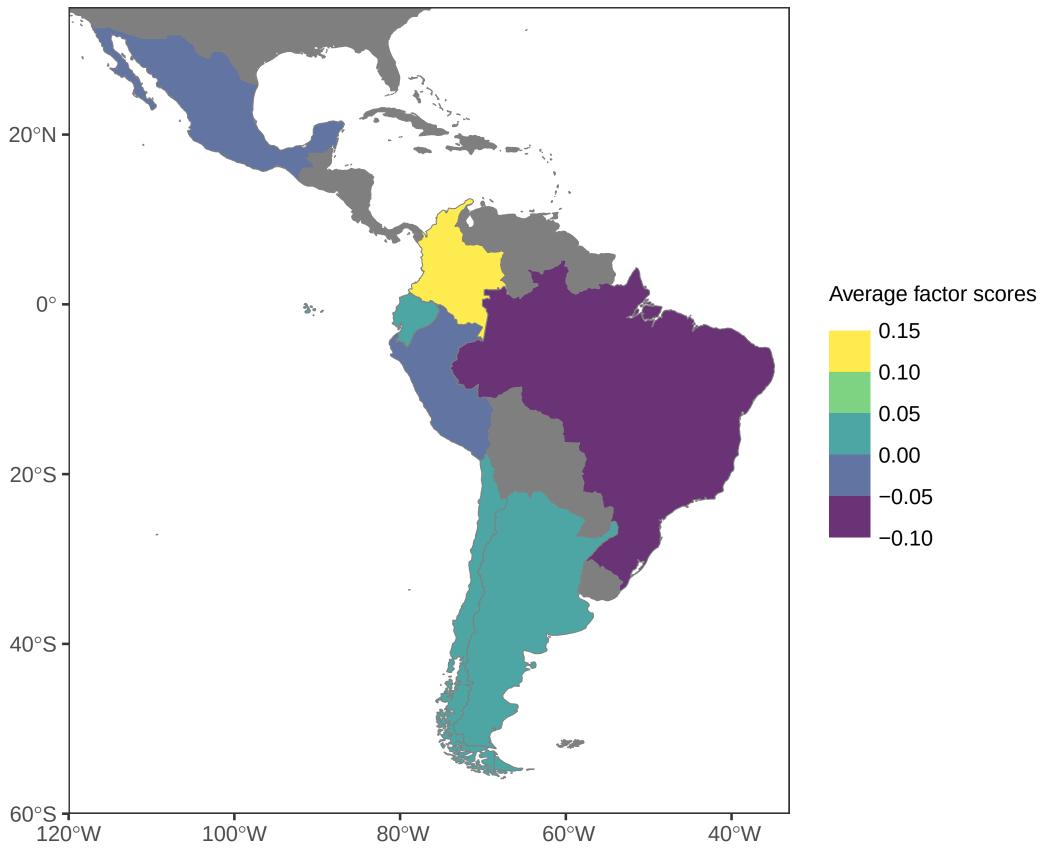

Notes: *Belief in climate change* is an aggregated index of the three dependent variables. It was constructed using factor analysis. *Existence of climate change* was transformed into a binary variable for this purpose. The figure presents the weighted average factor scores for each country. The figure was created by the authors using the *geobr* package in R.

Figure 8: Belief in Climate Change Index in Latin America - Distribution

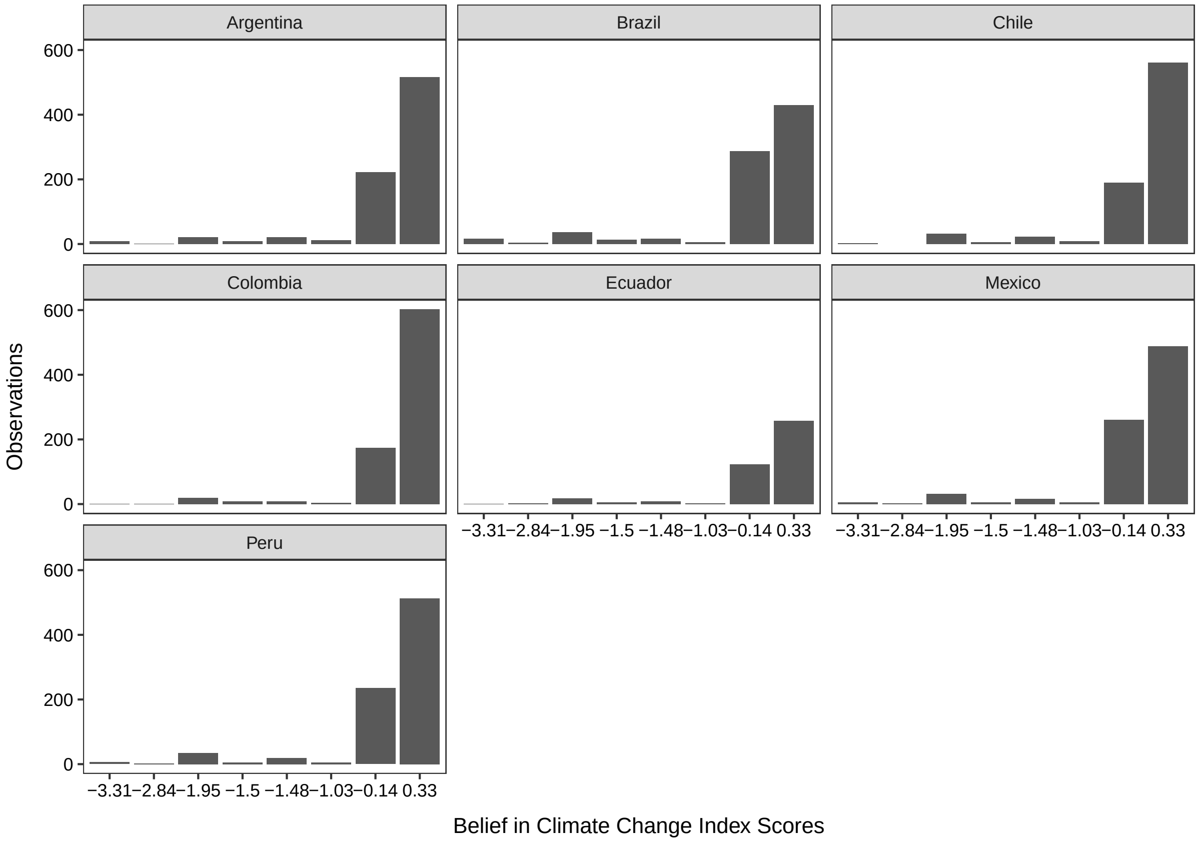

Notes: This plot presents the distribution of the *Belief in climate change Index* average factor scores by country surveyed. *Belief in climate change Index* is an aggregated index of the three dependent variables. It was constructed using factor analysis. *Existence of climate change* was transformed into a binary variable for this purpose.

1.2 Independent Variables: Psychological

Table 4: Descriptive statistics - Psychological variables

	Argentina	Brazil	Chile	Colombia	Ecuador	Mexico	Peru
**Subjective knowledge**							
Nothing	3.95%	3.71%	1.82%	0.47%	0.73%	1.92%	1.12%
A little	22.65%	30.33%	20.65%	17.83%	16.64%	25.50%	15.30%
A moderate amount	65.13%	46.22%	65.37%	70.45%	66.85%	65.18%	70.04%
A lot	8.27%	19.74%	12.16%	11.25%	15.77%	7.40%	13.54%
Do not know	1.39%	1.94%	0.51%	0.93%	0.50%	1.17%	0.38%
**Objective knowledge**							
Mean	4.62	4.40	4.84	5.16	5.00	4.92	4.94
**Scientific consensus**							
Mean	74.47%	69.78%	82.57%	79.14%	80.12%	78.08%	81.10%
**Trust in scientists**							
Mean	86.56%	84.24%	87.46%	87.48%	86.54%	86.06%	83.56%
**The New Ecological Paradigm (NEP)**							
Mean	3.59	3.51	3.64	3.64	3.65	3.58	3.56
**Individualism worldview**							
Mean	2.61	2.61	2.55	2.47	2.50	2.54	2.56
**Egalitarianism worldview**							
Mean	3.21	3.42	3.29	3.26	3.19	3.20	3.13
**Personal experience (extreme weather events)**							
Mean	79.65%	74.58%	83.82%	85.55%	79.95%	85.56%	83.21%

Notes: This table reports the weighted average and proportions for all psychological independent variables by country.

Table 5: Internal consistency - Psychological variables

Variable	Cronbach Alpha [95% CI]
**The New Ecological Paradigm**	0.53 [0.51 ; 0.55]
**Individualism worldview**	0.67 [0.65 ; 0.68]
**Egalitarianism worldview**	0.72 [0.71 ; 0.73]

Notes: This table reports the raw Cronbach Alpha for three psychological independent variables and their respective 95% confidence interval.

Table 6: Correlation matrix - Psychological variables

	Subjective knowledge	Objective knowledge	Scientific consensus	Trust in scientists	The New Ecological Paradigm	Personal experience
**Subjective knowledge**	1.00	.	.	.	.	.
**Objective knowledge**	0.10***	1.00	.	.	.	.
**Scientific consensus**	0.20***	0.22***	1.00	.	.	.
**Trust in scientists**	0.06***	0.18***	0.21***	1.00	.	.
**The New Ecological Paradigm**	0.11***	0.26***	0.20***	0.16***	1.00	.
**Personal experience**	0.10***	0.09***	0.11***	0.04***	0.07***	1.00

Notes: This table reports the pearson correlation coefficents between all psychological independent variables. Signif. Codes: ***: 0.01, **: 0.05, *: 0.1. p-values from standard two-sided t-tests.

1.3 Independent Variables: Political ideology

Table 7: Descriptive statistics - Political ideology variables

	Argentina	Brazil	Chile	Colombia	Ecuador	Mexico	Peru
**Political ideology: Left**							
Mean	60.28%	55.55%	65.86%	73.07%	61.65%	66.29%	47.94%
**Political ideology: Progressive**							
Mean	38.40%	33.02%	33.11%	46.80%	37.79%	32.91%	29.19%

Notes: This table reports the weighted average and proportions for all independent variables.

1.4 Independent Variables: Socio-Demographic

1. **Age**: Age in years;
2. **Female**: Dummy for Female Respondent;
3. **Education**: 3-brackets schooling level;
4. **Income:** 6-brackets income level[1];
5. **Color**: 5-brackets color level;
6. **Religion**: 5-brackets religion levels [2].

Table 8: Descriptive statistics - Socio-Demographic variables

	Argentina (N=830)	Brazil (N=830)	Chile (N=830)	Colombia (N=826)	Ecuador (N=421)	Mexico (N=824)	Peru (N=827)
**Age**							
Mean	42.3	41.0	42.8	39.9	39.0	37.0	41.2
**Female**							
Mean	51.00%	50.70%	51.10%	50.62%	50.44%	51.40%	49.90%
**Education**							
Elementary (Primary) or less	34.68%	43.01%	19.07%	42.32%	10.26%	55.78%	6.79%
High school or equivalent	28.99%	36.14%	49.58%	30.55%	54.74%	23.35%	46.81%
Undergraduate or more	36.33%	20.85%	31.35%	27.13%	35.00%	20.87%	46.41%
**Income**							
0 - 1 minimum wages	16.86%	28.25%	26.94%	30.70%	34.63%	20.94%	30.49%
1 - 2 minimum wages	17.70%	29.01%	26.15%	32.13%	23.49%	5.04%	22.66%
2 - 3 minimum wages	11.15%	14.57%	11.02%	11.54%	8.20%	3.56%	10.78%
3 - 5 minimum wages	13.36%	9.02%	11.13%	7.24%	10.12%	2.28%	11.44%
5 - 10 minimum wages	8.47%	4.89%	7.98%	1.69%	3.26%	2.10%	7.61%
10 minimum wages or more	6.93%	3.32%	2.06%	0.76%	1.18%	27.04%	4.98%
Do not know/ Prefer to not answer	25.53%	10.94%	14.72%	15.93%	19.12%	39.04%	12.04%
**Color**							
Black or *Pardo*	2.94%	53.00%	1.23%	5.76%	2.62%	2.24%	2.56%
Indigenous person	1.49%	1.63%	5.26%	2.90%	2.49%	7.01%	2.04%
Mestizo	24.27%	0.00%	32.03%	49.90%	79.38%	58.43%	69.61%
White	62.16%	43.00%	52.78%	34.24%	12.36%	21.44%	17.86%
Other	9.14%	2.36%	8.70%	7.20%	3.15%	10.88%	7.93%
**Religion**							
Atheist	10.20%	1.85%	7.95%	3.32%	3.13%	5.19%	5.50%
Catholic	51.56%	39.87%	44.47%	53.76%	56.49%	54.19%	60.23%
Evangelical Pentecostal or other evangelical	3.21%	17.86%	4.49%	3.87%	5.04%	3.32%	5.17%
Evangelical Traditional	9.68%	13.20%	11.26%	12.04%	10.91%	5.04%	11.09%
Others/No Relig.	25.35%	27.22%	31.83%	27.02%	24.43%	32.26%	18.00%

Notes: This table reports the weighted proportions for the socio-demographic characteristics of all observations per country. Age is a continuous variable. Thus, the mean represents the weighted average age of all observations per country.

[1] Income levels are reported as minimum wages of each country analyzed.

[2] "Evangelical Pentecostal or others evangelical" includes "Evangelical Pentecostal" and "Evangelical Neo Pentecostal", while "Evangelical Traditional" includes "Evangelical" and "Evangelical Protestant"

Table 9: Brazil's regions

**Region**	(Sample)	(2019 PNAD)
North	7.7%	7.4%
Northeast	25.2%	26.6%
Southeast	45.2%	43.8%
South	14.6%	14.9%
Center-West	7.3%	7.3%

Notes: This table reports the unweighted proportion of observations per region in Brazil compared to the 2019 National Household Sample Survey (PNAD).

Table 10: Sample vs. population distribution by education level and country

	Argentina		Brazil	
	(Sample)	(2010 Census)	(Sample)	(2019 PNAD)
Elementary (Primary) or less	33.61%	41.10%	43.98%	44.30%
High school or equivalent	28.80%	38.90%	35.66%	35.80%
Undergraduate or more	37.59%	20.00%	20.36%	19.90%
	Chile		Colombia	
	(Sample)	(2017 Census)	(Sample)	(2018 Census)
Elementary (Primary) or less	18.92%	23.90%	42.62%	43.00%
High school or equivalent	48.43%	47.20%	29.78%	29.70%
Undergraduate or more	32.65%	28.90%	27.60%	27.40%
	Ecuador		Mexico	
	(Sample)	(2010 Census)	(Sample)	(2020 Census)
Elementary (Primary) or less	10.69%	31.50%	53.88%	54.20%
High school or equivalent	52.02%	43.20%	22.82%	22.50%
Undergraduate or more	37.29%	25.30%	23.30%	23.20%
	Peru			
	(Sample)	(2017 Census)		
Elementary (Primary) or less	5.20%	25.80%		
High school or equivalent	48.13%	38.10%		
Undergraduate or more	46.67%	36.10%		

Notes: This table reports the unweighted proportions for the education level of all observations per country compared to the population. For Brazil, we used the 2019 National Household Sample Survey (PNAD); for the remaining countries, we relied on national censuses.

Table 11: Sample vs. population distribution by sex, age and country

	Argentina				Brazil			
	(Sample)		(2010 Census)		(Sample)		(2019 PNAD)	
**Age**	**Male**	**Female**	**Male**	**Female**	**Male**	**Female**	**Male**	**Female**
18-24	8.7%	8.4%	8.5%	8.5%	9.0%	8.8%	8.9%	8.9%
25-34	11.1%	11.0%	11.1%	11.3%	11.8%	12.5%	12.0%	12.4%
35-44	8.9%	9.4%	8.8%	9.2%	9.6%	10.2%	9.7%	10.3%
45-54	7.6%	7.8%	7.4%	7.9%	7.8%	8.7%	7.8%	8.5%
55+	12.0%	15.1%	12.0%	15.4%	9.6%	11.8%	9.7%	11.8%
**Total**	48.1%	51.9%	48.0%	52.0%	49.3%	50.7%	48.1%	51.9%
	Chile				Colombia			
	(Sample)		(2017 Census)		(Sample)		(2018 Census)	
**Age**	**Male**	**Female**	**Male**	**Female**	**Male**	**Female**	**Male**	**Female**
18-24	7.1%	7.0%	7.2%	7.0%	8.8%	8.7%	8.8%	8.6%
25-34	10.4%	10.8%	10.4%	10.4%	10.8%	11.0%	10.9%	11.1%
35-44	8.8%	9.4%	8.9%	9.2%	9.0%	9.8%	9.0%	9.7%
45-54	8.6%	9.0%	8.5%	9.1%	7.6%	8.8%	7.7%	8.7%
55+	13.0%	15.9%	13.2%	16.0%	11.6%	13.8%	11.6%	13.9%
**Total**	47.8%	52.2%	48.3%	51.7%	48.0%	52.0%	48.0%	52.0%
	Ecuador				Mexico			
	(Sample)		(2010 Census)		(Sample)		(2020 Census)	
**Age**	**Male**	**Female**	**Male**	**Female**	**Male**	**Female**	**Male**	**Female**
18-24	10.2%	10.2%	10.1%	10.2%	8.4%	8.7%	8.4%	8.5%
25-34	12.1%	12.8%	12.2%	12.8%	10.7%	11.4%	10.7%	11.5%
35-44	9.3%	10.0%	9.4%	9.9%	9.6%	10.3%	9.6%	10.4%
45-54	7.4%	7.8%	7.3%	7.6%	8.1%	9.0%	8.2%	9.0%
55+	10.0%	10.2%	9.9%	10.6%	11.0%	12.7%	11.1%	12.7%
**Total**	48.0%	52.0%	48.8%	51.2%	48.0%	52.0%	48.0%	52.0%
	Peru							
	(Sample)		(2017 Census)					
**Age**	**Male**	**Female**	**Male**	**Female**				
18-24	8.5%	8.6%	8.5%	8.7%				
25-34	11.1%	11.6%	11.2%	11.8%				
35-44	9.7%	10.4%	9.8%	10.4%				
45-54	7.6%	8.2%	7.7%	8.2%				
55+	11.6%	12.5%	11.3%	12.4%				
**Total**	48.0%	52.0%	48.5%	51.5%				

Notes: This table reports the uneighted proportions for sex and age of all observations per country compared to the population. For Brazil, we used the 2019 National Household Sample Survey (PNAD); for the remaining countries, we relied on national censuses.

2 Regression Models

Table 12: OLS Results - Correlates of Belief in the Existence of Climate Change

	Dependent Variable: Existence of Climate Change							
	Argentina (i)	Brazil (ii)	Chile (iii)	Colombia (iv)	Ecuador (v)	Mexico (vi)	Peru (vii)	Overall (viii)
Psychological variables								
Subjective knowledge	0.128	0.041	0.060	0.075	0.274***	0.228**	0.197***	0.140***
	(0.094)	(0.079)	(0.062)	(0.080)	(0.094)	(0.092)	(0.060)	(0.033)
Objective knowledge	0.178	0.107	0.092	0.308**	0.219*	0.092	0.153***	0.165***
	(0.122)	(0.121)	(0.056)	(0.149)	(0.111)	(0.094)	(0.057)	(0.038)
Scientific consensus	0.412**	0.828***	0.625***	0.398***	0.320*	0.142	0.354***	0.486***
	(0.177)	(0.211)	(0.154)	(0.153)	(0.182)	(0.116)	(0.137)	(0.067)
Trust in scientists	0.341	0.077	0.155	0.056	0.238	0.248	-0.050	0.122*
	(0.347)	(0.256)	(0.143)	(0.164)	(0.202)	(0.166)	(0.108)	(0.073)
The New Ecological Paradigm (NEP)	0.275**	0.737***	0.422***	-0.026	0.236*	0.281***	0.166**	0.305***
	(0.137)	(0.152)	(0.106)	(0.106)	(0.138)	(0.079)	(0.068)	(0.046)
Individualism worldview	0.029	-0.125	0.062	-0.002	0.018	0.018	0.0001	-0.016
	(0.085)	(0.077)	(0.063)	(0.073)	(0.071)	(0.060)	(0.050)	(0.026)
Egalitarianism worldview	0.039	0.227*	0.003	0.146*	0.036	0.033	0.029	0.067**
	(0.084)	(0.131)	(0.068)	(0.086)	(0.086)	(0.075)	(0.044)	(0.030)
Personal experience (extreme weather events)	-0.011	-0.085	0.506***	0.156	0.106	0.638***	0.378***	0.232***
	(0.159)	(0.149)	(0.162)	(0.170)	(0.146)	(0.242)	(0.137)	(0.060)
Political ideology and Socio-Demographic variables								
Political ideology: Left	-0.072	-0.146	0.078	-0.080	0.074	0.077	-0.002	0.019
	(0.152)	(0.112)	(0.087)	(0.080)	(0.106)	(0.077)	(0.068)	(0.036)
Political ideology: Progressive	0.116	0.221**	0.137*	0.085	0.083	0.082	0.026	0.102***
	(0.118)	(0.106)	(0.074)	(0.072)	(0.101)	(0.068)	(0.051)	(0.032)
Female	-0.160	0.059	0.063	-0.096	0.029	0.059	0.024	0.007
	(0.120)	(0.112)	(0.070)	(0.077)	(0.104)	(0.075)	(0.075)	(0.034)
Education: High school or equivalent	0.171	-0.074	0.063	0.045	0.300	0.047	-0.102	0.013
	(0.182)	(0.126)	(0.122)	(0.097)	(0.225)	(0.088)	(0.136)	(0.047)
Education: Undergraduate or more	0.088	0.002	-0.046	-0.014	0.219	0.048	0.091	0.034
	(0.160)	(0.157)	(0.128)	(0.090)	(0.243)	(0.113)	(0.129)	(0.051)
Religion: Catholic	-0.015	-0.030	-0.081	0.284	-0.028	-0.054	0.136	0.015
	(0.160)	(0.165)	(0.148)	(0.244)	(0.257)	(0.146)	(0.214)	(0.076)
Religion: Evangelical Pentecostal or other evangelical	-0.381	0.301*	-0.022	0.629**	0.016	0.095	0.264	0.138
	(0.531)	(0.180)	(0.193)	(0.292)	(0.495)	(0.223)	(0.216)	(0.110)
Religion: Evangelical Traditional	-0.152	0.301*	-0.112	0.327	0.004	0.104	0.276	0.087
	(0.239)	(0.163)	(0.182)	(0.249)	(0.294)	(0.172)	(0.225)	(0.091)
Religion: Others/No Relig.	0.052	-0.029	-0.197	0.331	0.068	-0.166	0.316	0.005
	(0.171)	(0.173)	(0.157)	(0.245)	(0.277)	(0.157)	(0.216)	(0.080)
Income: 1 - 2 minimum wages	-0.133	0.162	-0.112	0.195	0.258**	0.105	0.063	0.121***
	(0.156)	(0.159)	(0.091)	(0.124)	(0.129)	(0.124)	(0.096)	(0.046)
Income: 2 - 3 minimum wages	-0.444*	0.085	-0.251*	0.293**	0.145	0.127	0.009	0.002
	(0.259)	(0.167)	(0.134)	(0.119)	(0.213)	(0.123)	(0.098)	(0.060)
Income: 3 - 5 minimum wages	-0.136	-0.086	-0.145	0.199	-0.097	-0.734	-0.032	-0.039
	(0.141)	(0.203)	(0.121)	(0.136)	(0.230)	(0.483)	(0.098)	(0.065)
Income: 5 - 10 minimum wages	-0.522**	0.167	0.078	0.378	-0.008	0.150	-0.005	0.010
	(0.242)	(0.288)	(0.137)	(0.278)	(0.310)	(0.420)	(0.122)	(0.080)
Income: 10 minimum wages or more	-0.323	0.227	0.096	0.435	0.015	0.158*	-0.066	0.045
	(0.258)	(0.248)	(0.139)	(0.327)	(0.343)	(0.094)	(0.244)	(0.068)
Income: Do not know/ Prefer to not answer	-0.195	-0.283	-0.268	0.164	0.233	0.179	-0.143	0.028
	(0.164)	(0.263)	(0.192)	(0.174)	(0.172)	(0.111)	(0.142)	(0.060)
Age (Years)	0.007**	0.0006	-0.0008	0.0003	0.003	-0.003	0.004*	0.002
	(0.003)	(0.003)	(0.002)	(0.003)	(0.004)	(0.003)	(0.002)	(0.0010)
Race: Black	-1.39**	-0.118	-0.788	-0.050	-0.396	0.328**	0.043	-0.235**
	(0.638)	(0.166)	(0.867)	(0.147)	(0.456)	(0.162)	(0.178)	(0.105)
Const	4.79***	3.09***	4.44***	5.27***	3.98***	4.49***	4.91***	4.50***
	(0.635)	(0.775)	(0.465)	(0.666)	(1.01)	(0.560)	(0.601)	(0.261)
Fit statistics								
Observations	355	434	434	382	250	485	547	2,887
R^2	0.24024	0.33812	0.33307	0.20002	0.25807	0.20743	0.23666	0.19881
Adjusted R^2	0.18251	0.29756	0.29221	0.14384	0.17527	0.16426	0.20003	0.19180

Notes: Results from ordinary least squares models regressing climate change existence on a set of independent variables and socio-demographic characteristics. Columns labeled with country names are models for each separate country, while column "Overall" includes all observations. Coefficients are changes in the climate change existence scale (i.e., 0-8) given a unit increase in the covariates. The higher the scale, the greater the confidence that climate change is happening. Heteroskedasticity-robust standard errors in parentheses. Reference baseline for Education is "Elementary (Primary) or less", Religion is "Atheist" and Income is "0 – 1 minimum wages". Significance Codes: ***: 0.01, **: 0.05, *: 0.1. p-values from standard two-sided t-tests for the null hypothesis of a zero average parameter using the reported standard errors in parentheses.

Table 13: OLS Results - Correlates of Belief in the Anthropogenic Causes of Climate Change

	Dependent Variable: Anthropogenic Climate Change							
	Argentina (i)	Brazil (ii)	Chile (iii)	Colombia (iv)	Ecuador (v)	Mexico (vi)	Peru (vii)	Overall (viii)
Psychological variables								
Subjective knowledge	0.026	-0.030*	0.043**	0.044**	0.002	-0.032	0.021	0.002
	(0.020)	(0.017)	(0.022)	(0.020)	(0.029)	(0.026)	(0.022)	(0.009)
Objective knowledge	0.019	0.028	0.045*	0.132***	0.027	0.040	0.046**	0.054***
	(0.028)	(0.024)	(0.027)	(0.036)	(0.042)	(0.029)	(0.019)	(0.011)
Scientific consensus	0.025	0.110**	0.033	0.058	0.071	0.092*	0.055	0.070***
	(0.038)	(0.046)	(0.050)	(0.036)	(0.060)	(0.049)	(0.044)	(0.019)
Trust in scientists	0.175**	0.081	0.019	-0.066	0.043	0.061	0.090*	0.056**
	(0.088)	(0.052)	(0.052)	(0.041)	(0.062)	(0.050)	(0.053)	(0.028)
The New Ecological Paradigm (NEP)	0.003	0.075***	-0.004	0.020	0.137***	0.027	0.049*	0.045***
	(0.032)	(0.029)	(0.025)	(0.030)	(0.052)	(0.032)	(0.026)	(0.015)
Individualism worldview	-0.039**	-0.033*	-0.052***	-0.046***	0.004	-0.032	-0.030**	-0.038***
	(0.019)	(0.019)	(0.018)	(0.015)	(0.030)	(0.022)	(0.014)	(0.008)
Egalitarianism worldview	0.028	0.036	0.012	-0.006	-0.014	0.012	0.002	0.010
	(0.017)	(0.024)	(0.020)	(0.013)	(0.028)	(0.022)	(0.016)	(0.008)
Personal experience (extreme weather events)	0.049	0.030	0.021	-0.018	-0.020	0.075	0.028	0.014
	(0.049)	(0.038)	(0.048)	(0.023)	(0.042)	(0.052)	(0.035)	(0.015)
Political ideology and Socio-Demographic variables								
Political ideology: Left	-0.037	0.010	0.006	-0.003	0.072*	0.068**	-0.031	0.015
	(0.025)	(0.028)	(0.027)	(0.025)	(0.039)	(0.028)	(0.025)	(0.012)
Political ideology: Progressive	0.012	-0.030	-0.046*	-0.015	-0.024	-0.036	-0.038	-0.025**
	(0.019)	(0.029)	(0.027)	(0.019)	(0.036)	(0.024)	(0.027)	(0.011)
Female	-0.013	0.006	-0.028	-0.017	0.090***	-0.035	-0.007	0.002
	(0.026)	(0.029)	(0.025)	(0.018)	(0.030)	(0.021)	(0.025)	(0.011)
Education: High school or equivalent	0.065	0.031	-0.030	0.021	0.212**	0.066**	0.132	0.041**
	(0.047)	(0.033)	(0.043)	(0.025)	(0.099)	(0.026)	(0.092)	(0.016)
Education: Undergraduate or more	0.104**	0.037	0.023	0.017	0.212**	0.096***	0.112	0.064***
	(0.045)	(0.039)	(0.045)	(0.023)	(0.099)	(0.028)	(0.085)	(0.016)
Religion: Catholic	-0.047**	-0.034	-0.034	0.116*	-0.010	0.048	-0.031	-0.001
	(0.021)	(0.044)	(0.032)	(0.065)	(0.037)	(0.058)	(0.040)	(0.016)
Religion: Evangelical Pentecostal or other evangelical	-0.058	-0.060	-0.106	0.029	-0.128	0.099	-0.226**	-0.081*
	(0.096)	(0.052)	(0.091)	(0.114)	(0.105)	(0.082)	(0.109)	(0.043)
Religion: Evangelical Traditional	0.016	0.029	0.018	0.080	0.050	0.120**	-0.051	0.030
	(0.037)	(0.043)	(0.047)	(0.068)	(0.055)	(0.059)	(0.053)	(0.020)
Religion: Others/No Relig.	-0.006	-0.060	-0.026	0.060	0.021	0.025	-0.033	-0.011
	(0.023)	(0.045)	(0.033)	(0.070)	(0.040)	(0.058)	(0.045)	(0.018)
Income: 1 - 2 minimum wages	-0.034	0.057	-0.070*	0.012	-0.039	0.044*	0.068*	0.010
	(0.046)	(0.038)	(0.036)	(0.023)	(0.054)	(0.026)	(0.039)	(0.018)
Income: 2 - 3 minimum wages	-0.035	0.034	-0.063	0.016	0.026	-0.031	0.076**	0.015
	(0.048)	(0.041)	(0.039)	(0.028)	(0.058)	(0.063)	(0.035)	(0.018)
Income: 3 - 5 minimum wages	-0.052	-0.026	-0.071*	-0.0004	-0.022	0.063	0.050	-0.004
	(0.042)	(0.061)	(0.039)	(0.044)	(0.082)	(0.070)	(0.039)	(0.021)
Income: 5 - 10 minimum wages	-0.116*	0.095*	-0.073**	-0.174	0.086	0.010	0.059	-0.004
	(0.059)	(0.053)	(0.035)	(0.115)	(0.072)	(0.084)	(0.041)	(0.024)
Income: 10 minimum wages or more	-0.119**	0.008	-0.080**	0.070	0.189**	0.013	0.064	0.006
	(0.053)	(0.071)	(0.036)	(0.063)	(0.090)	(0.026)	(0.066)	(0.020)
Income: Do not know/ Prefer to not answer	-0.019	0.046	-0.081	0.037	-0.017	0.012	0.062	0.007
	(0.042)	(0.047)	(0.056)	(0.033)	(0.060)	(0.032)	(0.046)	(0.018)
Age (Years)	0.001**	0.0007	0.001	-0.001**	0.0001	-0.001	-0.001	-0.0004
	(0.0007)	(0.0008)	(0.0007)	(0.0007)	(0.001)	(0.0006)	(0.0009)	(0.0005)
Race: Black	0.081	-0.054	-0.206	0.069**	0.021	-0.169	0.088	-0.005
	(0.058)	(0.051)	(0.211)	(0.027)	(0.092)	(0.144)	(0.083)	(0.029)
Const	0.559***	0.404***	0.793***	0.505***	0.032	0.593***	0.453***	0.539***
	(0.166)	(0.156)	(0.146)	(0.169)	(0.299)	(0.199)	(0.165)	(0.071)
Fit statistics								
Observations	355	433	431	382	250	485	547	2,883
R^2	0.17620	0.19145	0.13499	0.38617	0.24254	0.16431	0.20623	0.12592
Adjusted R^2	0.11361	0.14179	0.08159	0.34306	0.15800	0.11879	0.16814	0.11827

Notes: Results from ordinary least squares (linear probability) models regressing the perception of climate change causes on a set of independent variables and socio-demographic characteristics. Columns labeled with country names are models for each separate country, while column "Overall" includes all observations. Coefficients multiplied by one hundred are percentage point changes in the probability of believing climate change is mainly caused by human activity given a unit increase in the covariates. Heteroskedasticity-robust standard errors in parentheses. Reference baseline for Education is "Elementary (Primary) or less", Religion is "Atheist" and Income is "0 – 1 minimum wages". Signif. Codes: ***: 0.01, **: 0.05, *: 0.1. p-values from standard two-sided t-tests for the null hypothesis of a zero average parameter using the reported standard errors in parentheses.

Table 14: OLS Results - Correlates of Belief in the Consequences of Climate Change

	Dependent Variable: Consequences of Climate Change							
	Argentina (i)	Brazil (ii)	Chile (iii)	Colombia (iv)	Ecuador (v)	Mexico (vi)	Peru (vii)	Overall (viii)
Psychological variables								
Subjective knowledge	-0.020	0.054*	0.012	-0.041	0.047	0.055	-0.026	0.013
	(0.040)	(0.031)	(0.037)	(0.044)	(0.057)	(0.042)	(0.035)	(0.015)
Objective knowledge	0.103**	0.105***	0.054	0.135***	0.071	-0.032	0.064**	0.067***
	(0.040)	(0.030)	(0.034)	(0.044)	(0.052)	(0.044)	(0.031)	(0.015)
Scientific consensus	0.107	0.052	0.205***	0.011	-0.016	0.068	0.049	0.062**
	(0.078)	(0.060)	(0.077)	(0.064)	(0.092)	(0.069)	(0.066)	(0.027)
Trust in scientists	-0.134	0.027	0.063	-0.081	-0.090	0.180**	0.132*	0.040
	(0.105)	(0.073)	(0.103)	(0.077)	(0.099)	(0.078)	(0.073)	(0.035)
The New Ecological Paradigm (NEP)	0.030	0.092**	0.043	0.089*	0.045	0.073	0.101**	0.081***
	(0.054)	(0.043)	(0.046)	(0.053)	(0.067)	(0.049)	(0.039)	(0.019)
Individualism worldview	-0.143***	-0.104***	-0.106***	-0.157***	-0.080*	-0.109***	-0.096**	-0.115***
	(0.034)	(0.032)	(0.031)	(0.034)	(0.048)	(0.037)	(0.042)	(0.014)
Egalitarianism worldview	0.015	0.009	-0.019	0.039	0.081	-0.011	-0.015	0.005
	(0.037)	(0.040)	(0.038)	(0.032)	(0.057)	(0.035)	(0.036)	(0.016)
Personal experience (extreme weather events)	0.015	0.031	0.100	0.004	0.078	0.026	0.068	0.046*
	(0.070)	(0.061)	(0.073)	(0.057)	(0.081)	(0.082)	(0.063)	(0.027)
Political ideology and Socio-Demographic variables								
Political ideology: Left	-0.082	0.218***	0.110*	0.034	0.041	0.153***	0.034	0.085***
	(0.050)	(0.050)	(0.057)	(0.049)	(0.065)	(0.048)	(0.043)	(0.020)
Political ideology: Progressive	0.015	-0.052	-0.063	-0.023	0.054	0.066	0.003	0.008
	(0.053)	(0.048)	(0.046)	(0.043)	(0.061)	(0.047)	(0.046)	(0.019)
Female	0.014	-0.055	-0.042	-0.028	-0.033	0.017	-0.113**	-0.031
	(0.051)	(0.051)	(0.050)	(0.044)	(0.061)	(0.049)	(0.050)	(0.020)
Education: High school or equivalent	0.075	0.053	-0.048	0.079	0.306**	0.117*	0.019	0.079***
	(0.076)	(0.057)	(0.074)	(0.054)	(0.126)	(0.060)	(0.113)	(0.025)
Education: Undergraduate or more	0.188***	0.125*	-0.010	0.055	0.434***	0.124**	0.037	0.145***
	(0.070)	(0.066)	(0.083)	(0.052)	(0.127)	(0.059)	(0.116)	(0.026)
Religion: Catholic	-0.020	-0.028	-0.090	-0.059	0.051	-0.067	-0.117	-0.067*
	(0.064)	(0.133)	(0.059)	(0.092)	(0.198)	(0.095)	(0.082)	(0.034)
Religion: Evangelical Pentecostal or other evangelical	-0.102	-0.038	-0.162	-0.380**	0.196	-0.022	-0.224*	-0.155***
	(0.137)	(0.141)	(0.131)	(0.188)	(0.220)	(0.149)	(0.136)	(0.052)
Religion: Evangelical Traditional	-0.131	0.068	-0.205**	-0.102	0.096	-0.184	-0.031	-0.082*
	(0.103)	(0.141)	(0.098)	(0.107)	(0.210)	(0.139)	(0.103)	(0.044)
Religion: Others/No Relig.	0.004	0.073	-0.144**	0.013	0.002	-0.062	-0.103	-0.059*
	(0.067)	(0.131)	(0.064)	(0.097)	(0.199)	(0.097)	(0.090)	(0.036)
Income: 1 - 2 minimum wages	-0.091	-0.022	0.020	0.030	0.108	0.092	0.133**	0.061**
	(0.090)	(0.064)	(0.073)	(0.055)	(0.087)	(0.115)	(0.064)	(0.028)
Income: 2 - 3 minimum wages	0.003	0.061	0.043	0.148**	0.095	-0.040	0.179***	0.084***
	(0.093)	(0.075)	(0.080)	(0.060)	(0.114)	(0.118)	(0.068)	(0.032)
Income: 3 - 5 minimum wages	-0.005	-0.090	0.081	0.087	0.264***	-0.177	0.096	0.069**
	(0.086)	(0.087)	(0.078)	(0.085)	(0.092)	(0.147)	(0.070)	(0.032)
Income: 5 - 10 minimum wages	-0.108	0.206**	0.085	-0.069	0.322***	0.014	0.018	0.070
	(0.097)	(0.086)	(0.082)	(0.166)	(0.117)	(0.145)	(0.115)	(0.043)
Income: 10 minimum wages or more	-0.134	0.059	0.133	-0.096	-0.084	0.133**	0.181*	0.082**
	(0.105)	(0.111)	(0.118)	(0.172)	(0.174)	(0.061)	(0.109)	(0.034)
Income: Do not know/ Prefer to not answer	-0.164*	-0.032	0.030	-0.087	-0.017	0.023	0.171**	0.008
	(0.098)	(0.087)	(0.092)	(0.085)	(0.104)	(0.065)	(0.078)	(0.032)
Age (Years)	0.003**	0.003*	0.002	-0.003	-0.002	0.003**	0.001	0.001*
	(0.001)	(0.002)	(0.002)	(0.002)	(0.002)	(0.001)	(0.002)	(0.0007)
Race: Black	0.220***	0.067	-0.130	-0.033	0.114	0.249**	0.060	0.047
	(0.056)	(0.069)	(0.195)	(0.079)	(0.169)	(0.127)	(0.137)	(0.043)
Const	0.655**	-0.295	0.372	0.608**	-0.252	0.091	0.275	0.173
	(0.267)	(0.239)	(0.246)	(0.309)	(0.408)	(0.271)	(0.318)	(0.110)
Fit statistics								
Observations	355	434	434	382	250	485	547	2,887
R^2	0.18102	0.25549	0.20995	0.23642	0.19557	0.18654	0.16093	0.14774
Adjusted R^2	0.11879	0.20987	0.16154	0.18280	0.10578	0.14223	0.12066	0.14029

Notes: Results from ordinary least squares (linear probability) models regressing the perception of climate change consequences on a set of independent variables and socio-demographic characteristics. Columns labeled with country names are models for each separate country, while column "Overall" includes all observations. Coefficients multiplied by one hundred are percentage point changes in the probability of believing climate change impacts will be negative given a unit increase in the covariates. Heteroskedasticity-robust standard errors in parentheses. Reference baseline for Education is "Elementary (Primary) or less", Religion is "Atheist" and Income is "0 – 1 minimum wages". Signif. Codes: ***: 0.01, **: 0.05, *: 0.1. p-values from standard two-sided t-tests for the null hypothesis of a zero average parameter using the reported standard errors in parentheses.

Table 15: OLS Results - Correlates of Belief in Climate Change (Index)

	Dependent Variable: Belief in Climate Change (Index)							
	Argentina (i)	Brazil (ii)	Chile (iii)	Colombia (iv)	Ecuador (v)	Mexico (vi)	Peru (vii)	Overall (viii)
Psychological variables								
Subjective knowledge	0.056	-0.066	0.121*	0.105	0.083	-0.027	0.060	0.024
	(0.091)	(0.069)	(0.069)	(0.068)	(0.106)	(0.107)	(0.078)	(0.033)
Objective knowledge	0.164	0.228**	0.179**	0.553***	0.218	0.126	0.220***	0.254***
	(0.102)	(0.095)	(0.085)	(0.130)	(0.138)	(0.108)	(0.070)	(0.041)
Scientific consensus	0.286*	0.580***	0.355**	0.276**	0.319	0.331*	0.273*	0.370***
	(0.156)	(0.176)	(0.165)	(0.121)	(0.212)	(0.175)	(0.157)	(0.069)
Trust in scientists	0.588*	0.347*	0.102	-0.178	0.071	0.388**	0.388**	0.252**
	(0.356)	(0.203)	(0.155)	(0.132)	(0.221)	(0.191)	(0.194)	(0.102)
The New Ecological Paradigm (NEP)	0.104	0.416***	0.114	0.079	0.472***	0.146	0.248***	0.242***
	(0.122)	(0.116)	(0.086)	(0.100)	(0.163)	(0.112)	(0.096)	(0.050)
Individualism worldview	-0.199***	-0.192***	-0.232***	-0.259***	-0.050	-0.165**	-0.152***	-0.197***
	(0.069)	(0.070)	(0.060)	(0.051)	(0.107)	(0.078)	(0.058)	(0.028)
Egalitarianism worldview	0.124*	0.153	-0.016	0.059	0.067	0.022	0.008	0.055*
	(0.070)	(0.100)	(0.067)	(0.049)	(0.103)	(0.092)	(0.060)	(0.030)
Personal experience (extreme weather events)	0.118	0.088	0.243	-0.036	0.026	0.455*	0.250*	0.134**
	(0.168)	(0.151)	(0.159)	(0.087)	(0.149)	(0.242)	(0.148)	(0.058)
Political ideology and Socio-Demographic variables								
Political ideology: Left	-0.196*	0.126	0.180*	0.016	0.239*	0.326***	-0.079	0.108**
	(0.106)	(0.104)	(0.094)	(0.084)	(0.137)	(0.101)	(0.091)	(0.043)
Political ideology: Progressive	0.059	-0.088	-0.155*	-0.031	-0.033	-0.036	-0.099	-0.052
	(0.080)	(0.106)	(0.091)	(0.067)	(0.126)	(0.084)	(0.096)	(0.040)
Female	-0.081	0.036	-0.097	-0.086	0.234**	-0.087	-0.097	-0.016
	(0.111)	(0.108)	(0.083)	(0.063)	(0.113)	(0.082)	(0.095)	(0.039)
Education: High school or equivalent	0.277*	0.118	-0.140	0.166*	0.967***	0.278***	0.363	0.192***
	(0.167)	(0.120)	(0.138)	(0.094)	(0.337)	(0.102)	(0.322)	(0.057)
Education: Undergraduate or more	0.425***	0.220	-0.014	0.127	1.02***	0.322**	0.345	0.291***
	(0.161)	(0.153)	(0.147)	(0.088)	(0.336)	(0.129)	(0.307)	(0.058)
Religion: Catholic	-0.136	-0.217	-0.120	0.470	-0.006	0.048	-0.145	-0.026
	(0.091)	(0.159)	(0.129)	(0.288)	(0.197)	(0.196)	(0.209)	(0.075)
Religion: Evangelical Pentecostal or other evangelical	-0.460	-0.248	-0.377	0.064	-0.351	0.291	-0.757*	-0.310**
	(0.522)	(0.189)	(0.281)	(0.435)	(0.426)	(0.292)	(0.400)	(0.156)
Religion: Evangelical Traditional	-0.093	0.106	-0.087	0.366	0.115	0.189	-0.096	0.051
	(0.204)	(0.162)	(0.174)	(0.294)	(0.230)	(0.217)	(0.227)	(0.086)
Religion: Others/No Relig.	0.014	-0.227	-0.174	0.364	-0.014	-0.001	-0.130	-0.067
	(0.102)	(0.165)	(0.137)	(0.303)	(0.204)	(0.194)	(0.219)	(0.078)
Income: 1 - 2 minimum wages	-0.206	0.247*	-0.206*	0.099	0.034	0.201*	0.303**	0.101
	(0.166)	(0.140)	(0.112)	(0.089)	(0.196)	(0.119)	(0.140)	(0.062)
Income: 2 - 3 minimum wages	-0.228	0.169	-0.219	0.200*	0.159	-0.099	0.378***	0.092
	(0.189)	(0.159)	(0.138)	(0.103)	(0.228)	(0.200)	(0.130)	(0.068)
Income: 3 - 5 minimum wages	-0.171	-0.080	-0.154	0.109	0.064	-0.075	0.235*	0.040
	(0.153)	(0.214)	(0.133)	(0.167)	(0.255)	(0.337)	(0.135)	(0.073)
Income: 5 - 10 minimum wages	-0.508**	0.511**	-0.154	-0.406	0.417	-0.111	0.179	0.030
	(0.254)	(0.240)	(0.128)	(0.344)	(0.293)	(0.342)	(0.162)	(0.089)
Income: 10 minimum wages or more	-0.567**	0.103	-0.103	0.265	0.627**	0.155	0.276	0.084
	(0.239)	(0.247)	(0.149)	(0.243)	(0.285)	(0.096)	(0.287)	(0.076)
Income: Do not know/ Prefer to not answer	-0.189	0.015	-0.353*	0.073	0.010	0.028	0.263	0.014
	(0.142)	(0.196)	(0.193)	(0.124)	(0.217)	(0.126)	(0.170)	(0.068)
Age (Years)	0.008***	0.004	0.005**	-0.006***	-0.001	-0.0004	-0.003	-0.0002
	(0.003)	(0.003)	(0.002)	(0.002)	(0.004)	(0.003)	(0.004)	(0.002)
Race: Black	-0.457	-0.030	-1.08*	0.149	0.090	-0.222	0.345	-0.038
	(0.396)	(0.183)	(0.560)	(0.110)	(0.405)	(0.381)	(0.234)	(0.102)
Const	-1.80***	-3.15***	-1.03**	-1.84***	-4.16***	-1.88**	-2.17***	-2.15***
	(0.538)	(0.623)	(0.480)	(0.622)	(0.986)	(0.744)	(0.721)	(0.265)
Fit statistics								
Observations	355	433	431	382	250	485	547	2,883
R^2	0.26352	0.31356	0.25622	0.45137	0.28954	0.20840	0.25942	0.21475
Adjusted R^2	0.20756	0.27139	0.21031	0.41285	0.21025	0.16529	0.22388	0.20788

Notes: Results from ordinary least squares models regressing the standardized climate change belief index scores on a set of independent variables and socio-demographic characteristics. Columns labeled with country names are models for each separate country, while column "Overall" includes all observations. Coefficients are changes in the climate change belief index given a standard deviation increase in the covariates. The higher the index score, the greater the confidence that climate change is happening, and the higher the perception that it is caused by human activity and that the impacts will be negative. Heteroskedasticity-robust standard errors in parentheses. Reference baseline for Education is "Elementary (Primary) or less", Religion is "Atheist" and Income is "0 – 1 minimum wages". Signif. Codes: ***: 0.01, **: 0.05, *: 0.1. p-values from standard two-sided t-tests for the null hypothesis of a zero average parameter using the reported standard errors in parentheses.

Figure 9: Correlates of Belief in the Existence of Climate Change in Latin America

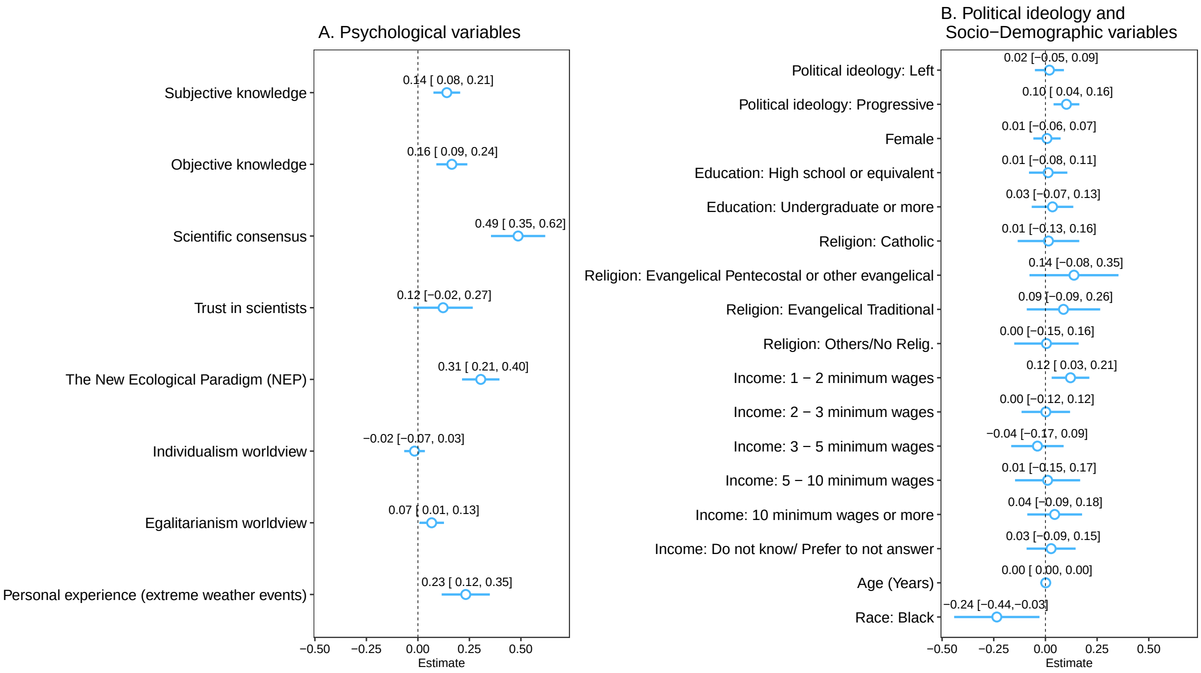

Notes: Results from an ordinary least squares model regressing climate change existence on a set of independent variables (A) and socio-demographic characteristics (B). Respondents from all countries (n = 2,887 observations) are included in the model. For each independent variable, point estimates of the coefficients and confidence intervals, in brackets, are reported. Coefficients are changes in the climate change existence scale (i.e., 0-8) given a unit increase in the covariates. The higher the scale, the greater the confidence that climate change is happening. The width of the confidence intervals for each coefficient is 95% with heteroskedasticity-robust standard errors. Reference baseline for Education is "Elementary (Primary) or less", Religion is "Atheist" and Income is "0 – 1 minimum wages".

Figure 10: Correlates of Belief in the Anthropogenic Causes of Climate Change in Latin America

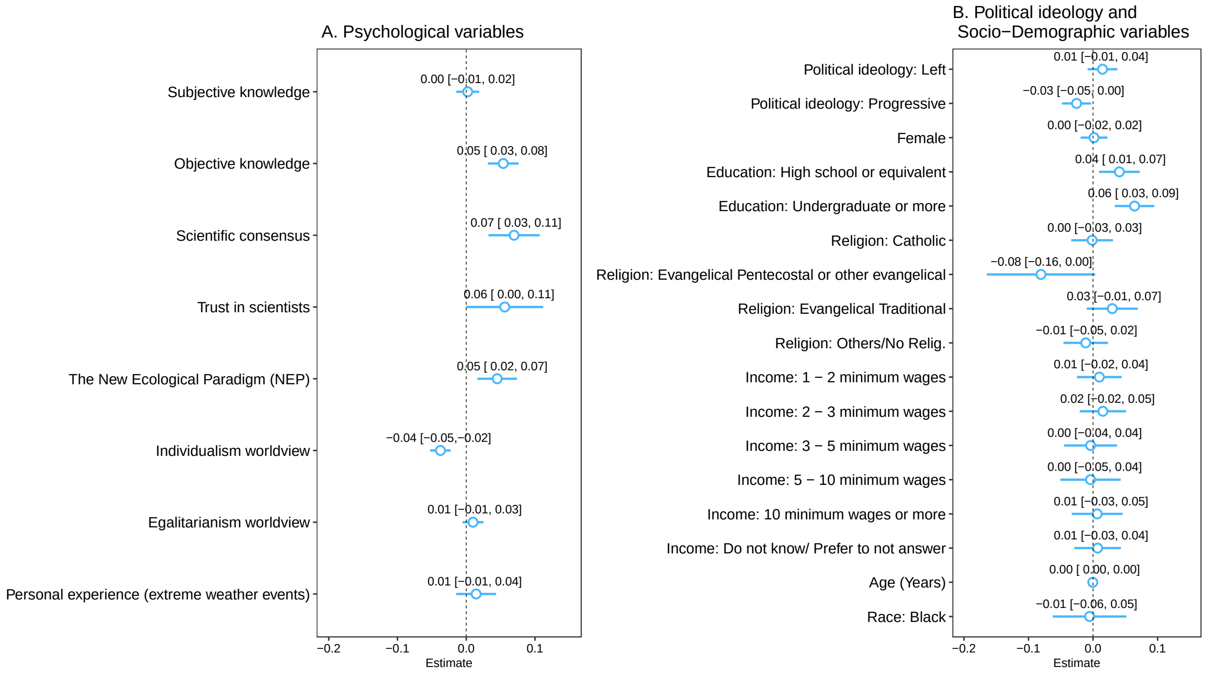

Notes: Results from an ordinary least squares (linear probability) model regressing the perception of climate change anthropogenic causes on a set of independent variables (A) and socio-demographic characteristics (B). Respondents from all countries (n = 2,883 observations) are included in the model. For each independent variable, point estimates of the coefficients and confidence intervals, in brackets, are reported. Coefficients multiplied by one hundred are percentage point changes in the probability of believing climate change is mainly caused by human activity given a unit increase in the covariates. The width of the confidence intervals for each coefficient is 95% with heteroskedasticity-robust standard errors. Reference baseline for Education is "Elementary (Primary) or less", Religion is "Atheist" and Income is "0 – 1 minimum wages".

Figure 11: Correlates of Belief in the Consequences of Climate Change in Latin America

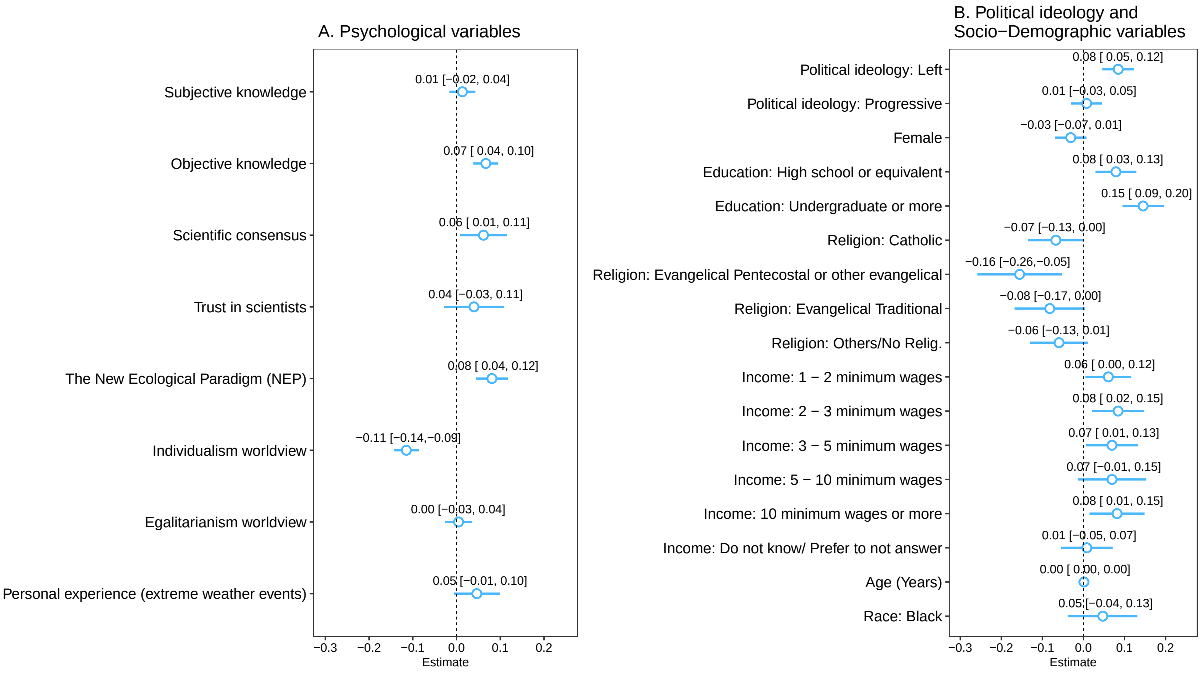

Notes: Results from an ordinary least squares (linear probability) model regressing the perception of climate change consequences on a set of independent variables (A) and socio-demographic characteristics (B). Respondents from all countries (n= 2,887 observations) are included in the model. For each independent variable, point estimates of the coefficients and confidence intervals, in brackets, are reported. Coefficients multiplied by one hundred are percentage point changes in the probability of believing climate change consequences will be negative given a unit increase in the covariates. The width of the confidence intervals for each coefficient is 95% with heteroskedasticity-robust standard errors. Reference baseline for Education is “Elementary (Primary) or less”, Religion is “Atheist” and Income is “0 – 1 minimum wages”.

Figure 12: OLS Results - Correlates of Belief in the Anthropogenic Causes and Consequences of Climate Change in Latin America

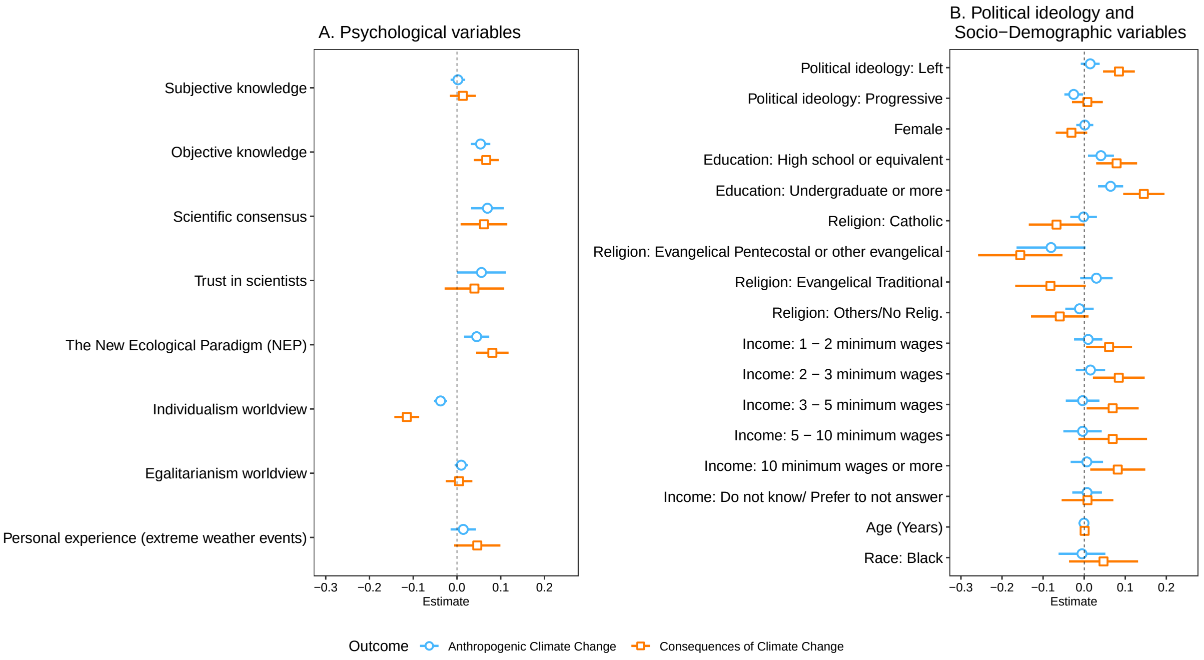

Notes: Results from two ordinary least squares (linear probability) models regressing the perception of climate change causes (blue) and impacts (orange) on the same set of independent variables (A) and socio-demographic characteristics (B). Respondents from all countries are included in each model (n = 2,883 observations for the anthropogenic causes and n= 2,887 observations for the consequences of climate change). For each independent variable, point estimates of the coefficients and confidence intervals, in brackets, are reported. Blue coefficients multiplied by one hundred are percentage point changes in the probability of believing climate change is mainly caused by human activity given a unit increase in the covariates. Orange coefficients multiplied by one hundred are percentage point changes in the probability of believing climate change impacts will be negative given a unit increase in the covariates. The width of the confidence intervals for each coefficient is 95% with heteroskedasticity-robust standard errors. Reference baseline for Education is “Elementary (Primary) or less”, Religion is “Atheist” and Income is “0 – 1 minimum wages”.

Figure 13: OLS Results - Correlates of Belief in Climate Change (Index) in Latin America

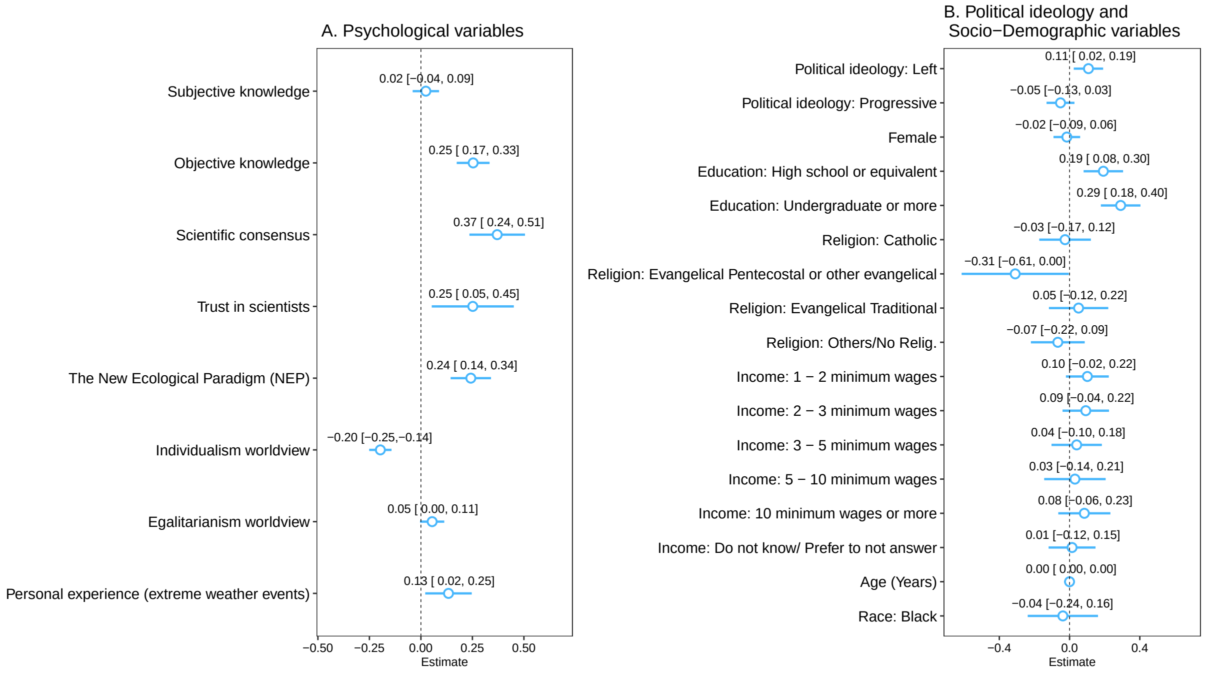

Notes: Results from ordinary least squares models regressing the standardized climate change belief index scores on a set of independent variables (A) and socio-demographic characteristics (B). Respondents from all countries (n = 2,883 observations) are included in the model. For each independent variable, point estimates of the coefficients and confidence intervals, in brackets, are reported. Coefficients are changes in the climate change belief index given a standard deviation increase in the covariates. The higher the index score, the greater the confidence that climate change is happening, and the higher the perception that it is caused by human activity and that the impacts will be negative. The width of the confidence intervals for each coefficient is 95% with heteroskedasticity-robust standard errors. Reference baseline for Education is "Elementary (Primary) or less", Religion is "Atheist" and Income is "0 – 1 minimum wages".

Figure 14: OLS Results - Correlations of Belief in the Existence of Climate Change per Country

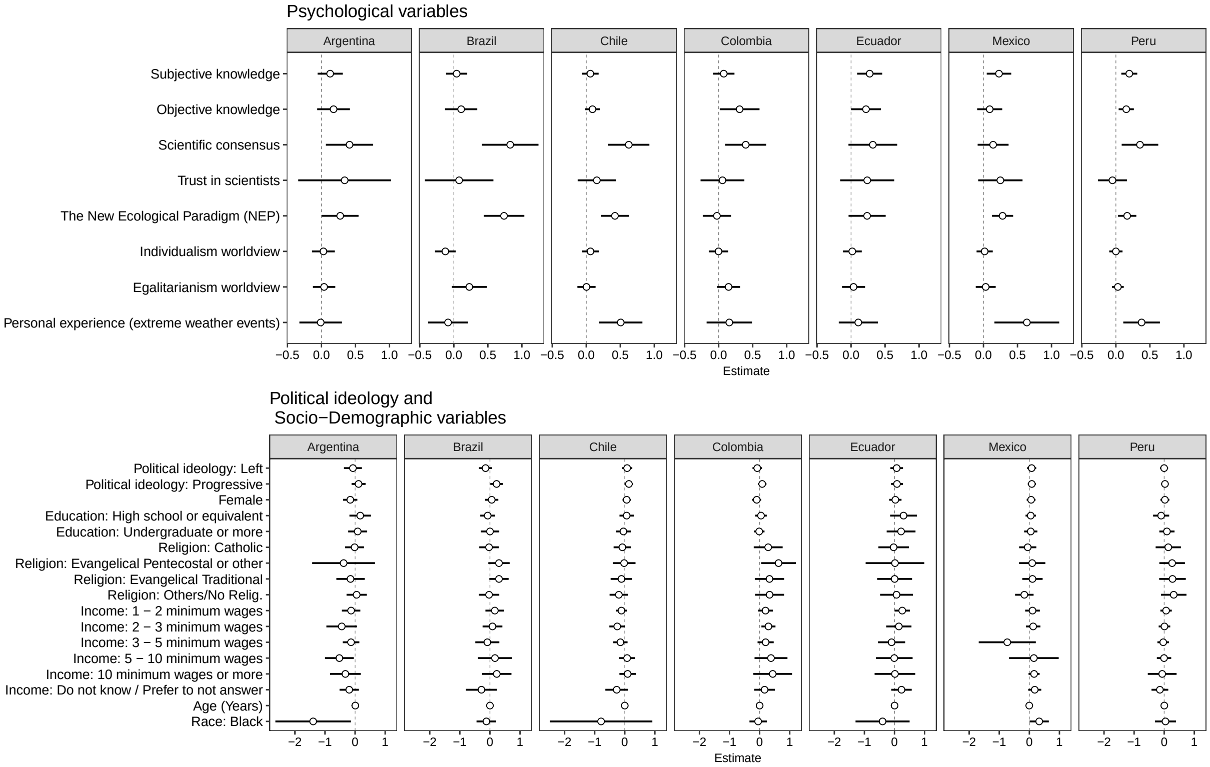

Notes: Results from seven ordinary least squares models regressing climate change existence on a set of independent variables and socio-demographic characteristics. Each column represents a separate model for observations from each country (n = 355 observations for Argentina, n = 434 for Brazil, n = 434 for Chile, n = 382 for Colombia, n = 250 for Ecuador, n = 485 for Mexico and n = 547 for Peru). Coefficients are increases in the climate change existence scale (i.e., 0-8) given a unit increase in the covariates. The higher the scale, the greater the confidence that climate change is happening. The width of the confidence intervals for each coefficient is 95% with heteroskedasticity-robust standard errors. Reference baseline for Education is "Elementary (Primary) or less", Religion is "Atheist" and Income is "0 – 1 minimum wages".

Figure 15: OLS Results - Correlates of Belief in the Anthropogenic Causes of Climate Change per Country

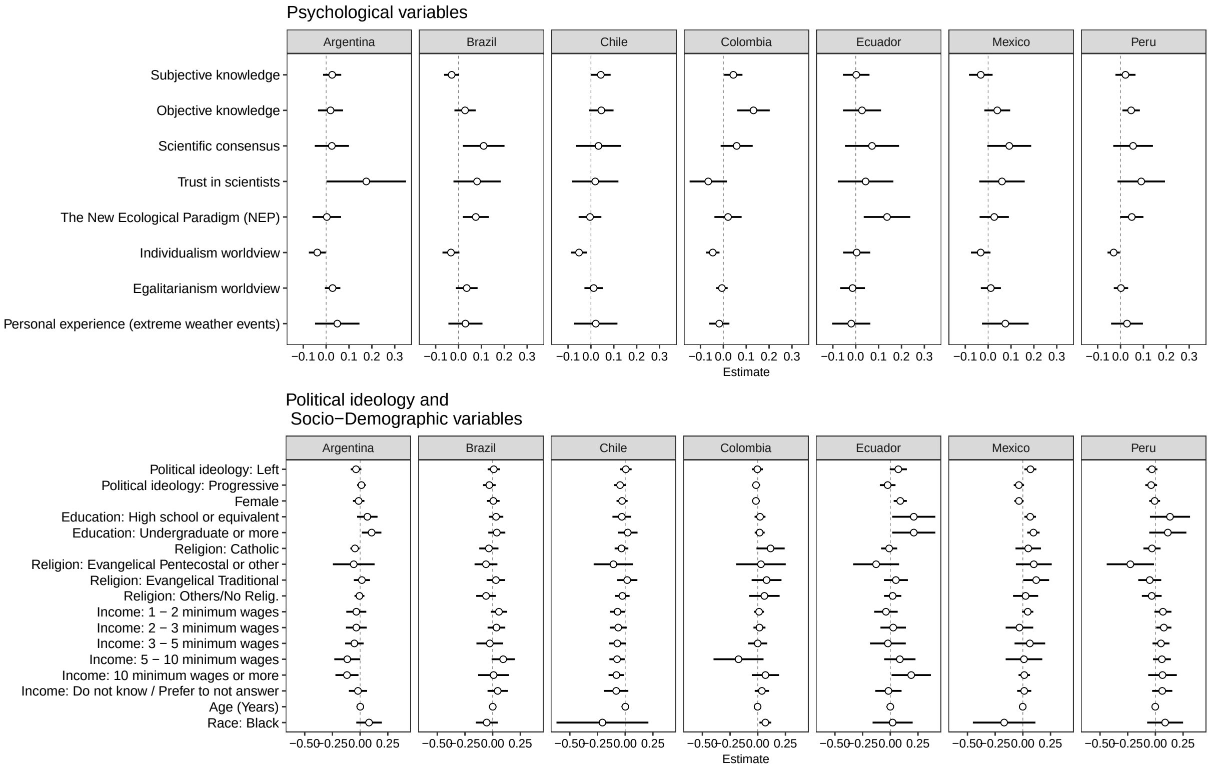

Notes: Results from seven ordinary least squares (linear probability) models regressing the perception of climate change anthropogenic causes on a set of independent variables and socio-demographic characteristics. Each column represents a separate model for observations from each country (n = 355 observations for Argentina, n = 433 for Brazil, n = 431 for Chile, n = 382 for Colombia, n = 250 for Ecuador, n = 485 for Mexico and n = 547 for Peru). Coefficients multiplied by one hundred are percentage point increases in the probability of believing climate change is mainly caused by human activity given a unit increase in the covariates. The width of the confidence intervals for each coefficient is 95% with heteroskedasticity-robust standard errors. Reference baseline for Education is “Elementary (Primary) or less”, Religion is “Atheist” and Income is “0 – 1 minimum wages”.

Figure 16: OLS Results - Correlates of Belief in the Consequences of Climate Change per Country

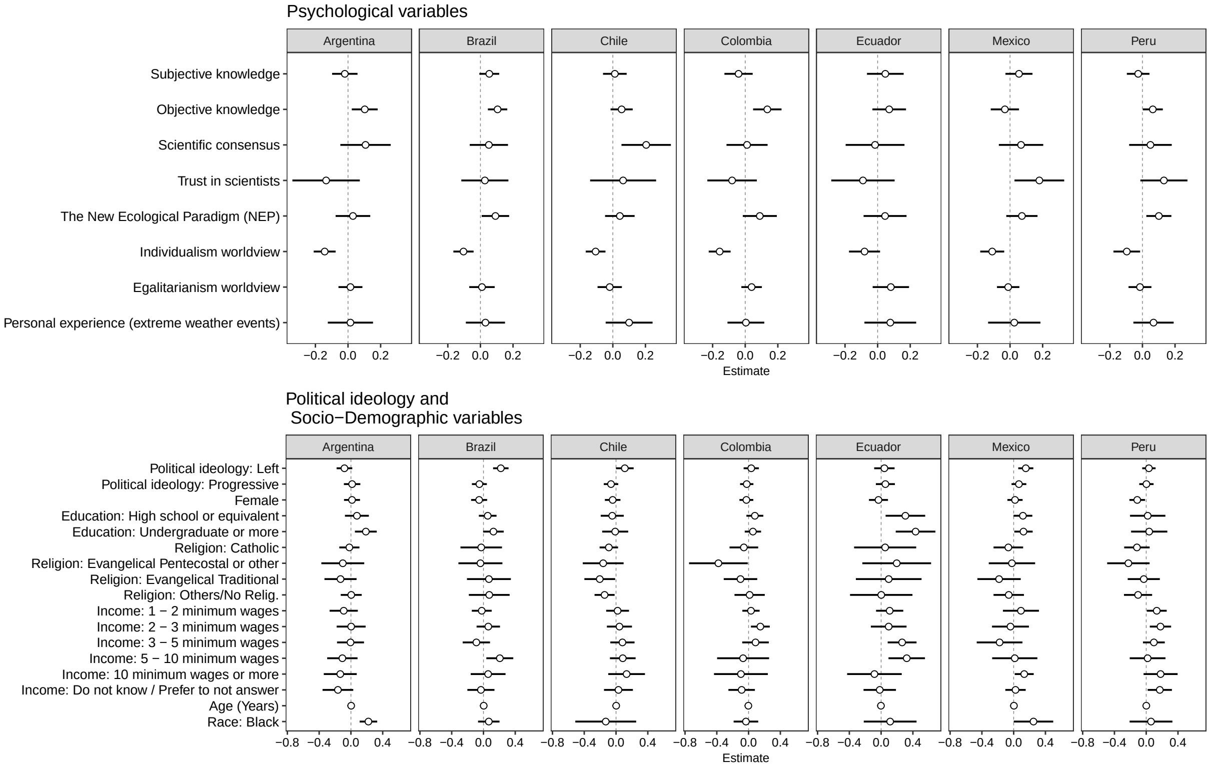

Notes: Results from seven ordinary least squares (linear probability) models regressing the perception of climate change consequences on a set of independent variables and socio-demographic characteristics. Each column represents a separate model for observations from each country (n = 355 observations for Argentina, n = 434 for Brazil, n = 434 for Chile, n = 382 for Colombia, n = 250 for Ecuador, n = 485 for Mexico and n = 547 for Peru). Coefficients multiplied by one hundred are percentage point increases in the probability of believing climate change impacts will be negative given a unit increase in the covariates. The width of the confidence intervals for each coefficient is 95% with heteroskedasticity-robust standard errors. Reference baseline for Education is "Elementary (Primary) or less", Religion is "Atheist" and Income is "0 – 1 minimum wages".

Figure 17: OLS Results - Correlates of Belief in Climate Change (Index) per Country

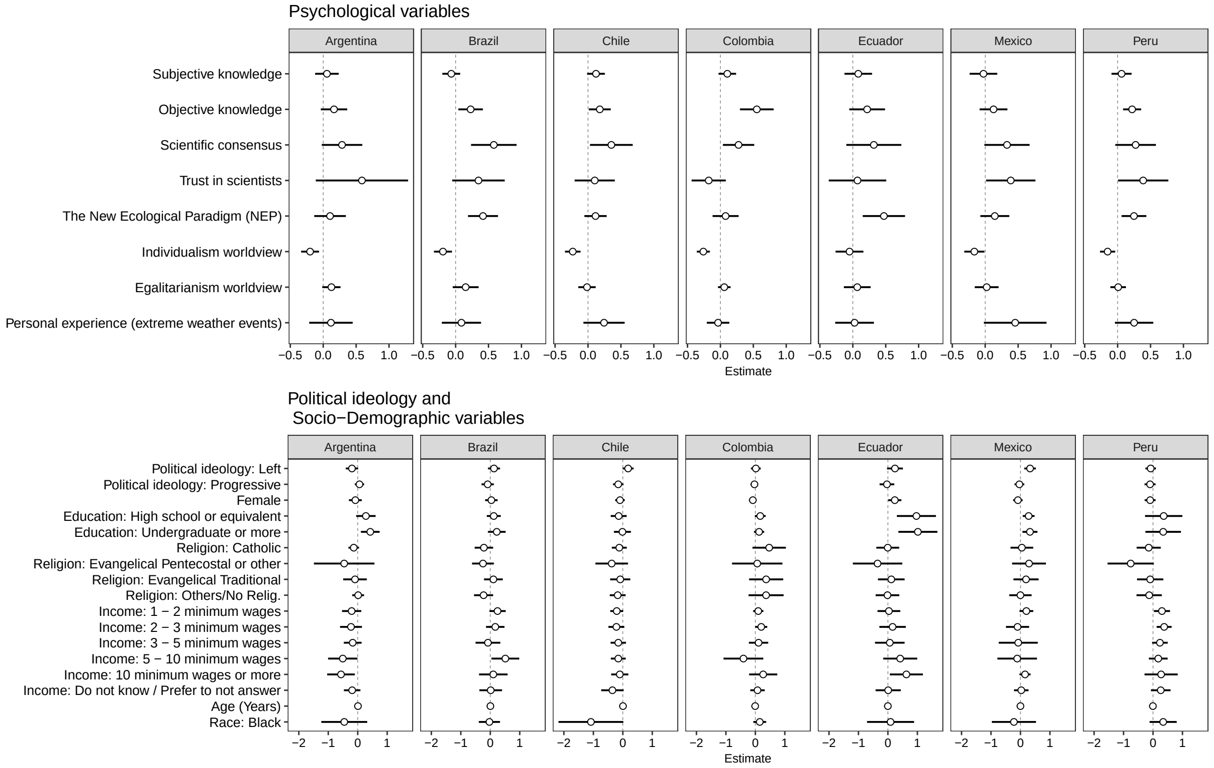

Notes: Results from seven ordinary least squares models regressing the climate change belief index on a set of independent variables and socio-demographic characteristics. Each column represents a separate model for observations from each country (n = 355 observations for Argentina, n = 433 for Brazil, n = 431 for Chile, n = 382 for Colombia, n = 250 for Ecuador, n = 485 for Mexico and n = 547 for Peru). Coefficients are changes in the climate change belief index given a standard deviation increase in the covariates. The higher the index score, the greater the confidence that climate change is happening, and the higher the perception that it is caused by human activity and that the impacts will be negative. The width of the confidence intervals for each coefficient is 95% with heteroskedasticity-robust standard errors. Reference baseline for Education is “Elementary (Primary) or less”, Religion is “Atheist” and Income is “0 – 1 minimum wages”.

3 Robustness Checks

3.1 Multicollinearity

Table 16: Multicollinearity Assessment

Term	VIF	VIF CI low	VIF CI high	Increased SE	Tolerance	Tolerance CI low	Tolerance CI high
Psychological variables							
Subjective knowledge	1.08	1.05	1.14	1.04	0.92	0.88	0.95
Objective knowledge	1.18	1.14	1.24	1.09	0.85	0.81	0.88
Scientific consensus	1.17	1.12	1.22	1.08	0.86	0.82	0.89
Trust in scientists	1.11	1.07	1.17	1.05	0.90	0.86	0.93
The New Ecological Paradigm	1.20	1.15	1.25	1.09	0.84	0.80	0.87
Individualism worldview	1.11	1.08	1.17	1.06	0.90	0.86	0.93
Egalitarianism worldview	1.22	1.18	1.28	1.11	0.82	0.78	0.85
Personal experience	1.02	1.00	1.13	1.01	0.98	0.89	1.00
Political ideology and Socio-Demographic variables							
Political Ideology: Left	1.13	1.09	1.18	1.06	0.89	0.85	0.92
Political Ideology: Progressive	1.10	1.06	1.15	1.05	0.91	0.87	0.94
Female	1.14	1.10	1.20	1.07	0.88	0.84	0.91
Education	1.38	1.32	1.44	1.17	0.73	0.69	0.76
Religion	1.20	1.15	1.25	1.09	0.84	0.80	0.87
Income	1.43	1.37	1.51	1.20	0.70	0.66	0.73
Age (Years)	1.20	1.16	1.26	1.10	0.83	0.79	0.86
Race: Black	1.03	1.01	1.11	1.02	0.97	0.90	0.99

Notes: This table reports a multicollinearity assessment. The columns represent the variance inflation factor (VIF) and the associated 95% confidence intervals; the factor by which the standard error is increased due to possible correlation with other terms; the tolerance values including their 95% confidence intervals. Tolerance is calculated as $\frac{1}{VIF}$.

3.2 Multiple Hypotheses Testing

Table 17: Test of Joint Significance - All independent variables

Model	Res.Df	Df	F	P-value
Existence of climate change	2861	25	28.40	< 0.001
Anthropogenic climate change	2857	25	16.46	< 0.001
Consequences of climate change	2861	25	19.84	< 0.001
Belief in climate change (Index)	2857	25	31.25	< 0.001

Notes: This table reports the results of an one-sided F-test to evaluate if the all independent variables are jointly significant in each of the four main OLS models estimated for the whole sample.

Table 18: Test of Joint Significance - Psychological variables

Model	Res.Df	Df	F	P-value
Existence of climate change	2861	8	25.98	< 0.001
Anthropogenic climate change	2857	8	13.91	< 0.001
Consequences of climate change	2861	8	22.35	< 0.001
Belief in climate change (Index)	2857	8	26.37	< 0.001

Notes: This table reports the results of an one-sided F-test to evaluate if the psychological variables are jointly significant in each of the four main OLS models estimated for the whole sample.

Table 19: Test of Joint Significance - Political variables

Model	Res.Df	Df	F	P-value
Existence of climate change	2861	2	5.54	0.004
Anthropogenic climate change	2857	2	3.41	0.03
Consequences of climate change	2861	2	9.35	< 0.001
Belief in climate change (Index)	2857	2	4.13	0.02

Notes: This table reports the results of an one-sided F-test to evaluate if the political variables are jointly significant in each of the four main OLS models estimated for the whole sample.

Table 20: Test of Joint Significance - Socio-demographic variables

Model	Res.Df	Df	F	P-value
Existence of climate change	2861	15	1.98	0.01
Anthropogenic climate change	2857	15	1.83	0.03
Consequences of climate change	2861	15	5.60	< 0.001
Belief in climate change (Index)	2857	15	2.79	< 0.001

Notes: This table reports the results of an one-sided F-test to evaluate if the political variables are jointly significant in each of the four main OLS models estimated for the whole sample.

Table 21: Test of Joint Significance - Statistically insignificant variables

Model	Res.Df	Df	F	P-value
Existence of climate change	2861	16	0.85	0.63
Anthropogenic climate change	2857	16	1.28	0.20
Consequences of climate change	2861	13	1.66	0.06
Belief in climate change (Index)	2857	15	1.17	0.29

Notes: This table reports the results of an one-sided F-test to evaluate if the statistically insignificant variables at the 95% level are jointly significant in each of the four main OLS models estimated for the whole sample.

Table 22: Benjamini-Hochberg adjusted p-values for multiple hypotheses testing

Model:	Existence				Anthropogenic Causes				Consequences				Belief (Index)			
	Standard	Signif.	Adjusted	Signif.	Standard	Signif.	Adjusted	Signif.	Standard	Signif.	Adjusted	Signif.	Standard	Signif.	Adjusted	Signif.
Psychological variables																
Subjective knowledge	0.0000	***	0.0000	***	0.8027	-	0.9088	-	0.3805	-	0.3779	-	0.4632	-	0.5604	-
Objective knowledge	0.0000	***	0.0000	***	0.0000	***	0.0000	***	0.0000	***	0.0000	***	0.0000	***	0.0000	***
Scientific consensus	0.0000	***	0.0000	***	0.0003	***	0.0000	***	0.0236	**	0.0259	**	0.0000	***	0.0000	***
Trust in scientists	0.0964	*	0.0542	*	0.0499	**	0.0008	***	0.2514	-	0.1842	-	0.0133	**	0.0000	***
The New Ecological Paradigm (NEP)	0.0000	***	0.0000	***	0.0022	***	0.0000	***	0.0000	***	0.0000	***	0.0000	***	0.0000	***
Individualism worldview	0.5223	-	0.7368	-	0.0000	***	0.0000	***	0.0000	***	0.0000	***	0.0000	***	0.0000	***
Egalitarianism worldview	0.0284	**	0.0223	**	0.2058	-	0.3232	-	0.7645	-	0.7459	-	0.0688	*	0.0756	*
Personal experience (extreme weather events)	0.0001	***	0.0000	***	0.3299	-	0.4574	-	0.0849	*	0.0848	*	0.0202	**	0.0114	**
Political ideology and Socio-Demographic variables																
Political ideology: Left	0.5911	-	0.7700	-	0.2113	-	0.2729	-	0.0000	***	0.0000	***	0.0118	**	0.0046	***
Political ideology: Progressive	0.0014	***	0.0097	***	0.0264	**	0.0183	**	0.6863	-	0.7104	-	0.2029	-	0.2408	-
Female	0.8250	-	0.9413	-	0.8870	-	0.9088	-	0.1141	-	0.1008	-	0.6857	-	0.7627	-
Education: High school or equivalent	0.7799	-	0.9347	-	0.0114	**	0.0020	***	0.0019	***	0.0013	***	0.0008	***	0.0000	***
Education: Undergraduate or more	0.5051	-	0.7368	-	0.0000	***	0.0000	***	0.0000	***	0.0000	***	0.0000	***	0.0000	***
Religion: Catholic	0.8468	-	0.9413	-	0.9318	-	0.9422	-	0.0512	*	0.0865	*	0.7322	-	0.7808	-
Religion: Evangelical Pentecostal or other evangelical	0.2085	-	0.2717	-	0.0598	*	0.0055	***	0.0030	***	0.0041	***	0.0468	**	0.0035	***
Religion: Evangelical Traditional	0.3355	-	0.5472	-	0.1392	-	0.3756	-	0.0610	*	0.0848	*	0.5510	-	0.7153	-
Religion: Others/No Relig.	0.9506	-	0.9746	-	0.5189	-	0.8378	-	0.0967	*	0.1493	-	0.3917	-	0.5604	-
Income: 1 - 2 minimum wages	0.0091	***	0.0265	**	0.5799	-	0.7054	-	0.0337	**	0.0326	**	0.1041	-	0.0760	*
Income: 2 - 3 minimum wages	0.9757	-	0.9746	-	0.4032	-	0.5563	-	0.0088	***	0.0138	**	0.1717	-	0.2217	-
Income: 3 - 5 minimum wages	0.5492	-	0.7368	-	0.8558	-	0.9088	-	0.0318	**	0.0439	**	0.5797	-	0.6918	-
Income: 5 - 10 minimum wages	0.8961	-	0.9549	-	0.8690	-	0.9088	-	0.1030	-	0.0848	*	0.7328	-	0.7627	-
Income: 10 minimum wages or more	0.5084	-	0.7368	-	0.7482	-	0.9088	-	0.0168	**	0.0335	**	0.2668	-	0.3398	-
Income: Do not know/ Prefer to not answer	0.6496	-	0.7904	-	0.7001	-	0.8720	-	0.8005	-	0.7684	-	0.8354	-	0.8300	-
Age (Years)	0.1205	-	0.2498	-	0.4087	-	0.3072	-	0.0816	*	0.0439	**	0.9046	-	0.8383	-
Race: Black	0.0255	**	0.0108	**	0.8553	-	0.9088	-	0.2719	-	0.2982	-	0.7080	-	0.7627	-

Notes: This table presents the standard and adjusted (Benjamini-Hochberg) p-values for each of the four main OLS models estimated for the whole sample. Signif. Codes: ***: 0.01, **: 0.05, *: 0.1.p-values from standard and adjusted (Benjamini-Hochberg) two-sided t-tests for the null hypothesis of a zero average parameter.

3.3 Linear Combination of Coefficients

Table 23: Testing differences between Scientific Consensus and other psychological predictors - Belief in the existence of climate change

Variable	Res.Df	Df	F	P-value
Subjective Knowledge	2861	1	18.86	< 0.001
Objective Knowledge	2861	1	16.94	< 0.001
The New Ecological Paradigm (NEP)	2861	1	5.47	0.02
Egalitarianism worldview	2861	1	32.84	< 0.001
Personal experience (extreme weather events)	2861	1	9.01	< 0.001

Notes: This table reports the results of an one-sided F-test to evaluate if the coefficient of Scientific Consensus and is statistically different from the coefficient of each of the other statistically significant psychological predictors of the belief in the existence of climate change. Each row represents the result of testing the difference between the coefficient of Scientific Consensus and that of the respective variable.

Table 24: Testing differences between Individualism worldview and other psychological predictors - Belief in anthropogenic climate change

Variable	Res.Df	Df	F	P-value
Objective Knowledge	2857	1	1.34	0.25
Scientific Consensus	2857	1	2.35	0.13
Trust in Scientists	2857	1	0.40	0.53
The New Ecological Paradigm (NEP)	2857	1	0.19	0.66

Notes: This table reports the results of an one-sided F-test to evaluate if the coefficient of Individualism worldview and is statistically different from the coefficient of each of the other statistically significant psychological predictors of the belief in anthropogenic climate change. Each row represents the result of testing the difference between the coefficient of Individual worldview and that of the respective variable.

Table 25: Testing differences between Individualism worldview and other psychological predictors - Belief in the consequences of climate change

Variable	Res.Df	Df	F	P-value
Objective Knowledge	2861	1	5.09	0.02
Scientific Consensus	2861	1	2.78	0.10
The New Ecological Paradigm (NEP)	2861	1	1.96	0.16

Notes: This table reports the results of an one-sided F-test to evaluate if the coefficient of Individualism worldview and is statistically different from the coefficient of each of the other statistically significant psychological predictors of the belief in the consequences of climate change. Each row represents the result of testing the difference between the coefficient of Individual worldview and that of the respective variable.

Table 26: Testing differences between Insignificant and Significant predictors - Belief in the existence of climate change (after independent variables standardization)

Insignif. Variable	Signif. Variable	Res.Df	Df	F	Pr(>F)	Check
Main Correlates						
Political ideology: Left	Scientific consensus	2861	1	3.3e+01	0.00	***
Trust in scientists	Scientific consensus	2861	1	1.9e+01	0.00	***
Individualism worldview	Scientific consensus	2861	1	4.6e+01	0.00	***
Female	Scientific consensus	2861	1	4.0e+01	0.00	***
Education: High school or equivalent	Scientific consensus	2861	1	3.9e+01	0.00	***
Education: Undergraduate or more	Scientific consensus	2861	1	3.1e+01	0.00	***
Religion: Catholic	Scientific consensus	2861	1	1.9e+01	0.00	***
Religion: Evangelical Pentecostal or other evangelical	Scientific consensus	2861	1	2.1e+01	0.00	***
Religion: Evangelical Traditional	Scientific consensus	2861	1	2.2e+01	0.00	***
Religion: Others/No Relig.	Scientific consensus	2861	1	2.1e+01	0.00	***
Income: 2 - 3 minimum wages	Scientific consensus	2861	1	3.7e+01	0.00	***
Income: 3 - 5 minimum wages	Scientific consensus	2861	1	4.0e+01	0.00	***
Income: 5 - 10 minimum wages	Scientific consensus	2861	1	3.5e+01	0.00	***
Income: 10 minimum wages or more	Scientific consensus	2861	1	3.2e+01	0.00	***
Income: Do not know/ Prefer to not answer	Scientific consensus	2861	1	2.9e+01	0.00	***
Age (Years)	Scientific consensus	2861	1	5.2e+01	0.00	***
Political ideology: Left	The New Ecological Paradigm (NEP)	2861	1	2.9e+01	0.00	***
Trust in scientists	The New Ecological Paradigm (NEP)	2861	1	1.0e+01	0.00	***
Individualism worldview	The New Ecological Paradigm (NEP)	2861	1	2.9e+01	0.00	***
Female	The New Ecological Paradigm (NEP)	2861	1	3.2e+01	0.00	***
Education: High school or equivalent	The New Ecological Paradigm (NEP)	2861	1	2.0e+01	0.00	***
Education: Undergraduate or more	The New Ecological Paradigm (NEP)	2861	1	1.6e+01	0.00	***
Religion: Catholic	The New Ecological Paradigm (NEP)	2861	1	1.3e+01	0.00	***
Religion: Evangelical Pentecostal or other evangelical	The New Ecological Paradigm (NEP)	2861	1	1.4e+01	0.00	***
Religion: Evangelical Traditional	The New Ecological Paradigm (NEP)	2861	1	1.5e+01	0.00	***

Religion: Others/No Relig.	The New Ecological Paradigm (NEP)	2861	1	1.5e+01	0.00	***
Income: 2 - 3 minimum wages	The New Ecological Paradigm (NEP)	2861	1	2.7e+01	0.00	***
Income: 3 - 5 minimum wages	The New Ecological Paradigm (NEP)	2861	1	3.0e+01	0.00	***
Income: 5 - 10 minimum wages	The New Ecological Paradigm (NEP)	2861	1	2.9e+01	0.00	***
Income: 10 minimum wages or more	The New Ecological Paradigm (NEP)	2861	1	2.5e+01	0.00	***
Income: Do not know/ Prefer to not answer	The New Ecological Paradigm (NEP)	2861	1	1.9e+01	0.00	***
Age (Years)	The New Ecological Paradigm (NEP)	2861	1	4.2e+01	0.00	***
Political ideology: Left	Personal experience (extreme weather events)	2861	1	7.7e+00	0.01	***
Trust in scientists	Personal experience (extreme weather events)	2861	1	2.1e+00	0.14	
Individualism worldview	Personal experience (extreme weather events)	2861	1	1.3e+01	0.00	***
Female	Personal experience (extreme weather events)	2861	1	9.6e+00	0.00	***
Education: High school or equivalent	Personal experience (extreme weather events)	2861	1	7.0e+00	0.01	***
Education: Undergraduate or more	Personal experience (extreme weather events)	2861	1	4.8e+00	0.03	**
Religion: Catholic	Personal experience (extreme weather events)	2861	1	3.8e+00	0.05	*
Religion: Evangelical Pentecostal or other evangelical	Personal experience (extreme weather events)	2861	1	3.0e+00	0.08	*
Religion: Evangelical Traditional	Personal experience (extreme weather events)	2861	1	3.4e+00	0.06	*
Religion: Others/No Relig.	Personal experience (extreme weather events)	2861	1	4.4e+00	0.04	**
Income: 2 - 3 minimum wages	Personal experience (extreme weather events)	2861	1	9.3e+00	0.00	***
Income: 3 - 5 minimum wages	Personal experience (extreme weather events)	2861	1	1.2e+01	0.00	***
Income: 5 - 10 minimum wages	Personal experience (extreme weather events)	2861	1	8.9e+00	0.00	***
Income: 10 minimum wages or more	Personal experience (extreme weather events)	2861	1	6.9e+00	0.01	***
Income: Do not know/ Prefer to not answer	Personal experience (extreme weather events)	2861	1	5.6e+00	0.02	**
Age (Years)	Personal experience (extreme weather events)	2861	1	1.5e+01	0.00	***
Political ideology: Left	Race: Black	2861	1	4.2e+00	0.04	**
Trust in scientists	Race: Black	2861	1	6.8e+00	0.01	***
Individualism worldview	Race: Black	2861	1	1.6e+00	0.20	
Female	Race: Black	2861	1	3.9e+00	0.05	**
Education: High school or equivalent	Race: Black	2861	1	4.0e+00	0.05	**
Education: Undergraduate or more	Race: Black	2861	1	4.8e+00	0.03	**
Religion: Catholic	Race: Black	2861	1	1.6e+00	0.21	

Religion: Evangelical Pentecostal or other evangelical	Race: Black	2861	1	5.0e+00	0.03	**
Religion: Evangelical Traditional	Race: Black	2861	1	4.7e+00	0.03	**
Religion: Others/No Relig.	Race: Black	2861	1	1.5e+00	0.22	
Income: 2 - 3 minimum wages	Race: Black	2861	1	2.8e+00	0.10	*
Income: 3 - 5 minimum wages	Race: Black	2861	1	1.6e+00	0.21	
Income: 5 - 10 minimum wages	Race: Black	2861	1	3.1e+00	0.08	*
Income: 10 minimum wages or more	Race: Black	2861	1	4.6e+00	0.03	**
Income: Do not know/ Prefer to not answer	Race: Black	2861	1	3.5e+00	0.06	*
Age (Years)	Race: Black	2861	1	5.4e+00	0.02	**
Political ideology: Left	Political ideology: Progressive	2861	1	2.6e+00	0.11	
Trust in scientists	Political ideology: Progressive	2861	1	4.0e-02	0.83	
Individualism worldview	Political ideology: Progressive	2861	1	7.3e+00	0.01	***
Female	Political ideology: Progressive	2861	1	3.8e+00	0.05	*
Education: High school or equivalent	Political ideology: Progressive	2861	1	2.3e+00	0.13	
Education: Undergraduate or more	Political ideology: Progressive	2861	1	1.3e+00	0.25	
Religion: Catholic	Political ideology: Progressive	2861	1	1.2e+00	0.28	
Religion: Evangelical Pentecostal or other evangelical	Political ideology: Progressive	2861	1	3.2e-01	0.57	
Religion: Evangelical Traditional	Political ideology: Progressive	2861	1	6.1e-01	0.43	
Religion: Others/No Relig.	Political ideology: Progressive	2861	1	1.6e+00	0.21	
Income: 2 - 3 minimum wages	Political ideology: Progressive	2861	1	4.3e+00	0.04	**
Income: 3 - 5 minimum wages	Political ideology: Progressive	2861	1	7.1e+00	0.01	***
Income: 5 - 10 minimum wages	Political ideology: Progressive	2861	1	3.8e+00	0.05	*
Income: 10 minimum wages or more	Political ideology: Progressive	2861	1	2.8e+00	0.10	*
Income: Do not know/ Prefer to not answer	Political ideology: Progressive	2861	1	1.8e+00	0.18	
Age (Years)	Political ideology: Progressive	2861	1	9.5e+00	0.00	***
Political ideology: Left	Subjective knowledge	2861	1	9.4e+00	0.00	***
Trust in scientists	Subjective knowledge	2861	1	1.9e+00	0.17	
Individualism worldview	Subjective knowledge	2861	1	1.3e+01	0.00	***
Female	Subjective knowledge	2861	1	9.3e+00	0.00	***
Education: High school or equivalent	Subjective knowledge	2861	1	5.8e+00	0.02	**

Education: Undergraduate or more	Subjective knowledge	2861	1	4.3e+00	0.04	**
Religion: Catholic	Subjective knowledge	2861	1	3.3e+00	0.07	*
Religion: Evangelical Pentecostal or other evangelical	Subjective knowledge	2861	1	2.5e+00	0.11	
Religion: Evangelical Traditional	Subjective knowledge	2861	1	2.7e+00	0.10	*
Religion: Others/No Relig.	Subjective knowledge	2861	1	4.3e+00	0.04	**
Income: 2 - 3 minimum wages	Subjective knowledge	2861	1	9.2e+00	0.00	***
Income: 3 - 5 minimum wages	Subjective knowledge	2861	1	1.1e+01	0.00	***
Income: 5 - 10 minimum wages	Subjective knowledge	2861	1	1.0e+01	0.00	***
Income: 10 minimum wages or more	Subjective knowledge	2861	1	6.7e+00	0.01	***
Income: Do not know/ Prefer to not answer	Subjective knowledge	2861	1	5.5e+00	0.02	**
Age (Years)	Subjective knowledge	2861	1	1.8e+01	0.00	***
Political ideology: Left	Objective knowledge	2861	1	1.1e+01	0.00	***
Trust in scientists	Objective knowledge	2861	1	6.0e+00	0.01	**
Individualism worldview	Objective knowledge	2861	1	1.7e+01	0.00	***
Female	Objective knowledge	2861	1	1.4e+01	0.00	***
Education: High school or equivalent	Objective knowledge	2861	1	1.0e+01	0.00	***
Education: Undergraduate or more	Objective knowledge	2861	1	8.6e+00	0.00	***
Religion: Catholic	Objective knowledge	2861	1	6.6e+00	0.01	**
Religion: Evangelical Pentecostal or other evangelical	Objective knowledge	2861	1	6.2e+00	0.01	**
Religion: Evangelical Traditional	Objective knowledge	2861	1	6.5e+00	0.01	**
Religion: Others/No Relig.	Objective knowledge	2861	1	8.2e+00	0.00	***
Income: 2 - 3 minimum wages	Objective knowledge	2861	1	1.4e+01	0.00	***
Income: 3 - 5 minimum wages	Objective knowledge	2861	1	1.5e+01	0.00	***
Income: 5 - 10 minimum wages	Objective knowledge	2861	1	1.4e+01	0.00	***
Income: 10 minimum wages or more	Objective knowledge	2861	1	1.1e+01	0.00	***
Income: Do not know/ Prefer to not answer	Objective knowledge	2861	1	1.1e+01	0.00	***
Age (Years)	Objective knowledge	2861	1	1.8e+01	0.00	***
Political ideology: Left	Egalitarianism worldview	2861	1	1.4e+00	0.24	
Trust in scientists	Egalitarianism worldview	2861	1	1.0e-02	0.93	
Individualism worldview	Egalitarianism worldview	2861	1	4.4e+00	0.04	**

Female	Egalitarianism worldview	2861	1	2.6e+00	0.11	
Education: High school or equivalent	Egalitarianism worldview	2861	1	2.0e+00	0.16	
Education: Undergraduate or more	Egalitarianism worldview	2861	1	1.2e+00	0.28	
Religion: Catholic	Egalitarianism worldview	2861	1	7.9e-01	0.37	
Religion: Evangelical Pentecostal or other evangelical	Egalitarianism worldview	2861	1	1.2e-01	0.72	
Religion: Evangelical Traditional	Egalitarianism worldview	2861	1	3.1e-01	0.58	
Religion: Others/No Relig.	Egalitarianism worldview	2861	1	1.2e+00	0.27	
Income: 2 - 3 minimum wages	Egalitarianism worldview	2861	1	2.8e+00	0.10	*
Income: 3 - 5 minimum wages	Egalitarianism worldview	2861	1	4.4e+00	0.04	**
Income: 5 - 10 minimum wages	Egalitarianism worldview	2861	1	2.5e+00	0.12	
Income: 10 minimum wages or more	Egalitarianism worldview	2861	1	1.7e+00	0.19	
Income: Do not know/ Prefer to not answer	Egalitarianism worldview	2861	1	1.4e+00	0.24	
Age (Years)	Egalitarianism worldview	2861	1	4.4e+00	0.04	**
Political ideology: Left	Income: 1 - 2 minimum wages	2861	1	2.3e+00	0.13	
Trust in scientists	Income: 1 - 2 minimum wages	2861	1	6.0e-02	0.81	
Individualism worldview	Income: 1 - 2 minimum wages	2861	1	5.8e+00	0.02	**
Female	Income: 1 - 2 minimum wages	2861	1	3.5e+00	0.06	*
Education: High school or equivalent	Income: 1 - 2 minimum wages	2861	1	2.4e+00	0.12	
Education: Undergraduate or more	Income: 1 - 2 minimum wages	2861	1	1.3e+00	0.26	
Religion: Catholic	Income: 1 - 2 minimum wages	2861	1	9.4e-01	0.33	
Religion: Evangelical Pentecostal or other evangelical	Income: 1 - 2 minimum wages	2861	1	2.5e-01	0.62	
Religion: Evangelical Traditional	Income: 1 - 2 minimum wages	2861	1	4.7e-01	0.49	
Religion: Others/No Relig.	Income: 1 - 2 minimum wages	2861	1	1.4e+00	0.24	
Income: 2 - 3 minimum wages	Income: 1 - 2 minimum wages	2861	1	7.1e+00	0.01	***
Income: 3 - 5 minimum wages	Income: 1 - 2 minimum wages	2861	1	1.1e+01	0.00	***
Income: 5 - 10 minimum wages	Income: 1 - 2 minimum wages	2861	1	5.8e+00	0.02	**
Income: 10 minimum wages or more	Income: 1 - 2 minimum wages	2861	1	4.2e+00	0.04	**
Income: Do not know/ Prefer to not answer	Income: 1 - 2 minimum wages	2861	1	3.1e+00	0.08	*
Age (Years)	Income: 1 - 2 minimum wages	2861	1	6.3e+00	0.01	**

Notes: This table reports the results of an one-sided F-test to evaluate if the insignificant coefficients (at the 95%) are statistically different from the significant coefficients (at the 95%) in the belief of climate change model. Each row represents the result of testing the difference between the coefficients. Before estimating the coefficients of the OLS model and using them to perform the one-sided F-test we standardized (i.e. scaled) all variables used on the right-hand side of our equation to allow for comparison. Signif. Codes: ***: 0.01, **: 0.05, *: 0.1.

Table 27: Testing differences between Insignificant and Significant predictors - Belief in anthropogenic climate change (after independent variables standardization)

Insignif. Variable	Signif. Variable	Res.Df	Df	F	Pr(>F)	Check
Main Correlates						
Political ideology: Left	Individualism worldview	2857	1	22.52	0.00	***
Subjective knowledge	Individualism worldview	2857	1	13.89	0.00	***
Egalitarianism worldview	Individualism worldview	2857	1	15.77	0.00	***
Personal experience (extreme weather events)	Individualism worldview	2857	1	16.04	0.00	***
Female	Individualism worldview	2857	1	16.91	0.00	***
Religion: Catholic	Individualism worldview	2857	1	6.16	0.01	**
Religion: Evangelical Pentecostal or other evangelical	Individualism worldview	2857	1	0.34	0.56	
Religion: Evangelical Traditional	Individualism worldview	2857	1	16.66	0.00	***
Religion: Others/No Relig.	Individualism worldview	2857	1	4.78	0.03	**
Income: 1 - 2 minimum wages	Individualism worldview	2857	1	10.96	0.00	***
Income: 2 - 3 minimum wages	Individualism worldview	2857	1	16.86	0.00	***
Income: 3 - 5 minimum wages	Individualism worldview	2857	1	9.89	0.00	***
Income: 5 - 10 minimum wages	Individualism worldview	2857	1	12.03	0.00	***
Income: 10 minimum wages or more	Individualism worldview	2857	1	13.59	0.00	***
Income: Do not know/ Prefer to not answer	Individualism worldview	2857	1	10.70	0.00	***
Race: Black	Individualism worldview	2857	1	9.66	0.00	***
Age (Years)	Individualism worldview	2857	1	24.10	0.00	***
Political ideology: Left	Scientific consensus	2857	1	4.85	0.03	**
Subjective knowledge	Scientific consensus	2857	1	7.53	0.01	***
Egalitarianism worldview	Scientific consensus	2857	1	5.32	0.02	**
Personal experience (extreme weather events)	Scientific consensus	2857	1	5.66	0.02	**
Female	Scientific consensus	2857	1	8.86	0.00	***
Religion: Catholic	Scientific consensus	2857	1	6.65	0.01	***
Religion: Evangelical Pentecostal or other evangelical	Scientific consensus	2857	1	16.36	0.00	***
Religion: Evangelical Traditional	Scientific consensus	2857	1	3.73	0.05	*

Religion: Others/No Relig.	Scientific consensus	2857	1	8.86	0.00	***
Income: 1 - 2 minimum wages	Scientific consensus	2857	1	5.48	0.02	**
Income: 2 - 3 minimum wages	Scientific consensus	2857	1	6.08	0.01	**
Income: 3 - 5 minimum wages	Scientific consensus	2857	1	9.26	0.00	***
Income: 5 - 10 minimum wages	Scientific consensus	2857	1	8.56	0.00	***
Income: 10 minimum wages or more	Scientific consensus	2857	1	7.82	0.01	***
Income: Do not know/ Prefer to not answer	Scientific consensus	2857	1	5.59	0.02	**
Race: Black	Scientific consensus	2857	1	8.98	0.00	***
Age (Years)	Scientific consensus	2857	1	13.83	0.00	***
Political ideology: Left	Trust in scientists	2857	1	0.93	0.33	
Subjective knowledge	Trust in scientists	2857	1	3.00	0.08	*
Egalitarianism worldview	Trust in scientists	2857	1	1.43	0.23	
Personal experience (extreme weather events)	Trust in scientists	2857	1	1.35	0.25	
Female	Trust in scientists	2857	1	2.45	0.12	
Religion: Catholic	Trust in scientists	2857	1	2.59	0.11	
Religion: Evangelical Pentecostal or other evangelical	Trust in scientists	2857	1	4.94	0.03	**
Religion: Evangelical Traditional	Trust in scientists	2857	1	0.84	0.36	
Religion: Others/No Relig.	Trust in scientists	2857	1	4.06	0.04	**
Income: 1 - 2 minimum wages	Trust in scientists	2857	1	2.17	0.14	
Income: 2 - 3 minimum wages	Trust in scientists	2857	1	2.13	0.14	
Income: 3 - 5 minimum wages	Trust in scientists	2857	1	3.90	0.05	**
Income: 5 - 10 minimum wages	Trust in scientists	2857	1	4.12	0.04	**
Income: 10 minimum wages or more	Trust in scientists	2857	1	3.22	0.07	*
Income: Do not know/ Prefer to not answer	Trust in scientists	2857	1	2.22	0.14	
Race: Black	Trust in scientists	2857	1	3.35	0.07	*
Age (Years)	Trust in scientists	2857	1	3.83	0.05	*
Other Correlates						
Political ideology: Left	Political ideology: Progressive	2857	1	6.30	0.01	**
Subjective knowledge	Political ideology: Progressive	2857	1	2.69	0.10	

Egalitarianism worldview	Political ideology: Progressive	2857	1	5.57	0.02	**
Personal experience (extreme weather events)	Political ideology: Progressive	2857	1	6.07	0.01	**
Female	Political ideology: Progressive	2857	1	2.88	0.09	*
Religion: Catholic	Political ideology: Progressive	2857	1	1.43	0.23	
Religion: Evangelical Pentecostal or other evangelical	Political ideology: Progressive	2857	1	0.64	0.42	
Religion: Evangelical Traditional	Political ideology: Progressive	2857	1	6.70	0.01	***
Religion: Others/No Relig.	Political ideology: Progressive	2857	1	0.57	0.45	
Income: 1 - 2 minimum wages	Political ideology: Progressive	2857	1	2.33	0.13	
Income: 2 - 3 minimum wages	Political ideology: Progressive	2857	1	3.46	0.06	*
Income: 3 - 5 minimum wages	Political ideology: Progressive	2857	1	1.35	0.24	
Income: 5 - 10 minimum wages	Political ideology: Progressive	2857	1	1.68	0.20	
Income: 10 minimum wages or more	Political ideology: Progressive	2857	1	2.45	0.12	
Income: Do not know/ Prefer to not answer	Political ideology: Progressive	2857	1	2.07	0.15	
Race: Black	Political ideology: Progressive	2857	1	1.86	0.17	
Age (Years)	Political ideology: Progressive	2857	1	4.94	0.03	**
Political ideology: Left	Objective knowledge	2857	1	11.41	0.00	***
Subjective knowledge	Objective knowledge	2857	1	15.41	0.00	***
Egalitarianism worldview	Objective knowledge	2857	1	10.63	0.00	***
Personal experience (extreme weather events)	Objective knowledge	2857	1	12.01	0.00	***
Female	Objective knowledge	2857	1	16.89	0.00	***
Religion: Catholic	Objective knowledge	2857	1	13.43	0.00	***
Religion: Evangelical Pentecostal or other evangelical	Objective knowledge	2857	1	21.73	0.00	***
Religion: Evangelical Traditional	Objective knowledge	2857	1	9.80	0.00	***
Religion: Others/No Relig.	Objective knowledge	2857	1	15.81	0.00	***
Income: 1 - 2 minimum wages	Objective knowledge	2857	1	11.65	0.00	***
Income: 2 - 3 minimum wages	Objective knowledge	2857	1	13.59	0.00	***
Income: 3 - 5 minimum wages	Objective knowledge	2857	1	16.80	0.00	***
Income: 5 - 10 minimum wages	Objective knowledge	2857	1	15.96	0.00	***
Income: 10 minimum wages or more	Objective knowledge	2857	1	15.14	0.00	***
Income: Do not know/ Prefer to not answer	Objective knowledge	2857	1	11.83	0.00	***

Race: Black	Objective knowledge	2857	1	18.31	0.00	***
Age (Years)	Objective knowledge	2857	1	22.52	0.00	***
Political ideology: Left	The New Ecological Paradigm (NEP)	2857	1	3.72	0.05	*
Subjective knowledge	The New Ecological Paradigm (NEP)	2857	1	5.00	0.03	**
Egalitarianism worldview	The New Ecological Paradigm (NEP)	2857	1	2.89	0.09	*
Personal experience (extreme weather events)	The New Ecological Paradigm (NEP)	2857	1	3.56	0.06	*
Female	The New Ecological Paradigm (NEP)	2857	1	7.17	0.01	***
Religion: Catholic	The New Ecological Paradigm (NEP)	2857	1	4.57	0.03	**
Religion: Evangelical Pentecostal or other evangelical	The New Ecological Paradigm (NEP)	2857	1	14.26	0.00	***
Religion: Evangelical Traditional	The New Ecological Paradigm (NEP)	2857	1	2.40	0.12	
Religion: Others/No Relig.	The New Ecological Paradigm (NEP)	2857	1	6.38	0.01	**
Income: 1 - 2 minimum wages	The New Ecological Paradigm (NEP)	2857	1	3.06	0.08	*
Income: 2 - 3 minimum wages	The New Ecological Paradigm (NEP)	2857	1	3.83	0.05	*
Income: 3 - 5 minimum wages	The New Ecological Paradigm (NEP)	2857	1	5.77	0.02	**
Income: 5 - 10 minimum wages	The New Ecological Paradigm (NEP)	2857	1	6.30	0.01	**
Income: 10 minimum wages or more	The New Ecological Paradigm (NEP)	2857	1	5.26	0.02	**
Income: Do not know/ Prefer to not answer	The New Ecological Paradigm (NEP)	2857	1	3.78	0.05	*
Race: Black	The New Ecological Paradigm (NEP)	2857	1	6.50	0.01	**
Age (Years)	The New Ecological Paradigm (NEP)	2857	1	9.91	0.00	***
Political ideology: Left	Education: High school or equivalent	2857	1	1.47	0.23	
Subjective knowledge	Education: High school or equivalent	2857	1	3.34	0.07	*
Egalitarianism worldview	Education: High school or equivalent	2857	1	2.13	0.14	
Personal experience (extreme weather events)	Education: High school or equivalent	2857	1	2.51	0.11	
Female	Education: High school or equivalent	2857	1	3.43	0.06	*
Religion: Catholic	Education: High school or equivalent	2857	1	3.88	0.05	**
Religion: Evangelical Pentecostal or other evangelical	Education: High school or equivalent	2857	1	7.64	0.01	***
Religion: Evangelical Traditional	Education: High school or equivalent	2857	1	1.30	0.25	
Religion: Others/No Relig.	Education: High school or equivalent	2857	1	5.17	0.02	**
Income: 1 - 2 minimum wages	Education: High school or equivalent	2857	1	2.64	0.10	
Income: 2 - 3 minimum wages	Education: High school or equivalent	2857	1	2.66	0.10	

Income: 3 - 5 minimum wages	Education: High school or equivalent	2857	1	5.03	0.02	**
Income: 5 - 10 minimum wages	Education: High school or equivalent	2857	1	4.66	0.03	**
Income: 10 minimum wages or more	Education: High school or equivalent	2857	1	4.41	0.04	**
Income: Do not know/ Prefer to not answer	Education: High school or equivalent	2857	1	2.93	0.09	*
Race: Black	Education: High school or equivalent	2857	1	5.27	0.02	**
Age (Years)	Education: High school or equivalent	2857	1	6.50	0.01	**
Political ideology: Left	Education: Undergraduate or more	2857	1	6.55	0.01	**
Subjective knowledge	Education: Undergraduate or more	2857	1	8.27	0.00	***
Egalitarianism worldview	Education: Undergraduate or more	2857	1	8.02	0.00	***
Personal experience (extreme weather events)	Education: Undergraduate or more	2857	1	7.01	0.01	***
Female	Education: Undergraduate or more	2857	1	10.65	0.00	***
Religion: Catholic	Education: Undergraduate or more	2857	1	10.00	0.00	***
Religion: Evangelical Pentecostal or other evangelical	Education: Undergraduate or more	2857	1	13.97	0.00	***
Religion: Evangelical Traditional	Education: Undergraduate or more	2857	1	6.18	0.01	**
Religion: Others/No Relig.	Education: Undergraduate or more	2857	1	11.14	0.00	***
Income: 1 - 2 minimum wages	Education: Undergraduate or more	2857	1	6.98	0.01	***
Income: 2 - 3 minimum wages	Education: Undergraduate or more	2857	1	7.43	0.01	***
Income: 3 - 5 minimum wages	Education: Undergraduate or more	2857	1	10.88	0.00	***
Income: 5 - 10 minimum wages	Education: Undergraduate or more	2857	1	9.77	0.00	***
Income: 10 minimum wages or more	Education: Undergraduate or more	2857	1	10.74	0.00	***
Income: Do not know/ Prefer to not answer	Education: Undergraduate or more	2857	1	7.73	0.01	***
Race: Black	Education: Undergraduate or more	2857	1	13.78	0.00	***
Age (Years)	Education: Undergraduate or more	2857	1	16.92	0.00	***

Notes: This table reports the results of an one-sided F-test to evaluate if the insignificant coefficients (at the 95%) are statistically different from the significant coefficients (at the 95%) in the anthropogenic climate change model. Each row represents the result of testing the difference between the coefficients. Before estimating the coefficients of the OLS model and using them to perform the one-sided F-test we standardized (i.e. scaled) all variables used on the right-hand side of our equation to allow for comparison. Signif. Codes: ***: 0.01, **: 0.05, *: 0.1.

Table 28: Testing differences between Insignificant and Significant predictors - Belief in consequences of climate change (after independent variables standardization)

Insignif. Variable	Signif. Variable	Res.Df	Df	F	Pr(>F)	Check
Main Correlates						
Political ideology: Progressive	Individualism worldview	2861	1	36.09	0.00	***
Subjective knowledge	Individualism worldview	2861	1	40.08	0.00	***
Trust in scientists	Individualism worldview	2861	1	34.14	0.00	***
Egalitarianism worldview	Individualism worldview	2861	1	33.43	0.00	***
Personal experience (extreme weather events)	Individualism worldview	2861	1	46.96	0.00	***
Female	Individualism worldview	2861	1	21.48	0.00	***
Religion: Catholic	Individualism worldview	2861	1	4.62	0.03	**
Religion: Evangelical Traditional	Individualism worldview	2861	1	9.85	0.00	***
Religion: Others/No Relig.	Individualism worldview	2861	1	7.37	0.01	***
Income: 5 - 10 minimum wages	Individualism worldview	2861	1	62.39	0.00	***
Income: Do not know/ Prefer to not answer	Individualism worldview	2861	1	27.85	0.00	***
Race: Black	Individualism worldview	2861	1	45.30	0.00	***
Age (Years)	Individualism worldview	2861	1	65.54	0.00	***
Political ideology: Progressive	Objective knowledge	2861	1	11.14	0.00	***
Subjective knowledge	Objective knowledge	2861	1	8.55	0.00	***
Trust in scientists	Objective knowledge	2861	1	4.94	0.03	**
Egalitarianism worldview	Objective knowledge	2861	1	9.44	0.00	***
Personal experience (extreme weather events)	Objective knowledge	2861	1	5.08	0.02	**
Female	Objective knowledge	2861	1	20.66	0.00	***
Religion: Catholic	Objective knowledge	2861	1	17.38	0.00	***
Religion: Evangelical Traditional	Objective knowledge	2861	1	19.61	0.00	***
Religion: Others/No Relig.	Objective knowledge	2861	1	16.41	0.00	***
Income: 5 - 10 minimum wages	Objective knowledge	2861	1	6.32	0.01	**
Income: Do not know/ Prefer to not answer	Objective knowledge	2861	1	8.56	0.00	***
Race: Black	Objective knowledge	2861	1	9.87	0.00	***

Age (Years)	Objective knowledge	2861	1	20.05	0.00	***
Political ideology: Progressive	The New Ecological Paradigm (NEP)	2861	1	8.47	0.00	***
Subjective knowledge	The New Ecological Paradigm (NEP)	2861	1	6.34	0.01	**
Trust in scientists	The New Ecological Paradigm (NEP)	2861	1	3.15	0.08	*
Egalitarianism worldview	The New Ecological Paradigm (NEP)	2861	1	6.51	0.01	**
Personal experience (extreme weather events)	The New Ecological Paradigm (NEP)	2861	1	3.00	0.08	*
Female	The New Ecological Paradigm (NEP)	2861	1	17.92	0.00	***
Religion: Catholic	The New Ecological Paradigm (NEP)	2861	1	15.97	0.00	***
Religion: Evangelical Traditional	The New Ecological Paradigm (NEP)	2861	1	16.98	0.00	***
Religion: Others/No Relig.	The New Ecological Paradigm (NEP)	2861	1	14.24	0.00	***
Income: 5 - 10 minimum wages	The New Ecological Paradigm (NEP)	2861	1	4.22	0.04	**
Income: Do not know/ Prefer to not answer	The New Ecological Paradigm (NEP)	2861	1	5.92	0.02	**
Race: Black	The New Ecological Paradigm (NEP)	2861	1	6.77	0.01	***
Age (Years)	The New Ecological Paradigm (NEP)	2861	1	17.32	0.00	***
Political ideology: Progressive	Scientific consensus	2861	1	2.20	0.14	
Subjective knowledge	Scientific consensus	2861	1	1.21	0.27	
Trust in scientists	Scientific consensus	2861	1	0.45	0.50	
Egalitarianism worldview	Scientific consensus	2861	1	1.99	0.16	
Personal experience (extreme weather events)	Scientific consensus	2861	1	0.26	0.61	
Female	Scientific consensus	2861	1	7.58	0.01	***
Religion: Catholic	Scientific consensus	2861	1	8.08	0.00	***
Religion: Evangelical Traditional	Scientific consensus	2861	1	8.24	0.00	***
Religion: Others/No Relig.	Scientific consensus	2861	1	7.13	0.01	***
Income: 5 - 10 minimum wages	Scientific consensus	2861	1	0.46	0.50	
Income: Do not know/ Prefer to not answer	Scientific consensus	2861	1	1.79	0.18	
Race: Black	Scientific consensus	2861	1	1.31	0.25	
Age (Years)	Scientific consensus	2861	1	4.67	0.03	**
Political ideology: Progressive	Education: High school or equivalent	2861	1	4.80	0.03	**
Subjective knowledge	Education: High school or equivalent	2861	1	3.43	0.06	*
Trust in scientists	Education: High school or equivalent	2861	1	2.06	0.15	

Egalitarianism worldview	Education: High school or equivalent	2861	1	5.23	0.02	**
Personal experience (extreme weather events)	Education: High school or equivalent	2861	1	1.64	0.20	
Female	Education: High school or equivalent	2861	1	11.05	0.00	***
Religion: Catholic	Education: High school or equivalent	2861	1	11.70	0.00	***
Religion: Evangelical Traditional	Education: High school or equivalent	2861	1	12.47	0.00	***
Religion: Others/No Relig.	Education: High school or equivalent	2861	1	10.50	0.00	***
Income: 5 - 10 minimum wages	Education: High school or equivalent	2861	1	2.24	0.13	
Income: Do not know/ Prefer to not answer	Education: High school or equivalent	2861	1	4.20	0.04	**
Race: Black	Education: High school or equivalent	2861	1	4.06	0.04	**
Age (Years)	Education: High school or equivalent	2861	1	9.10	0.00	***
Political ideology: Progressive	Education: Undergraduate or more	2861	1	16.24	0.00	***
Subjective knowledge	Education: Undergraduate or more	2861	1	13.92	0.00	***
Trust in scientists	Education: Undergraduate or more	2861	1	9.83	0.00	***
Egalitarianism worldview	Education: Undergraduate or more	2861	1	19.95	0.00	***
Personal experience (extreme weather events)	Education: Undergraduate or more	2861	1	9.73	0.00	***
Female	Education: Undergraduate or more	2861	1	33.79	0.00	***
Religion: Catholic	Education: Undergraduate or more	2861	1	24.55	0.00	***
Religion: Evangelical Traditional	Education: Undergraduate or more	2861	1	29.27	0.00	***
Religion: Others/No Relig.	Education: Undergraduate or more	2861	1	22.97	0.00	***
Income: 5 - 10 minimum wages	Education: Undergraduate or more	2861	1	11.30	0.00	***
Income: Do not know/ Prefer to not answer	Education: Undergraduate or more	2861	1	13.32	0.00	***
Race: Black	Education: Undergraduate or more	2861	1	17.44	0.00	***
Age (Years)	Education: Undergraduate or more	2861	1	30.95	0.00	***
Political ideology: Progressive	Political ideology: Left	2861	1	7.63	0.01	***
Subjective knowledge	Political ideology: Left	2861	1	6.20	0.01	**
Trust in scientists	Political ideology: Left	2861	1	2.98	0.08	*
Egalitarianism worldview	Political ideology: Left	2861	1	5.77	0.02	**
Personal experience (extreme weather events)	Political ideology: Left	2861	1	2.69	0.10	
Female	Political ideology: Left	2861	1	18.95	0.00	***
Religion: Catholic	Political ideology: Left	2861	1	14.85	0.00	***

Religion: Evangelical Traditional	Political ideology: Left	2861	1	17.87	0.00	***
Religion: Others/No Relig.	Political ideology: Left	2861	1	12.85	0.00	***
Income: 5 - 10 minimum wages	Political ideology: Left	2861	1	3.42	0.06	*
Income: Do not know/ Prefer to not answer	Political ideology: Left	2861	1	5.60	0.02	**
Race: Black	Political ideology: Left	2861	1	6.22	0.01	**
Age (Years)	Political ideology: Left	2861	1	17.49	0.00	***
Other Correlates						
Political ideology: Progressive	Religion: Evangelical Pentecostal or other evangelical	2861	1	8.44	0.00	***
Subjective knowledge	Religion: Evangelical Pentecostal or other evangelical	2861	1	8.13	0.00	***
Trust in scientists	Religion: Evangelical Pentecostal or other evangelical	2861	1	7.88	0.01	***
Egalitarianism worldview	Religion: Evangelical Pentecostal or other evangelical	2861	1	6.03	0.01	**
Personal experience (extreme weather events)	Religion: Evangelical Pentecostal or other evangelical	2861	1	11.82	0.00	***
Female	Religion: Evangelical Pentecostal or other evangelical	2861	1	1.81	0.18	
Religion: Catholic	Religion: Evangelical Pentecostal or other evangelical	2861	1	0.07	0.80	
Religion: Evangelical Traditional	Religion: Evangelical Pentecostal or other evangelical	2861	1	0.85	0.36	
Religion: Others/No Relig.	Religion: Evangelical Pentecostal or other evangelical	2861	1	0.63	0.43	
Income: 5 - 10 minimum wages	Religion: Evangelical Pentecostal or other evangelical	2861	1	11.25	0.00	***
Income: Do not know/ Prefer to not answer	Religion: Evangelical Pentecostal or other evangelical	2861	1	5.05	0.02	**
Race: Black	Religion: Evangelical Pentecostal or other evangelical	2861	1	8.82	0.00	***
Age (Years)	Religion: Evangelical Pentecostal or other evangelical	2861	1	9.37	0.00	***
Political ideology: Progressive	Income: 1 - 2 minimum wages	2861	1	1.99	0.16	
Subjective knowledge	Income: 1 - 2 minimum wages	2861	1	1.18	0.28	
Trust in scientists	Income: 1 - 2 minimum wages	2861	1	0.45	0.50	
Egalitarianism worldview	Income: 1 - 2 minimum wages	2861	1	1.83	0.18	
Personal experience (extreme weather events)	Income: 1 - 2 minimum wages	2861	1	0.21	0.65	
Female	Income: 1 - 2 minimum wages	2861	1	7.94	0.00	***
Religion: Catholic	Income: 1 - 2 minimum wages	2861	1	7.87	0.01	***
Religion: Evangelical Traditional	Income: 1 - 2 minimum wages	2861	1	7.94	0.00	***
Religion: Others/No Relig.	Income: 1 - 2 minimum wages	2861	1	6.95	0.01	***

Income: 5 - 10 minimum wages	Income: 1 - 2 minimum wages	2861	1	0.58	0.45	
Income: Do not know/ Prefer to not answer	Income: 1 - 2 minimum wages	2861	1	2.87	0.09	*
Race: Black	Income: 1 - 2 minimum wages	2861	1	1.20	0.27	
Age (Years)	Income: 1 - 2 minimum wages	2861	1	4.04	0.04	**
Political ideology: Progressive	Income: 2 - 3 minimum wages	2861	1	2.63	0.10	
Subjective knowledge	Income: 2 - 3 minimum wages	2861	1	1.62	0.20	
Trust in scientists	Income: 2 - 3 minimum wages	2861	1	0.58	0.45	
Egalitarianism worldview	Income: 2 - 3 minimum wages	2861	1	2.38	0.12	
Personal experience (extreme weather events)	Income: 2 - 3 minimum wages	2861	1	0.31	0.58	
Female	Income: 2 - 3 minimum wages	2861	1	9.83	0.00	***
Religion: Catholic	Income: 2 - 3 minimum wages	2861	1	9.34	0.00	***
Religion: Evangelical Traditional	Income: 2 - 3 minimum wages	2861	1	9.66	0.00	***
Religion: Others/No Relig.	Income: 2 - 3 minimum wages	2861	1	8.04	0.00	***
Income: 5 - 10 minimum wages	Income: 2 - 3 minimum wages	2861	1	0.85	0.36	
Income: Do not know/ Prefer to not answer	Income: 2 - 3 minimum wages	2861	1	3.34	0.07	*
Race: Black	Income: 2 - 3 minimum wages	2861	1	1.70	0.19	
Age (Years)	Income: 2 - 3 minimum wages	2861	1	6.10	0.01	**
Political ideology: Progressive	Income: 3 - 5 minimum wages	2861	1	1.50	0.22	
Subjective knowledge	Income: 3 - 5 minimum wages	2861	1	0.73	0.39	
Trust in scientists	Income: 3 - 5 minimum wages	2861	1	0.16	0.68	
Egalitarianism worldview	Income: 3 - 5 minimum wages	2861	1	1.50	0.22	
Personal experience (extreme weather events)	Income: 3 - 5 minimum wages	2861	1	0.03	0.87	
Female	Income: 3 - 5 minimum wages	2861	1	7.54	0.01	***
Religion: Catholic	Income: 3 - 5 minimum wages	2861	1	7.80	0.01	***
Religion: Evangelical Traditional	Income: 3 - 5 minimum wages	2861	1	8.03	0.00	***
Religion: Others/No Relig.	Income: 3 - 5 minimum wages	2861	1	6.83	0.01	***
Income: 5 - 10 minimum wages	Income: 3 - 5 minimum wages	2861	1	0.17	0.68	
Income: Do not know/ Prefer to not answer	Income: 3 - 5 minimum wages	2861	1	1.95	0.16	
Race: Black	Income: 3 - 5 minimum wages	2861	1	0.77	0.38	
Age (Years)	Income: 3 - 5 minimum wages	2861	1	3.94	0.05	**

Political ideology: Progressive	Income: 10 minimum wages or more	2861	1	1.86	0.17	
Subjective knowledge	Income: 10 minimum wages or more	2861	1	0.94	0.33	
Trust in scientists	Income: 10 minimum wages or more	2861	1	0.24	0.62	
Egalitarianism worldview	Income: 10 minimum wages or more	2861	1	1.77	0.18	
Personal experience (extreme weather events)	Income: 10 minimum wages or more	2861	1	0.06	0.81	
Female	Income: 10 minimum wages or more	2861	1	8.59	0.00	***
Religion: Catholic	Income: 10 minimum wages or more	2861	1	8.35	0.00	***
Religion: Evangelical Traditional	Income: 10 minimum wages or more	2861	1	8.95	0.00	***
Religion: Others/No Relig.	Income: 10 minimum wages or more	2861	1	7.21	0.01	***
Income: 5 - 10 minimum wages	Income: 10 minimum wages or more	2861	1	0.28	0.60	
Income: Do not know/ Prefer to not answer	Income: 10 minimum wages or more	2861	1	2.20	0.14	
Race: Black	Income: 10 minimum wages or more	2861	1	0.99	0.32	
Age (Years)	Income: 10 minimum wages or more	2861	1	4.90	0.03	**

Notes: This table reports the results of an one-sided F-test to evaluate if the insignificant coefficients (at the 95%) are statistically different from the significant coefficients (at the 95%) in the consequences of climate change model. Each row represents the result of testing the difference between the coefficients. Before estimating the coefficients of the OLS model and using them to perform the one-sided F-test we standardized (i.e. scaled) all variables used on the right-hand side of our equation to allow for comparison. Signif. Codes: ***: 0.01, **: 0.05, *: 0.1.

3.4 Alternative Specifications

Table 29: Multilevel Model Results - Random Intercepts at the Country level

	Dependent Variable:			
	Existence (i)	Anthropogenic Causes (ii)	Consequences (iii)	Belief (Index) (iv)
Psychological variables				
Subjective knowledge	0.14***	0.00	0.01	0.03
	(0.03)	(0.01)	(0.01)	(0.03)
Objective knowledge	0.16***	0.05***	0.07***	0.25***
	(0.02)	(0.01)	(0.01)	(0.02)
Scientific consensus	0.49***	0.07***	0.06**	0.37***
	(0.05)	(0.01)	(0.02)	(0.05)
Trust in scientists	0.13*	0.05***	0.04	0.25***
	(0.05)	(0.02)	(0.03)	(0.06)
The New Ecological Paradigm (NEP)	0.30***	0.05***	0.08***	0.24***
	(0.03)	(0.01)	(0.02)	(0.03)
Individualism worldview	−0.02	−0.04***	−0.11***	−0.20***
	(0.02)	(0.01)	(0.01)	(0.02)
Egalitarianism worldview	0.07**	0.01	0.01	0.06*
	(0.03)	(0.01)	(0.01)	(0.03)
Personal experience (extreme weather events)	0.23***	0.01	0.05	0.13**
	(0.05)	(0.01)	(0.02)	(0.05)
Political ideology and Socio-Demographic variables				
Political ideology: Left	0.01	0.01	0.08***	−2.16***
	(0.03)	(0.01)	(0.02)	(0.18)
Political ideology: Progressive	0.10**	−0.03**	0.00	0.10**
	(0.03)	(0.01)	(0.02)	(0.03)
Female	0.00	0.00	−0.03	−0.02
	(0.03)	(0.01)	(0.02)	(0.03)
Education: High school or equivalent	0.01	0.05***	0.08***	0.21***
	(0.04)	(0.01)	(0.02)	(0.05)
Education: Undergraduate or more	0.03	0.07***	0.14***	0.31***
	(0.05)	(0.01)	(0.02)	(0.05)
Religion: Catholic	0.02	−0.00	−0.06	−0.02
	(0.07)	(0.02)	(0.04)	(0.07)
Religion: Evangelical Pentecostal or other evangelical	0.17	−0.08**	−0.13**	−0.29**
	(0.09)	(0.03)	(0.05)	(0.10)
Religion: Evangelical Traditional	0.09	0.03	−0.08	0.06
	(0.08)	(0.02)	(0.04)	(0.09)
Religion: Others/No Relig.	0.01	−0.01	−0.05	−0.06
	(0.07)	(0.02)	(0.04)	(0.08)
Income: 1 - 2 minimum wages	0.12*	0.01	0.06*	0.10
	(0.05)	(0.01)	(0.02)	(0.05)
Income: 2 - 3 minimum wages	0.01	0.01	0.08**	0.07
	(0.06)	(0.02)	(0.03)	(0.07)
Income: 3 - 5 minimum wages	−0.04	−0.00	0.07*	0.09
	(0.06)	(0.02)	(0.03)	(0.06)
Income: 5 - 10 minimum wages	0.02	−0.01	0.07	0.04
	(0.07)	(0.02)	(0.04)	(0.06)
Income: 10 minimum wages or more	0.04	0.00	0.09*	0.03
	(0.07)	(0.02)	(0.03)	(0.07)
Income: Do not know/ Prefer to not answer	0.02	0.01	0.01	0.01
	(0.05)	(0.02)	(0.03)	(0.06)
Age (Years)	0.00	−0.00	0.00*	−0.00
	(0.00)	(0.00)	(0.00)	(0.00)
Race: Black	−0.22**	−0.01	0.05	−0.04
	(0.08)	(0.02)	(0.04)	(0.08)
Const	4.50***	0.54***	0.17	−2.16***
	(0.18)	(0.05)	(0.09)	(0.18)
Fit statistics				
Num. obs.	2887	2883	2887	2883
Var: Country (Intercept)	0.00	0.00	0.00	0.00
Var: Residual	1.37	0.11	0.36	1.46

Notes: Results from four multilevel (hierarchical) models. Columns “Existence”, “Causes”, “Impacts”, "Belief (Index)" are models regressing climate change existence, perception of climate change causes, perception of climate change impacts, and standardized climate change belief index. respectively, on a set of independent variables and socio-demographic characteristics. A random intercept is specified at the country level. Coefficients for column “Existence” are increases in the climate change existence scale (i.e., 0-8) given a unit increase in the covariates. The higher the scale, the greater the confidence that climate change is happening. Coefficients for column “Causes” are percent increases in the probability of believing climate change is mainly caused by human activity given a unit increase in the covariates. Coefficients for column “Impacts” are percent increases in the probability of believing climate change impacts will be negative given a unit increase in the covariates. Coefficients for column "Belief (Index) are changes in the climate change belief index given a standard deviation increase in the covariates. The higher the index score, the greater the confidence that climate change is happening, and the higher the perception that it is caused by human activity and that the impacts will be negative. Reference baseline for Education is “Elementary (Primary) or less”, Religion is “Atheist” and Income is “0 – 1 minimum wages”. Signif. Codes: ***: 0.01, **: 0.05, *: 0.1. p-values from standard two-sided t-tests for the null hypothesis of a zero average parameter using the reported standard errors in parentheses.

Table 30: Fixed Effects Model Results

	Dependent Variable:			
	Existence (i)	Anthropogenic Causes (ii)	Consequences (iii)	Belief (Index) (iv)
Psychological variables				
Subjective knowledge	0.137***	0.003	0.013	0.027
	(0.034)	(0.009)	(0.015)	(0.033)
Objective knowledge	0.163***	0.053***	0.066***	0.251***
	(0.039)	(0.011)	(0.015)	(0.041)
Scientific consensus	0.485***	0.071***	0.064**	0.374***
	(0.067)	(0.019)	(0.027)	(0.068)
Trust in scientists	0.134*	0.053*	0.041	0.247**
	(0.074)	(0.028)	(0.034)	(0.099)
The New Ecological Paradigm (NEP)	0.299***	0.045***	0.077***	0.239***
	(0.046)	(0.015)	(0.019)	(0.050)
Individualism worldview	-0.016	-0.037***	-0.114***	-0.196***
	(0.026)	(0.008)	(0.015)	(0.028)
Egalitarianism worldview	0.076**	0.010	0.009	0.059*
	(0.031)	(0.008)	(0.016)	(0.030)
Personal experience (extreme weather events)	0.223***	0.013	0.045*	0.126**
	(0.061)	(0.015)	(0.027)	(0.059)
Political ideology and Socio-Demographic variables				
Political ideology: Left	0.010	0.014	0.080***	0.100**
	(0.036)	(0.012)	(0.020)	(0.044)
Political ideology: Progressive	0.098***	-0.027**	0.002	-0.060
	(0.032)	(0.012)	(0.019)	(0.042)
Female	0.003	0.001	-0.033*	-0.019
	(0.033)	(0.010)	(0.020)	(0.038)
Education: High school or equivalent	0.014	0.053***	0.083***	0.232***
	(0.051)	(0.018)	(0.027)	(0.064)
Education: Undergraduate or more	0.026	0.075***	0.144***	0.322***
	(0.054)	(0.018)	(0.028)	(0.065)
Religion: Catholic	0.016	0.002	-0.058	-0.008
	(0.077)	(0.017)	(0.036)	(0.078)
Religion: Evangelical Pentecostal or other evangelical	0.191*	-0.079*	-0.126**	-0.272*
	(0.113)	(0.044)	(0.054)	(0.163)
Religion: Evangelical Traditional	0.099	0.033	-0.073	0.071
	(0.091)	(0.021)	(0.045)	(0.089)
Religion: Others/No Relig.	0.018	-0.010	-0.049	-0.051
	(0.079)	(0.018)	(0.037)	(0.079)
Income: 1 - 2 minimum wages	0.120**	0.008	0.053*	0.092
	(0.047)	(0.018)	(0.029)	(0.062)
Income: 10 minimum wages or more	0.045	-0.003	0.091**	0.056
	(0.075)	(0.020)	(0.036)	(0.079)
Income: 2 - 3 minimum wages	0.012	0.012	0.083***	0.085
	(0.060)	(0.018)	(0.032)	(0.066)
Income: 3 - 5 minimum wages	-0.034	-0.006	0.065**	0.032
	(0.065)	(0.021)	(0.032)	(0.073)
Income: 5 - 10 minimum wages	0.020	-0.007	0.068	0.021
	(0.080)	(0.024)	(0.043)	(0.091)
Income: Do not know/ Prefer to not answer	0.021	0.004	0.007	0.002
	(0.062)	(0.018)	(0.033)	(0.067)
Age (Years)	0.002	-0.0003	0.001*	9.41×10^{-7}
	(0.001)	(0.0005)	(0.0007)	(0.002)
Race: Black	-0.214**	-0.008	0.050	-0.038
	(0.108)	(0.029)	(0.043)	(0.103)
Fixed-effects				
Country	Yes	Yes	Yes	Yes
Fit statistics				
Observations	2,887	2,883	2,887	2,883
R^2	0.20327	0.12895	0.15375	0.21842
Within R^2	0.19667	0.12649	0.14015	0.21181

Notes: Results from four fixed effects models (OLS). Columns "Existence", "Causes", "Impacts", and "Belief (Index)" are models regressing climate change existence, perception of climate change causes, perception of climate change impacts, and standardized climate change belief index. respectively, on a set of independent variables and socio-demographic characteristics. Fixed effects are included to control for anything constant in observations of the same country. Coefficients for column "Existence" are increases in the climate change belief scale (i.e., 0-8) given a unit increase in the covariates. The higher the scale, the greater the confidence that climate change is happening. Coefficients for column "Causes" are percent increases in the probability of believing climate change is mainly caused by human activity given a unit increase in the covariates. Coefficients for column "Impacts" are percent increases in the probability of believing climate change impacts will be negative given a unit increase in the covariates. Coefficients for column "Belief (Index) are changes in the climate change belief index given a standard deviation increase in the covariates. The higher the index score, the greater the confidence that climate change is happening, and the higher the perception that it is caused by human activity and that the impacts will be negative. Heteroskedasticity-robust standard errors in parentheses. Reference baseline for Education is "Elementary (Primary) or less", Religion is "Atheist" and Income is "0 – 1 minimum wages". Signif. Codes: ***: 0.01, **: 0.05, *: 0.1. p-values from standard two-sided t-tests for the null hypothesis of a zero average parameter using the reported standard errors in parentheses. p-values from standard two-sided t-tests for the null hypothesis of a zero average parameter using the reported standard errors in parentheses.

Table 31: Ordinal Logit Results - Existence of Climate Change

	Dependent Variable: Existence of Climate Change							
	Argentina	Brazil	Chile	Colombia	Ecuador	Mexico	Peru	Overall
Psychological variables								
Subjective knowledge	0.56***	0.44***	0.37*	0.57**	0.95***	1.10***	0.69***	0.61***
	(0.16)	(0.11)	(0.16)	(0.19)	(0.16)	(0.16)	(0.14)	(0.05)
Objective knowledge	0.27*	0.00	0.30**	0.59***	0.36**	0.03	0.39***	0.26***
	(0.13)	(0.11)	(0.11)	(0.15)	(0.12)	(0.13)	(0.09)	(0.04)
Scientific consensus	0.62*	1.06***	1.59***	0.99***	0.52*	0.23	0.83***	0.82***
	(0.24)	(0.21)	(0.24)	(0.26)	(0.23)	(0.23)	(0.21)	(0.08)
Trust in scientists	0.41	−0.58	0.75*	−0.21	0.73**	0.23	−0.27	0.09
	(0.38)	(0.30)	(0.30)	(0.32)	(0.24)	(0.26)	(0.23)	(0.10)
The New Ecological Paradigm (NEP)	0.76***	1.65***	1.08***	0.33	0.79***	0.87***	0.56***	0.81***
	(0.20)	(0.17)	(0.18)	(0.20)	(0.17)	(0.16)	(0.13)	(0.06)
Individualism worldview	−0.04	−0.35**	0.29*	−0.16	0.12	0.06	−0.02	−0.03
	(0.14)	(0.12)	(0.14)	(0.17)	(0.13)	(0.13)	(0.12)	(0.05)
Egalitarianism worldview	−0.04	0.41**	0.30*	0.35*	−0.22	0.24	0.03	0.11*
	(0.14)	(0.14)	(0.14)	(0.16)	(0.14)	(0.14)	(0.12)	(0.05)
Personal experience (extreme weather events)	0.24	−0.24	1.24***	0.23	0.12	1.11***	0.88***	0.48***
	(0.27)	(0.22)	(0.24)	(0.29)	(0.22)	(0.27)	(0.20)	(0.08)
Political ideology and Socio-Demographic variables								
Political ideology: Left	−0.14	−0.26	0.02	−0.19	0.39*	0.25	0.11	0.11
	(0.21)	(0.18)	(0.19)	(0.23)	(0.17)	(0.17)	(0.15)	(0.06)
Political ideology: Progressive	0.41*	0.64***	0.38	0.23	0.62***	0.38*	0.03	0.39***
	(0.21)	(0.19)	(0.20)	(0.21)	(0.18)	(0.17)	(0.16)	(0.07)
Female	−0.30	−0.21	0.09	−0.41*	0.01	0.14	0.11	−0.08
	(0.21)	(0.18)	(0.21)	(0.20)	(0.16)	(0.17)	(0.18)	(0.06)
Education: High school or equivalent	0.40	−0.39	0.54*	−0.40	0.64*	0.25	−0.26	−0.15
	(0.29)	(0.20)	(0.27)	(0.26)	(0.31)	(0.21)	(0.32)	(0.08)
Education: Undergraduate or more	0.39	−0.29	0.40	−0.62*	0.51	0.34	0.41	0.01
	(0.27)	(0.25)	(0.33)	(0.27)	(0.32)	(0.22)	(0.34)	(0.08)
Religion: Catholic	−0.25	−0.01	−0.92*	0.78	0.17	−0.04	0.24	−0.12
	(0.30)	(0.57)	(0.42)	(0.48)	(0.58)	(0.38)	(0.28)	(0.14)
Religion: Evangelical Pentecostal or other evangelical	0.12	0.93	−0.68	1.65*	0.97	0.37	0.72	0.40*
	(0.65)	(0.59)	(0.62)	(0.75)	(0.69)	(0.55)	(0.44)	(0.18)
Religion: Evangelical Traditional	−0.34	0.90	−0.96	0.81	0.84	0.54	0.61	0.23
	(0.41)	(0.60)	(0.49)	(0.55)	(0.63)	(0.52)	(0.36)	(0.16)
Religion: Others/No Relig.	−0.08	0.28	−1.22**	0.75	0.86	−0.36	0.97**	−0.01
	(0.33)	(0.57)	(0.43)	(0.49)	(0.60)	(0.39)	(0.34)	(0.14)
Income: 1 - 2 minimum wages	−0.31	−0.10	−0.17	0.44	0.77***	0.23	0.15	0.29**
	(0.35)	(0.24)	(0.27)	(0.25)	(0.23)	(0.38)	(0.22)	(0.09)
Income: 2 - 3 minimum wages	−0.71	−0.10	−0.73*	1.11**	0.61	0.21	−0.20	0.04
	(0.37)	(0.30)	(0.30)	(0.40)	(0.32)	(0.36)	(0.27)	(0.11)
Income: 3 - 5 minimum wages	−0.46	−0.75*	−0.49	0.46	0.34	−1.40**	−0.31	−0.12
	(0.35)	(0.30)	(0.32)	(0.38)	(0.31)	(0.50)	(0.26)	(0.11)
Income: 5 - 10 minimum wages	−1.50***	−0.20	0.55	0.16	0.23	1.44*	−0.17	−0.01
	(0.39)	(0.38)	(0.39)	(0.62)	(0.40)	(0.62)	(0.30)	(0.13)
Income: 10 minimum wages or more	−0.29	−0.05	0.85	1.31	−0.26	0.32	0.62	0.17
	(0.45)	(0.45)	(0.68)	(1.20)	(0.54)	(0.22)	(0.44)	(0.13)
Income: Do not know/ Prefer to not answer	−0.63	−0.55	−0.32	0.35	0.48*	0.55*	−0.56*	0.07
	(0.37)	(0.32)	(0.35)	(0.32)	(0.24)	(0.22)	(0.28)	(0.10)
Age (Years)	0.02**	0.00	−0.01	0.01	0.01	−0.01	0.02***	0.01***
	(0.01)	(0.01)	(0.01)	(0.01)	(0.01)	(0.01)	(0.00)	(0.00)
Race: Black	−1.98***	−1.06***	−0.50	−0.25	−1.09**	0.68	0.23	−0.66***
	(0.58)	(0.25)	(0.76)	(0.35)	(0.39)	(0.59)	(0.46)	(0.14)
Fit statistics								
Log Likelihood	−479.76	−644.18	−507.89	−424.39	−640.13	−634.00	−715.80	−4337.85
Deviance	959.51	1288.35	1015.78	848.79	1280.26	1268.00	1431.60	8675.70

Notes: Results from ordinal logistic models regressing climate change existence (an ordered scale of 0-8) on a set of independent variables and socio-demographic characteristics. Columns labeled with country names are models for each separate country, while column "Overall" includes all observations. Coefficients are increases in the expected value of climate change existence on the log-odds scale given a unit increase in the covariates. Reference baseline for Education is "Elementary (Primary) or less", Religion is "Atheist" and Income is "0 – 1 minimum wages". Signif. Codes: ***: 0.01, **: 0.05, *: 0.1. p-values from standard two-sided z-tests for the null hypothesis of a zero average parameter using the reported standard errors in parentheses.

Table 32: Logit Results - Anthropogenic Causes of Climate Change

	Dependent Variable: Anthropogenic Causes of Climate Change							
	Argentina	Brazil	Chile	Colombia	Ecuador	Mexico	Peru	Overall
Psychological variables								
Subjective knowledge	0.743	-0.382	0.772*	1.57	0.299	-0.367	0.185	0.128
	(0.469)	(0.257)	(0.443)	(1.09)	(0.569)	(0.444)	(0.378)	(0.141)
Objective knowledge	0.504	0.257	0.562**	1.76***	0.129	0.317	0.493**	0.473***
	(0.594)	(0.205)	(0.281)	(0.615)	(0.484)	(0.265)	(0.218)	(0.100)
Scientific consensus	0.548	1.18**	0.535	0.988	0.842	1.30**	0.560	0.757***
	(0.939)	(0.477)	(0.591)	(1.45)	(0.720)	(0.541)	(0.607)	(0.206)
Trust in scientists	2.09**	0.514	0.439	-1.34	0.497	0.797	0.747	0.572**
	(0.984)	(0.518)	(0.683)	(3.91)	(0.764)	(0.564)	(0.500)	(0.269)
The New Ecological Paradigm (NEP)	0.554	0.848***	0.278	0.509	1.56***	0.416	0.753**	0.625***
	(0.631)	(0.293)	(0.493)	(1.30)	(0.535)	(0.458)	(0.294)	(0.168)
Individualism worldview	-1.74***	-0.687**	-1.28***	-3.00***	-0.156	-0.707*	-0.820***	-0.798***
	(0.569)	(0.345)	(0.385)	(0.772)	(0.481)	(0.419)	(0.304)	(0.152)
Egalitarianism worldview	0.675	0.522*	0.399	0.613	-0.323	0.397	0.235	0.243*
	(0.630)	(0.275)	(0.352)	(0.578)	(0.472)	(0.488)	(0.329)	(0.141)
Personal experience (extreme weather events)	1.69**	0.378	0.475	-14.7***	0.249	1.05	0.441	0.268
	(0.774)	(0.575)	(0.690)	(1.25)	(1.11)	(0.662)	(0.570)	(0.237)
Political ideology and Socio-Demographic variables								
Political ideology: Left	-1.13*	0.318	-0.156	0.314	1.04	1.51***	-0.311	0.302
	(0.679)	(0.455)	(0.520)	(1.30)	(0.912)	(0.535)	(0.425)	(0.209)
Political ideology: Progressive	0.655	-0.536	-0.479	0.047	-0.617	-0.598	-0.478	-0.400**
	(1.08)	(0.474)	(0.509)	(1.10)	(0.682)	(0.459)	(0.448)	(0.201)
Female	0.076	-0.170	-0.645	-0.107	2.41*	-1.28***	-0.471	-0.058
	(0.751)	(0.487)	(0.530)	(0.850)	(1.33)	(0.493)	(0.502)	(0.196)
Education: High school or equivalent	0.482	0.301	-0.864	-0.311	2.32**	1.69*	0.856	0.498**
	(0.997)	(0.526)	(0.794)	(1.30)	(0.963)	(0.988)	(0.669)	(0.224)
Education: Undergraduate or more	3.11*	0.226	0.684	0.772	2.13**	2.26**	0.518	1.15***
	(1.62)	(0.583)	(0.978)	(1.18)	(0.954)	(0.889)	(0.652)	(0.259)
Religion: Catholic	-18.9***	-14.7***	-0.979	2.51**	-15.1***	1.40	-0.500	-0.079
	(1.66)	(0.544)	(1.55)	(1.01)	(1.89)	(0.915)	(1.31)	(0.458)
Religion: Evangelical Pentecostal or other evangelical	-18.4***	-15.0***	-1.64	0.005	-16.8***	2.61	-1.76	-0.899
	(2.05)	(0.597)	(1.67)	(2.86)	(1.62)	(1.65)	(1.45)	(0.557)
Religion: Evangelical Traditional	-17.8***	-12.3***	0.021	1.33	-14.0***	17.4***	-0.833	0.522
	(1.72)	(0.869)	(1.69)	(2.55)	(1.78)	(0.921)	(1.36)	(0.556)
Religion: Others/No Relig.	-17.0***	-15.2***	-0.736	0.703	-14.8***	0.958	-0.439	-0.241
	(1.88)	(0.563)	(1.58)	(1.43)	(1.62)	(0.827)	(1.47)	(0.475)
Income: 1 - 2 minimum wages	-1.58	0.926	-1.29**	0.493	-0.932	15.3***	1.05	0.195
	(1.24)	(0.623)	(0.644)	(1.01)	(0.950)	(0.440)	(0.669)	(0.292)
Income: 2 - 3 minimum wages	-1.31	0.792	-1.47*	-0.052	0.456	-1.19	1.48*	0.298
	(1.65)	(0.801)	(0.786)	(1.52)	(1.90)	(0.914)	(0.886)	(0.369)
Income: 3 - 5 minimum wages	-1.02	0.062	-1.40	-1.01	0.157	1.09	0.741	0.092
	(1.32)	(0.945)	(0.883)	(2.18)	(1.40)	(1.09)	(0.708)	(0.370)
Income: 5 - 10 minimum wages	-3.89**	1.61*	-1.63	0.383	16.2***	0.087	1.14	0.094
	(1.92)	(0.924)	(1.03)	(3.48)	(1.32)	(1.19)	(0.885)	(0.403)
Income: 10 minimum wages or more	-4.19***	0.333	11.5***	16.0***	17.8***	-0.018	0.670	0.204
	(1.50)	(1.06)	(0.908)	(2.06)	(1.12)	(0.598)	(1.05)	(0.387)
Income: Do not know/ Prefer to not answer	0.843	0.890	-1.31*	1.26	-0.456	-0.163	1.10	0.128
	(1.02)	(0.864)	(0.793)	(2.33)	(1.11)	(0.546)	(0.900)	(0.290)
Age (Years)	0.038*	-0.003	0.018	-0.066	-0.004	-0.018	-0.020	-0.008
	(0.020)	(0.015)	(0.016)	(0.044)	(0.034)	(0.015)	(0.013)	(0.008)
Race: Black	17.0***	-0.678	-1.49	18.0***	0.503	-2.14**	0.665	-0.011
	(0.914)	(0.618)	(1.31)	(2.71)	(1.28)	(0.970)	(1.35)	(0.404)
Const	14.9***	13.1***	1.31	15.7***	8.70**	-1.31	-0.819	-1.09
	(3.06)	(1.82)	(2.67)	(2.94)	(4.22)	(2.82)	(2.70)	(0.980)
Fit statistics								
Observations	355	433	431	382	250	485	547	2,883
Squared Correlation	0.31704	0.22013	0.20470	0.45880	0.33065	0.24314	0.15050	0.11730
Pseudo R^2	0.43457	0.17945	0.25230	0.64325	0.39638	0.27778	0.21016	0.15691

Notes: Results from binary logistic models regressing the perception of climate change causes on a set of independent variables and socio-demographic characteristics. Columns labeled with country names are models for each separate country, while column "Overall" includes all observations. Coefficients are the amount of increase in the predicted log odds of believing climate change is mainly caused by human activity given a unit increase in the covariates. Heteroskedasticity-robust standard errors in parentheses. Reference baseline for Education is "Elementary (Primary) or less", Religion is "Atheist" and Income is "0 – 1 minimum wages". Signif. Codes: ***: 0.01, **: 0.05, *: 0.1. p-values from standard two-sided z-tests for the null hypothesis of a zero average parameter using the reported standard errors in parentheses.

Table 33: Logit Results - Consequences of Climate Change

	Dependent Variable: Consequences of Climate Change							
	Argentina	Brazil	Chile	Colombia	Ecuador	Mexico	Peru	Overall
Psychological variables								
Subjective knowledge	-0.073	0.277	0.051	-0.220	0.309	0.291	-0.142	0.079
	(0.251)	(0.179)	(0.235)	(0.280)	(0.345)	(0.225)	(0.192)	(0.081)
Objective knowledge	0.526**	0.600***	0.278	0.739***	0.368	-0.175	0.315**	0.308***
	(0.212)	(0.198)	(0.182)	(0.259)	(0.279)	(0.214)	(0.151)	(0.070)
Scientific consensus	0.641	0.264	0.963**	-0.006	-0.124	0.323	0.222	0.273**
	(0.418)	(0.321)	(0.374)	(0.456)	(0.494)	(0.341)	(0.326)	(0.128)
Trust in scientists	-0.771	0.219	0.300	-0.472	-0.415	0.876**	0.641*	0.181
	(0.732)	(0.410)	(0.505)	(0.620)	(0.523)	(0.390)	(0.348)	(0.165)
The New Ecological Paradigm (NEP)	0.245	0.547**	0.280	0.561*	0.237	0.377	0.523***	0.423***
	(0.334)	(0.257)	(0.269)	(0.329)	(0.349)	(0.246)	(0.203)	(0.096)
Individualism worldview	-0.971***	-0.592***	-0.653***	-1.12***	-0.473*	-0.600***	-0.520**	-0.642***
	(0.257)	(0.182)	(0.200)	(0.239)	(0.275)	(0.202)	(0.220)	(0.080)
Egalitarianism worldview	0.160	0.124	-0.062	0.331	0.485	0.004	-0.060	0.068
	(0.237)	(0.220)	(0.226)	(0.249)	(0.321)	(0.187)	(0.205)	(0.084)
Personal experience (extreme weather events)	0.192	0.186	0.543	0.038	0.397	0.078	0.350	0.232
	(0.440)	(0.331)	(0.379)	(0.484)	(0.446)	(0.460)	(0.327)	(0.141)
Political ideology and Socio-Demographic variables								
Political ideology: Left	-0.520	1.15***	0.636*	0.300	0.172	0.783***	0.211	0.455***
	(0.339)	(0.275)	(0.329)	(0.322)	(0.359)	(0.255)	(0.232)	(0.104)
Political ideology: Progressive	0.058	-0.233	-0.291	-0.163	0.314	0.408	0.060	0.082
	(0.338)	(0.273)	(0.277)	(0.319)	(0.376)	(0.263)	(0.265)	(0.107)
Female	0.093	-0.302	-0.202	-0.117	-0.208	0.092	-0.578**	-0.167
	(0.327)	(0.285)	(0.297)	(0.319)	(0.351)	(0.254)	(0.261)	(0.105)
Education: High school or equivalent	0.417	0.256	-0.305	0.468	1.39**	0.619*	0.041	0.370***
	(0.455)	(0.302)	(0.418)	(0.388)	(0.664)	(0.326)	(0.539)	(0.123)
Education: Undergraduate or more	1.11**	0.718*	-0.049	0.404	2.14***	0.637**	0.167	0.769***
	(0.451)	(0.369)	(0.483)	(0.381)	(0.693)	(0.318)	(0.559)	(0.133)
Religion: Catholic	-0.251	-0.067	-1.14	-0.467	0.112	-0.597	-0.711	-0.552**
	(0.516)	(0.810)	(0.718)	(0.813)	(1.41)	(0.618)	(0.598)	(0.262)
Religion: Evangelical Pentecostal or other evangelical	-0.649	-0.087	-1.52*	-2.13*	0.934	-0.355	-1.18	-0.970***
	(0.729)	(0.845)	(0.893)	(1.21)	(1.55)	(0.844)	(0.788)	(0.316)
Religion: Evangelical Traditional	-0.926	0.453	-1.71**	-0.612	0.355	-1.14	-0.258	-0.617**
	(0.622)	(0.845)	(0.796)	(0.888)	(1.45)	(0.826)	(0.690)	(0.291)
Religion: Others/No Relig.	-0.059	0.559	-1.47**	0.188	-0.145	-0.554	-0.618	-0.506*
	(0.570)	(0.826)	(0.721)	(0.886)	(1.42)	(0.628)	(0.648)	(0.271)
Income: 1 - 2 minimum wages	-0.577	-0.142	0.059	0.140	0.507	0.592	0.640**	0.295**
	(0.546)	(0.339)	(0.401)	(0.404)	(0.465)	(0.647)	(0.325)	(0.144)
Income: 2 - 3 minimum wages	0.034	0.344	0.163	1.04**	0.394	-0.180	0.979**	0.432**
	(0.626)	(0.434)	(0.457)	(0.519)	(0.605)	(0.558)	(0.404)	(0.178)
Income: 3 - 5 minimum wages	0.025	-0.550	0.495	0.438	1.77**	-0.914	0.452	0.377**
	(0.564)	(0.465)	(0.500)	(0.642)	(0.747)	(0.757)	(0.366)	(0.185)
Income: 5 - 10 minimum wages	-0.628	1.46**	0.551	-0.601	2.34*	0.037	0.028	0.384
	(0.652)	(0.617)	(0.585)	(1.07)	(1.27)	(0.638)	(0.557)	(0.256)
Income: 10 minimum wages or more	-0.856	0.261	1.35	-0.994	-0.547	0.758**	1.05	0.444**
	(0.710)	(0.651)	(1.35)	(1.22)	(0.861)	(0.326)	(0.733)	(0.195)
Income: Do not know/ Prefer to not answer	-0.923	-0.136	0.104	-0.633	-0.165	0.162	0.854**	0.046
	(0.585)	(0.418)	(0.524)	(0.532)	(0.527)	(0.324)	(0.412)	(0.157)
Age (Years)	0.018*	0.015*	0.011	-0.017	-0.013	0.018**	0.005	0.006*
	(0.010)	(0.008)	(0.010)	(0.011)	(0.013)	(0.008)	(0.009)	(0.004)
Race: Black	14.4***	0.271	-0.788	-0.260	0.697	1.80**	0.308	0.260
	(0.381)	(0.379)	(1.09)	(0.546)	(0.952)	(0.851)	(0.713)	(0.224)
Const	0.736	-4.85***	-0.008	0.716	-3.83	-2.02	-0.937	-1.47**
	(1.70)	(1.50)	(1.53)	(1.82)	(2.40)	(1.42)	(1.65)	(0.591)
Fit statistics								
Observations	355	434	434	382	250	485	547	2,887
Squared Correlation	0.18320	0.27506	0.22687	0.15923	0.16352	0.20962	0.14915	0.15407
Pseudo R^2	0.12883	0.13598	0.13822	0.20280	0.21007	0.10235	0.06547	0.08382

Notes: Results from binary logistic models regressing the perception of climate change impacts on a set of independent variables and socio-demographic characteristics. Columns labeled with country names are models for each separate country, while column "Overall" includes all observations. Coefficients are the amount of increase in the predicted log odds of believing climate change impacts will be negative given a unit increase in the covariates. Heteroskedasticity-robust standard errors in parentheses. Reference baseline for Education is "Elementary (Primary) or less", Religion is "Atheist" and Income is "0 – 1 minimum wages". Signif. Codes: ***: 0.01, **: 0.05, *: 0.1. p-values from standard two-sided z-tests for the null hypothesis of a zero average parameter using the reported standard errors in parentheses.

Table 34: Stepwise OLS Results - Correlates of Belief in the Existence of Climate Change

Dependent Variable:	Existence of Climate Change				
Model:	(1)	(2)	(3)	(4)	(5)
Psychological variables					
Subjective knowledge	0.212***	0.167***	0.165***	0.143***	0.140***
	(0.033)	(0.032)	(0.032)	(0.033)	(0.033)
Objective knowledge	0.293***	0.231***	0.221***	0.165***	0.165***
	(0.040)	(0.038)	(0.037)	(0.039)	(0.038)
Individualism worldview	-0.033	-0.008	-0.008	-0.016	-0.016
	(0.026)	(0.025)	(0.025)	(0.026)	(0.026)
Egalitarianism worldview	0.141***	0.112***	0.107***	0.066**	0.067**
	(0.032)	(0.030)	(0.030)	(0.031)	(0.030)
Political ideology and Socio-Demographic variables					
Political ideology: Left	0.032	0.021	0.021	0.022	0.019
	(0.037)	(0.036)	(0.036)	(0.036)	(0.036)
Political ideology: Progressive	0.115***	0.112***	0.112***	0.099***	0.102***
	(0.033)	(0.032)	(0.032)	(0.032)	(0.032)
Female	0.001	0.012	0.014	0.009	0.007
	(0.035)	(0.034)	(0.034)	(0.034)	(0.034)
Education: High school or equivalent	0.028	0.016	0.014	-0.001	0.013
	(0.048)	(0.046)	(0.046)	(0.047)	(0.047)
Education: Undergraduate or more	0.074	0.045	0.038	0.031	0.034
	(0.054)	(0.051)	(0.051)	(0.051)	(0.051)
Religion: Catholic	-0.013	0.003	0.006	0.019	0.015
	(0.079)	(0.074)	(0.074)	(0.078)	(0.076)
Religion: Evangelical Pentecostal or other evangelical	0.050	0.086	0.108	0.138	0.138
	(0.109)	(0.105)	(0.106)	(0.110)	(0.110)
Religion: Evangelical Traditional	0.051	0.063	0.077	0.093	0.087
	(0.095)	(0.089)	(0.088)	(0.092)	(0.091)
Religion: Others/No Relig.	-0.025	0.019	0.028	0.019	0.005
	(0.084)	(0.079)	(0.078)	(0.081)	(0.080)
Income: 1 - 2 minimum wages	0.079*	0.110**	0.100**	0.129***	0.121***
	(0.047)	(0.046)	(0.046)	(0.047)	(0.046)
Income: 10 minimum wages or more	0.043	0.042	0.036	0.054	0.045
	(0.069)	(0.067)	(0.068)	(0.068)	(0.068)
Income: 2 - 3 minimum wages	0.027	0.014	0.0004	0.005	0.002
	(0.062)	(0.060)	(0.060)	(0.060)	(0.060)
Income: 3 - 5 minimum wages	-0.074	-0.052	-0.065	-0.050	-0.039
	(0.064)	(0.062)	(0.063)	(0.063)	(0.065)
Income: 5 - 10 minimum wages	-0.032	-0.006	-0.025	0.013	0.010
	(0.082)	(0.080)	(0.080)	(0.081)	(0.080)
Income: Do not know/ Prefer to not answer	-0.017	-0.0001	-0.013	0.019	0.028
	(0.061)	(0.060)	(0.061)	(0.061)	(0.060)
Age (Years)	0.002**	0.002	0.002*	0.001	0.002
	(0.001)	(0.0009)	(0.0010)	(0.0010)	(0.0010)
Race: Black	-0.247**	-0.241**	-0.241**	-0.239**	-0.235**
	(0.113)	(0.107)	(0.106)	(0.105)	(0.105)
Climate-related independent variables					
Scientific consensus		0.576***	0.559***	0.494***	0.486***
		(0.069)	(0.069)	(0.068)	(0.067)
Trust in scientists			0.158**	0.120	0.122*
			(0.072)	(0.074)	(0.073)
The New Ecological Paradigm (NEP)				0.311***	0.305***
				(0.047)	(0.046)
Personal experience (extreme weather events)					0.232***
					(0.060)
Const	5.55***	5.41***	5.33***	4.67***	4.50***
	(0.208)	(0.205)	(0.214)	(0.254)	(0.261)
Fit statistics					
Observations	2,959	2,959	2,959	2,887	2,887
R^2	0.12520	0.17151	0.17400	0.19162	0.19881

Notes:Results from stepwise ordinary least squares models regressing climate change existence on a set of independent variables and socio-demographic characteristics. We gradually include climate-related independent variables from columns 2 - 5. Coefficients are changes in the climate change existence scale (i.e., 0-8) given a unit increase in the covariates. The higher the scale, the greater the confidence that climate change is happening. Heteroskedasticity-robust standard errors in parentheses. Reference baseline for Education is “Elementary (Primary) or less”, Religion is “Atheist” and Income is “0 – 1 minimum wages”. Signif. Codes: ***: 0.01, **: 0.05, *: 0.1. p-values from standard two-sided t-tests for the null hypothesis of a zero average parameter using the reported standard errors in parentheses.

Table 35: Stepwise OLS Results - Correlates of Belief in the Anthropogenic Causes of Climate Change

Dependent Variable:	Anthropogenic Climate Change				
Model:	(1)	(2)	(3)	(4)	(5)
Psychological variables					
Subjective knowledge	0.011	0.005	0.004	0.002	0.002
	(0.009)	(0.008)	(0.008)	(0.008)	(0.009)
Objective knowledge	0.076***	0.067***	0.062***	0.054***	0.054***
	(0.011)	(0.011)	(0.011)	(0.011)	(0.011)
Individualism worldview	-0.041***	-0.037***	-0.037***	-0.037***	-0.038***
	(0.008)	(0.008)	(0.008)	(0.008)	(0.008)
Egalitarianism worldview	0.022***	0.018**	0.016**	0.010	0.010
	(0.008)	(0.008)	(0.008)	(0.008)	(0.008)
Political ideology and Socio-Demographic variables					
Political ideology: Left	0.016	0.014	0.014	0.015	0.015
	(0.012)	(0.012)	(0.012)	(0.012)	(0.012)
Political ideology: Progressive	-0.024**	-0.024**	-0.024**	-0.026**	-0.025**
	(0.012)	(0.012)	(0.012)	(0.011)	(0.011)
Female	-0.002	-0.0005	7.58×10^{-5}	0.002	0.002
	(0.011)	(0.011)	(0.011)	(0.010)	(0.011)
Education: High school or equivalent	0.048***	0.046***	0.045***	0.040**	0.041**
	(0.016)	(0.016)	(0.016)	(0.016)	(0.016)
Education: Undergraduate or more	0.074***	0.070***	0.067***	0.064***	0.064***
	(0.016)	(0.016)	(0.016)	(0.016)	(0.016)
Religion: Catholic	-0.004	-0.002	-0.0004	-0.001	-0.001
	(0.016)	(0.016)	(0.016)	(0.016)	(0.016)
Religion: Evangelical Pentecostal or other evangelical	-0.094**	-0.089**	-0.080*	-0.081*	-0.081*
	(0.044)	(0.044)	(0.041)	(0.043)	(0.043)
Religion: Evangelical Traditional	0.021	0.023	0.029	0.030	0.030
	(0.020)	(0.020)	(0.020)	(0.020)	(0.020)
Religion: Others/No Relig.	-0.022	-0.016	-0.012	-0.010	-0.011
	(0.018)	(0.017)	(0.017)	(0.018)	(0.018)
Income: 1 - 2 minimum wages	0.003	0.008	0.004	0.010	0.010
	(0.018)	(0.018)	(0.018)	(0.018)	(0.018)
Income: 10 minimum wages or more	0.008	0.008	0.005	0.007	0.006
	(0.021)	(0.020)	(0.020)	(0.020)	(0.020)
Income: 2 - 3 minimum wages	0.021	0.020	0.014	0.015	0.015
	(0.019)	(0.019)	(0.018)	(0.018)	(0.018)
Income: 3 - 5 minimum wages	-0.014	-0.011	-0.016	-0.004	-0.004
	(0.022)	(0.022)	(0.021)	(0.021)	(0.021)
Income: 5 - 10 minimum wages	-0.005	-0.002	-0.009	-0.004	-0.004
	(0.025)	(0.025)	(0.024)	(0.024)	(0.024)
Income: Do not know/ Prefer to not answer	0.009	0.011	0.006	0.007	0.007
	(0.019)	(0.019)	(0.018)	(0.018)	(0.018)
Age (Years)	-0.0003	-0.0004	-0.0004	-0.0004	-0.0004
	(0.0005)	(0.0005)	(0.0005)	(0.0005)	(0.0005)
Race: Black	-0.012	-0.012	-0.011	-0.006	-0.005
	(0.031)	(0.030)	(0.030)	(0.029)	(0.029)
Climate-related independent variables					
Scientific consensus		0.085***	0.078***	0.070***	0.070***
		(0.018)	(0.019)	(0.019)	(0.019)
Trust in scientists			0.066**	0.056*	0.056**
			(0.027)	(0.028)	(0.028)
The New Ecological Paradigm (NEP)				0.045***	0.045***
				(0.015)	(0.015)
Personal experience (extreme weather events)					0.014
					(0.015)
Const	0.701***	0.680***	0.648***	0.549***	0.539***
	(0.056)	(0.056)	(0.057)	(0.070)	(0.071)
Fit statistics					
Observations	2,953	2,953	2,953	2,883	2,883
R^2	0.10036	0.11401	0.12001	0.12554	0.12592

Notes: Results from stepwise ordinary least squares (linear probability) models regressing the perception of climate change causes on a set of independent variables and socio-demographic characteristics. We gradually include climate-related independent variables from columns 2 - 5. Coefficients multiplied by one hundred are percentage point changes in the probability of believing climate change is mainly caused by human activity given a unit increase in the covariates. Heteroskedasticity-robust standard errors in parentheses. Reference baseline for Education is "Elementary (Primary) or less", Religion is "Atheist" and Income is "0 – 1 minimum wages". Signif. Codes: ***: 0.01, **: 0.05, *: 0.1. p-values from standard two-sided t-tests for the null hypothesis of a zero average parameter using the reported standard errors in parentheses.

Table 36: Stepwise OLS Results - Correlates of Belief in the Consequences of Climate Change

Dependent Variable:	Consequences of Climate Change				
Model:	(1)	(2)	(3)	(4)	(5)
Psychological variables					
Subjective knowledge	0.029*	0.021	0.021	0.014	0.013
	(0.015)	(0.015)	(0.015)	(0.015)	(0.015)
Objective knowledge	0.090***	0.079***	0.076***	0.067***	0.067***
	(0.014)	(0.014)	(0.014)	(0.015)	(0.015)
Individualism worldview	-0.121***	-0.117***	-0.117***	-0.115***	-0.115***
	(0.014)	(0.014)	(0.014)	(0.014)	(0.014)
Egalitarianism worldview	0.025*	0.020	0.019	0.004	0.005
	(0.015)	(0.015)	(0.015)	(0.016)	(0.016)
Political ideology and Socio-Demographic variables					
Political ideology: Left	0.083***	0.081***	0.081***	0.085***	0.085***
	(0.019)	(0.019)	(0.019)	(0.020)	(0.020)
Political ideology: Progressive	0.015	0.014	0.014	0.007	0.008
	(0.019)	(0.019)	(0.019)	(0.019)	(0.019)
Female	-0.030	-0.028	-0.028	-0.031	-0.031
	(0.019)	(0.019)	(0.019)	(0.019)	(0.020)
Education: High school or equivalent	0.083***	0.082***	0.081***	0.076***	0.079***
	(0.025)	(0.025)	(0.025)	(0.025)	(0.025)
Education: Undergraduate or more	0.151***	0.146***	0.144***	0.145***	0.145***
	(0.026)	(0.026)	(0.026)	(0.026)	(0.026)
Religion: Catholic	-0.066*	-0.063*	-0.062*	-0.066*	-0.067*
	(0.035)	(0.034)	(0.034)	(0.035)	(0.034)
Religion: Evangelical Pentecostal or other evangelical	-0.170***	-0.164***	-0.156***	-0.155***	-0.155***
	(0.053)	(0.052)	(0.051)	(0.052)	(0.052)
Religion: Evangelical Traditional	-0.086*	-0.084*	-0.079*	-0.081*	-0.082*
	(0.044)	(0.043)	(0.044)	(0.044)	(0.044)
Religion: Others/No Relig.	-0.062*	-0.054	-0.052	-0.057	-0.059*
	(0.036)	(0.036)	(0.036)	(0.036)	(0.036)
Income: 1 - 2 minimum wages	0.053*	0.059**	0.056**	0.062**	0.061**
	(0.028)	(0.028)	(0.028)	(0.028)	(0.028)
Income: 10 minimum wages or more	0.086**	0.086**	0.083**	0.084**	0.082**
	(0.034)	(0.034)	(0.034)	(0.034)	(0.034)
Income: 2 - 3 minimum wages	0.089***	0.087***	0.083***	0.085***	0.084***
	(0.032)	(0.032)	(0.032)	(0.032)	(0.032)
Income: 3 - 5 minimum wages	0.063**	0.067**	0.063**	0.067**	0.069**
	(0.032)	(0.032)	(0.032)	(0.032)	(0.032)
Income: 5 - 10 minimum wages	0.064	0.069*	0.063	0.070*	0.070
	(0.042)	(0.041)	(0.041)	(0.042)	(0.043)
Income: Do not know/ Prefer to not answer	0.002	0.005	0.001	0.006	0.008
	(0.032)	(0.032)	(0.032)	(0.032)	(0.032)
Age (Years)	0.001*	0.001	0.001*	0.001*	0.001*
	(0.0007)	(0.0007)	(0.0006)	(0.0007)	(0.0007)
Race: Black	0.030	0.031	0.031	0.046	0.047
	(0.044)	(0.043)	(0.043)	(0.043)	(0.043)
Climate-related independent variables					
Scientific consensus		0.095***	0.089***	0.063**	0.062**
		(0.027)	(0.027)	(0.027)	(0.027)
Trust in scientists			0.051	0.039	0.040
			(0.034)	(0.035)	(0.035)
The New Ecological Paradigm (NEP)				0.082***	0.081***
				(0.019)	(0.019)
Personal experience (extreme weather events)					0.046*
					(0.027)
Const	0.429***	0.405***	0.381***	0.206*	0.173
	(0.092)	(0.092)	(0.094)	(0.108)	(0.110)
Fit statistics					
Observations	2,959	2,959	2,959	2,887	2,887
R^2	0.13407	0.13915	0.14021	0.14658	0.14774

Notes: Results from stepwise ordinary least squares (linear probability) models regressing the perception of climate change consequences on a set of independent variables and socio-demographic characteristics. We gradually include climate-related independent variables from columns 2 - 5. Coefficients multiplied by one hundred are percentage point changes in the probability of believing climate change impacts will be negative given a unit increase in the covariates. Heteroskedasticity-robust standard errors in parentheses. Reference baseline for Education is "Elementary (Primary) or less", Religion is "Atheist" and Income is "0 – 1 minimum wages". Signif. Codes: ***: 0.01, **: 0.05, *: 0.1. p-values from standard two-sided t-tests for the null hypothesis of a zero average parameter using the reported standard errors in parentheses.

Table 37: Stepwise OLS Results - Correlates of Belief in the Existence of Climate Change

Dependent Variable:	Existence of Climate Change								
Model:	(1)	(2)	(3)	(4)	(5)	(6)	(7)	(8)	(9)
Significant variables									
Subjective knowledge	0.155***	0.155***	0.144***	0.144***	0.144***	0.144***	0.143***	0.143***	0.140***
	(0.031)	(0.031)	(0.033)	(0.033)	(0.033)	(0.033)	(0.033)	(0.034)	(0.033)
Objective knowledge	0.177***	0.170***	0.169***	0.169***	0.170***	0.169***	0.169***	0.166***	0.165***
	(0.037)	(0.036)	(0.038)	(0.038)	(0.038)	(0.038)	(0.038)	(0.038)	(0.038)
Scientific consensus	0.488***	0.475***	0.487***	0.487***	0.487***	0.486***	0.485***	0.490***	0.486***
	(0.063)	(0.062)	(0.067)	(0.067)	(0.068)	(0.067)	(0.067)	(0.067)	(0.067)
The New Ecological Paradigm (NEP)	0.298***	0.293***	0.299***	0.299***	0.299***	0.299***	0.302***	0.306***	0.305***
	(0.045)	(0.045)	(0.046)	(0.046)	(0.046)	(0.046)	(0.046)	(0.046)	(0.046)
Egalitarianism worldview	0.065**	0.063**	0.075***	0.074**	0.074**	0.076**	0.072**	0.070**	0.067**
	(0.027)	(0.027)	(0.028)	(0.029)	(0.029)	(0.030)	(0.031)	(0.030)	(0.030)
Personal experience (extreme weather events)	0.241***	0.242***	0.239***	0.239***	0.239***	0.238***	0.241***	0.232***	0.232***
	(0.056)	(0.056)	(0.058)	(0.058)	(0.059)	(0.059)	(0.059)	(0.060)	(0.060)
Political ideology: Progressive	0.099***	0.098***	0.097***	0.097***	0.098***	0.097***	0.100***	0.103***	0.102***
	(0.029)	(0.029)	(0.031)	(0.032)	(0.032)	(0.032)	(0.032)	(0.032)	(0.032)
Race: Black	-0.201**	-0.198**	-0.240**	-0.240**	-0.239**	-0.238**	-0.249**	-0.244**	-0.235**
	(0.096)	(0.096)	(0.103)	(0.103)	(0.103)	(0.105)	(0.105)	(0.105)	(0.105)
Insignificant variables									
Trust in scientists		0.124*	0.114	0.114	0.115	0.113	0.124*	0.120	0.122*
		(0.070)	(0.073)	(0.073)	(0.073)	(0.073)	(0.073)	(0.074)	(0.073)
Individualism worldview			-0.017	-0.017	-0.017	-0.016	-0.019	-0.018	-0.016
			(0.025)	(0.025)	(0.025)	(0.026)	(0.026)	(0.026)	(0.026)
Political ideology: Left				0.002	0.002	0.003	0.010	0.014	0.019
				(0.035)	(0.035)	(0.035)	(0.036)	(0.036)	(0.036)

Female					0.008	0.011	0.004	0.010	0.007
					(0.033)	(0.034)	(0.033)	(0.034)	(0.034)
Education: High school or equivalent						-0.004	9.19×10^{-5}	0.003	0.013
						(0.047)	(0.047)	(0.047)	(0.047)
Education: Undergraduate or more						0.011	0.021	0.031	0.034
						(0.049)	(0.050)	(0.051)	(0.051)
Religion: Catholic							0.017	0.023	0.015
							(0.080)	(0.077)	(0.076)
Religion: Evangelical Pentecostal or other evangelical							0.132	0.140	0.138
							(0.113)	(0.111)	(0.110)
Religion: Evangelical Traditional							0.085	0.087	0.087
							(0.094)	(0.092)	(0.091)
Religion: Others/No Relig.							-0.005	0.005	0.005
							(0.084)	(0.081)	(0.080)
Income: 1 - 2 minimum wages								0.131***	0.121***
								(0.046)	(0.046)
Income: 10 minimum wages or more								0.060	0.045
								(0.065)	(0.068)
Income: 2 - 3 minimum wages								0.012	0.002
								(0.058)	(0.060)
Income: 3 - 5 minimum wages								-0.018	-0.039
								(0.064)	(0.065)
Income: 5 - 10 minimum wages								0.031	0.010
								(0.077)	(0.080)
Income: Do not know/ Prefer to not answer								0.030	0.028
								(0.060)	(0.060)
Age (Years)									0.002
									(0.0010)
Const	4.66***	4.61***	4.63***	4.63***	4.62***	4.62***	4.58***	4.53***	4.50***
	(0.211)	(0.221)	(0.238)	(0.239)	(0.243)	(0.247)	(0.263)	(0.261)	(0.261)

Fit statistics									
Observations	3,117	3,117	2,887	2,887	2,887	2,887	2,887	2,887	2,887
R^2	0.18979	0.19136	0.19343	0.19343	0.19344	0.19349	0.19497	0.19811	0.19881

Notes: Results from stepwise ordinary least squares models regressing climate change existence on a set of independent variables and socio-demographic characteristics. We gradually include the insignificant variables (at the 95% level) from our main specification from columns 2 - 9. Coefficients are changes in the climate change existence scale (i.e., 0-8) given a unit increase in the covariates. The higher the scale, the greater the confidence that climate change is happening. Heteroskedasticity-robust standard errors in parentheses. Reference baseline for Education is "Elementary (Primary) or less", Religion is "Atheist" and Income is "0 – 1 minimum wages". Signif. Codes: ***: 0.01, **: 0.05, *: 0.1. p-values from standard two-sided t-tests for the null hypothesis of a zero average parameter using the reported standard errors in parentheses.

Table 38: Stepwise OLS Results - Correlates of Belief in the Anthropogenic Causes of Climate Change

Dependent Variable:	Anthropogenic Climate Change									
Model:	(1)	(2)	(3)	(4)	(5)	(6)	(7)	(8)	(9)	(10)
Significant variables										
Objective knowledge	0.053***	0.052***	0.053***	0.053***	0.052***	0.052***	0.053***	0.053***	0.054***	0.054***
	(0.011)	(0.011)	(0.011)	(0.011)	(0.011)	(0.011)	(0.011)	(0.011)	(0.011)	(0.011)
Scientific consensus	0.082***	0.082***	0.071***	0.071***	0.070***	0.070***	0.069***	0.069***	0.070***	0.070***
	(0.019)	(0.019)	(0.019)	(0.019)	(0.019)	(0.019)	(0.019)	(0.019)	(0.019)	(0.019)
Trust in scientists	0.062*	0.063*	0.061*	0.061*	0.061*	0.061*	0.057*	0.056*	0.056*	0.056**
	(0.032)	(0.032)	(0.033)	(0.033)	(0.033)	(0.033)	(0.030)	(0.029)	(0.028)	(0.028)
The New Ecological Paradigm (NEP)	0.052***	0.053***	0.048***	0.047***	0.047***	0.047***	0.045***	0.045***	0.045***	0.045***
	(0.014)	(0.014)	(0.015)	(0.015)	(0.014)	(0.014)	(0.015)	(0.015)	(0.015)	(0.015)
Individualism worldview	-	-	-	-	-	-	-	-	-	-
	0.038***	0.038***	0.039***	0.039***	0.037***	0.037***	0.037***	0.037***	0.038***	0.038***
	(0.007)	(0.007)	(0.007)	(0.007)	(0.007)	(0.008)	(0.007)	(0.008)	(0.008)	(0.008)
Political ideology: Progressive	-	-	-	-	-	-	-	-	-	-
	0.024**	0.023**	0.023**	0.023**	0.025**	0.025**	0.026**	0.026**	0.026**	0.025**
	(0.011)	(0.011)	(0.011)	(0.012)	(0.012)	(0.012)	(0.012)	(0.012)	(0.011)	(0.011)
Education: High school or equivalent	0.049***	0.048***	0.044**	0.045***	0.044**	0.044**	0.042**	0.043**	0.041**	0.041**
	(0.017)	(0.017)	(0.017)	(0.017)	(0.018)	(0.018)	(0.017)	(0.017)	(0.016)	(0.016)
Education: Undergraduate or more	0.063***	0.062***	0.063***	0.063***	0.064***	0.064***	0.063***	0.065***	0.064***	0.064***
	(0.015)	(0.015)	(0.016)	(0.016)	(0.016)	(0.015)	(0.015)	(0.016)	(0.015)	(0.016)
Insignificant variables										
Subjective knowledge		0.001	0.0007	0.0006	0.0008	0.0008	0.001	0.002	0.002	0.002
		(0.008)	(0.008)	(0.008)	(0.008)	(0.008)	(0.008)	(0.008)	(0.009)	(0.009)
Egalitarianism worldview			0.010	0.010	0.008	0.008	0.009	0.009	0.010	0.010
			(0.008)	(0.008)	(0.008)	(0.008)	(0.008)	(0.008)	(0.008)	(0.008)

Personal experience (extreme weather events)	0.017	0.016	0.016	0.015	0.015	0.014	0.014
	(0.014)	(0.014)	(0.014)	(0.014)	(0.015)	(0.015)	(0.015)
Political ideology: Left		0.018	0.018	0.016	0.016	0.015	0.015
		(0.011)	(0.011)	(0.011)	(0.011)	(0.012)	(0.012)
Female			-0.0004	0.001	0.0010	0.002	0.002
			(0.011)	(0.011)	(0.011)	(0.011)	(0.011)
Religion: Catholic				-0.004	-0.004	-0.001	-0.001
				(0.016)	(0.016)	(0.016)	(0.016)
Religion: Evangelical Pentecostal or other evangelical				-0.082*	-0.081*	-0.081*	-0.081*
				(0.044)	(0.043)	(0.043)	(0.043)
Religion: Evangelical Traditional				0.030	0.030	0.030	0.030
				(0.020)	(0.020)	(0.020)	(0.020)
Religion: Others/No Relig.				-0.011	-0.011	-0.011	-0.011
				(0.017)	(0.017)	(0.018)	(0.018)
Income: 1 - 2 minimum wages					0.007	0.010	0.010
					(0.016)	(0.018)	(0.018)
Income: 10 minimum wages or more					0.003	0.007	0.006
					(0.018)	(0.020)	(0.020)
Income: 2 - 3 minimum wages					0.013	0.015	0.015
					(0.017)	(0.018)	(0.018)
Income: 3 - 5 minimum wages					-0.009	-0.004	-0.004
					(0.018)	(0.021)	(0.021)
Income: 5 - 10 minimum wages					-0.009	-0.004	-0.004
					(0.023)	(0.024)	(0.024)
Income: Do not know/ Prefer to not answer					0.007	0.007	0.007
					(0.018)	(0.018)	(0.018)
Age (Years)						-0.0004	-0.0004
						(0.0005)	(0.0005)
Race: Black							-0.005
							(0.029)

Const	0.537***	0.535***	0.538***	0.525***	0.520***	0.520***	0.534***	0.530***	0.538***	0.539***
	(0.060)	(0.062)	(0.066)	(0.066)	(0.066)	(0.068)	(0.070)	(0.070)	(0.071)	(0.071)
Fit statistics										
Observations	3,037	3,031	2,883	2,883	2,883	2,883	2,883	2,883	2,883	2,883
R^2	0.12644	0.12525	0.11470	0.11521	0.11634	0.11634	0.12453	0.12525	0.12590	0.12592

Notes: Results from stepwise ordinary least squares (linear probability) models regressing the perception of climate change causes on a set of independent variables and socio-demographic characteristics. We gradually include the insignificant variables (at the 95% level) from our main specification from columns 2 - 9. Coefficients multiplied by one hundred are percentage point changes in the probability of believing climate change is mainly caused by human activity given a unit increase in the covariates. Heteroskedasticity-robust standard errors in parentheses. Reference baseline for Education is "Elementary (Primary) or less", Religion is "Atheist" and Income is "0 – 1 minimum wages". Signif. Codes: ***: 0.01, **: 0.05, *: 0.1. p-values from standard two-sided t-tests for the null hypothesis of a zero average parameter using the reported standard errors in parentheses.

Table 39: Stepwise OLS Results - Correlates of Belief in the Consequences of Climate Change

Dependent Variable:	Consequences of Climate Change										
Model:	(1)	(2)	(3)	(4)	(5)	(6)	(7)	(8)	(9)	(10)	(11)
Significant variables											
Objective knowledge	0.066***	0.066***	0.066***	0.069***	0.066***	0.068***	0.067***	0.068***	0.068***	0.066***	0.067***
	(0.011)	(0.011)	(0.011)	(0.011)	(0.011)	(0.012)	(0.012)	(0.014)	(0.015)	(0.014)	(0.015)
Scientific consensus	0.103***	0.102***	0.097***	0.092***	0.085***	0.081***	0.079***	0.065**	0.064**	0.062**	0.062**
	(0.021)	(0.021)	(0.021)	(0.021)	(0.022)	(0.022)	(0.022)	(0.027)	(0.027)	(0.027)	(0.027)
The New Ecological Paradigm (NEP)	0.094***	0.093***	0.093***	0.092***	0.089***	0.085***	0.085***	0.081***	0.081***	0.080***	0.081***
	(0.015)	(0.015)	(0.015)	(0.015)	(0.015)	(0.016)	(0.016)	(0.019)	(0.019)	(0.019)	(0.019)
Individualism worldview	-0.114***	-0.111***	-0.107***	-0.106***	-0.106***	-0.111***	-0.111***	-0.114***	-0.116***	-0.115***	-0.115***
	(0.012)	(0.012)	(0.012)	(0.012)	(0.012)	(0.012)	(0.012)	(0.014)	(0.014)	(0.014)	(0.014)
Political ideology: Left	0.084***	0.077***	0.081***	0.081***	0.081***	0.085***	0.085***	0.081***	0.081***	0.084***	0.085***
	(0.016)	(0.016)	(0.016)	(0.016)	(0.016)	(0.017)	(0.017)	(0.020)	(0.020)	(0.020)	(0.020)
Education: High school or equivalent	0.064***	0.061***	0.062***	0.058***	0.057***	0.052**	0.053***	0.070***	0.070***	0.077***	0.079***
	(0.020)	(0.019)	(0.019)	(0.019)	(0.019)	(0.020)	(0.020)	(0.025)	(0.025)	(0.025)	(0.025)
Education: Undergraduate or more	0.173***	0.162***	0.144***	0.140***	0.137***	0.135***	0.135***	0.146***	0.141***	0.144***	0.145***
	(0.020)	(0.019)	(0.020)	(0.020)	(0.020)	(0.021)	(0.021)	(0.025)	(0.026)	(0.026)	(0.026)
Religion: Catholic		-0.086***	-0.082***	-0.082***	-0.080***	-0.073**	-0.073**	-0.066*	-0.061*	-0.067*	-0.067*
		(0.031)	(0.030)	(0.030)	(0.030)	(0.031)	(0.031)	(0.035)	(0.035)	(0.034)	(0.034)
Religion: Evangelical Pentecostal or other evangelical		-0.177***	-0.172***	-0.176***	-0.167***	-0.173***	-0.172***	-0.160***	-0.151***	-0.153***	-0.155***
		(0.044)	(0.044)	(0.044)	(0.043)	(0.045)	(0.045)	(0.053)	(0.052)	(0.052)	(0.052)
Religion: Evangelical Traditional		-0.097**	-0.093**	-0.096**	-0.090**	-0.089**	-0.089**	-0.088**	-0.082*	-0.082*	-0.082*
		(0.038)	(0.038)	(0.038)	(0.037)	(0.039)	(0.038)	(0.045)	(0.044)	(0.044)	(0.044)

Religion: Others/No Relig.	-0.063**	-0.058*	-0.056*	-0.053*	-0.055*	-0.056*	-0.063*	-0.059	-0.059*	-0.059*
	(0.032)	(0.032)	(0.032)	(0.031)	(0.032)	(0.032)	(0.037)	(0.037)	(0.036)	(0.036)
Income: 1 - 2 minimum wages		0.058***	0.058***	0.055**	0.057**	0.056**	0.072**	0.068**	0.060**	0.061**
		(0.022)	(0.022)	(0.022)	(0.023)	(0.023)	(0.028)	(0.028)	(0.028)	(0.028)
Income: 10 minimum wages or more		0.067**	0.068**	0.067**	0.077**	0.077**	0.099***	0.091***	0.080**	0.082**
		(0.029)	(0.029)	(0.029)	(0.030)	(0.030)	(0.033)	(0.033)	(0.034)	(0.034)
Income: 2 - 3 minimum wages		0.090***	0.092***	0.088***	0.098***	0.097***	0.096***	0.092***	0.084***	0.084***
		(0.026)	(0.026)	(0.026)	(0.026)	(0.026)	(0.031)	(0.032)	(0.032)	(0.032)
Income: 3 - 5 minimum wages		0.078***	0.076***	0.072***	0.076***	0.077***	0.090***	0.084***	0.069**	0.069**
		(0.027)	(0.026)	(0.026)	(0.027)	(0.028)	(0.032)	(0.032)	(0.032)	(0.032)
Income: 5 - 10 minimum wages		0.084**	0.083**	0.077**	0.086**	0.086**	0.089**	0.083**	0.068	0.070
		(0.037)	(0.037)	(0.037)	(0.038)	(0.038)	(0.042)	(0.042)	(0.043)	(0.043)
Income: Do not know/ Prefer to not answer		-0.006	-0.003	-0.006	-0.006	-0.004	0.009	0.009	0.008	0.008
		(0.024)	(0.024)	(0.024)	(0.025)	(0.025)	(0.032)	(0.032)	(0.032)	(0.032)
Insignificant variables										
Subjective knowledge			0.018	0.018	0.014	0.013	0.016	0.015	0.013	0.013
			(0.012)	(0.012)	(0.013)	(0.013)	(0.015)	(0.015)	(0.015)	(0.015)
Trust in scientists				0.063**	0.058**	0.059**	0.039	0.039	0.040	0.040
				(0.026)	(0.028)	(0.028)	(0.035)	(0.035)	(0.035)	(0.035)
Egalitarianism worldview					0.013	0.013	0.007	0.008	0.005	0.005
					(0.013)	(0.013)	(0.015)	(0.015)	(0.015)	(0.016)
Personal experience (extreme weather events)						0.034	0.045*	0.046*	0.046*	0.046*
						(0.022)	(0.028)	(0.027)	(0.027)	(0.027)
Political ideology: Progressive							0.010	0.009	0.008	0.008
							(0.019)	(0.019)	(0.019)	(0.019)
Female								-0.030	-0.032	-0.031
								(0.020)	(0.020)	(0.020)
Age (Years)									0.001*	0.001*

										(0.0007)	(0.0007)
Race: Black											0.047
											(0.043)
Const	0.234***	0.318***	0.275***	0.222***	0.193**	0.184**	0.160*	0.185*	0.205*	0.182*	0.173
	(0.065)	(0.072)	(0.074)	(0.081)	(0.081)	(0.088)	(0.089)	(0.107)	(0.108)	(0.109)	(0.110)
Fit statistics											
Observations	4,270	4,270	4,270	4,252	4,252	3,987	3,987	2,887	2,887	2,887	2,887
R^2	0.12279	0.12743	0.13355	0.13508	0.13676	0.13932	0.13996	0.14494	0.14584	0.14735	0.14774

Notes: Results from stepwise ordinary least squares (linear probability) models regressing the perception of climate change consequences on a set of independent variables and socio-demographic characteristics. We gradually include the insignificant variables (at the 95% level) from our main specification from columns 5 - 9. Coefficients multiplied by one hundred are percentage point changes in the probability of believing climate change impacts will be negative given a unit increase in the covariates. Heteroskedasticity-robust standard errors in parentheses. Reference baseline for Education is "Elementary (Primary) or less", Religion is "Atheist" and Income is "0 – 1 minimum wages". Signif. Codes: ***: 0.01, **: 0.05, *: 0.1. p-values from standard two-sided t-tests for the null hypothesis of a zero average parameter using the reported standard errors in parentheses.
