# Supplementary material for: Climate change beliefs and their correlates in Latin America
Source: Nat Commun. 2023 Nov 9;14:7241. doi: 10.1038/s41467-023-42729-x (PMC10636181; doi:10.1038/s41467-023-42729-x)
Supplement: Supplementary file 2 — Reporting Summary [file 41467_2023_42729_MOESM2_ESM.pdf]

nature portfolio

Corresponding author(s): DBPR NCOMMS-22-41583C

Last updated by author(s): Oct 8, 2023

Reporting Summary

Nature Portfolio wishes to improve the reproducibility of the work that we publish. This form provides structure for consistency and transparency in reporting. For further information on Nature Portfolio policies, see our Editorial Policies and the Editorial Policy Checklist.

Statistics

For all statistical analyses, confirm that the following items are present in the figure legend, table legend, main text, or Methods section.

n/a	Confirmed	
☐	☒	The exact sample size (*n*) for each experimental group/condition, given as a discrete number and unit of measurement
☐	☒	A statement on whether measurements were taken from distinct samples or whether the same sample was measured repeatedly
☐	☒	The statistical test(s) used AND whether they are one- or two-sided<br>*Only common tests should be described solely by name; describe more complex techniques in the Methods section.*
☐	☒	A description of all covariates tested
☐	☒	A description of any assumptions or corrections, such as tests of normality and adjustment for multiple comparisons
☐	☒	A full description of the statistical parameters including central tendency (e.g. means) or other basic estimates (e.g. regression coefficient) AND variation (e.g. standard deviation) or associated estimates of uncertainty (e.g. confidence intervals)
☐	☒	For null hypothesis testing, the test statistic (e.g. *F*, *t*, *r*) with confidence intervals, effect sizes, degrees of freedom and *P* value noted<br>*Give P values as exact values whenever suitable.*
☒	☐	For Bayesian analysis, information on the choice of priors and Markov chain Monte Carlo settings
☒	☐	For hierarchical and complex designs, identification of the appropriate level for tests and full reporting of outcomes
☒	☐	Estimates of effect sizes (e.g. Cohen's *d*, Pearson's *r*), indicating how they were calculated

Our web collection on statistics for biologists contains articles on many of the points above.

Software and code

Policy information about availability of computer code

Data collection	No software was used for data collection. The process was conducted by Netquest, an international polling company. Netquest is certified with ISO 26362, an international high-quality standard for online panels, and complies with the European Society for Opinion and Market Research (ESOMAR).
Data analysis	All statistical analysis were conducted using the R software (version 4.2.2) The integrated development environment (IDE) of choice was RStudio.

For manuscripts utilizing custom algorithms or software that are central to the research but not yet described in published literature, software must be made available to editors and reviewers. We strongly encourage code deposition in a community repository (e.g. GitHub). See the Nature Portfolio guidelines for submitting code & software for further information.

Data

Policy information about availability of data

All manuscripts must include a data availability statement. This statement should provide the following information, where applicable:

- Accession codes, unique identifiers, or web links for publicly available datasets
- A description of any restrictions on data availability
- For clinical datasets or third party data, please ensure that the statement adheres to our policy

The data collected and used in this study, as well as all coding scripts, are available at Harvard Dataverse as Spektor, Matias; Fasolin, Guilherme; Camargo, Juliana, 2022, “Replication Data for: Climate Change Beliefs and their Correlates in Latin America”, https://doi.org/10.7910/DVN/F4KNNS, Harvard Dataverse, V1.

Human research participants

Policy information about studies involving human research participants and Sex and Gender in Research.

Reporting on sex and gender	Sex, as a biological attribute, was considered in our study design. The information was self.-reported by survey participants when answering the following question: "What is your sex?".
Population characteristics	See below.
Recruitment	Respondents were recruited by Netquest through an opt-in recruitment method, where respondents are randomly selected for survey invitation, using population quotas.
Ethics oversight	This study was approved by the Ethical Review Committee of the Fundação Getulio Vargas (FGV) (Ethics no. 053/2021). All participants informed voluntary consent with an IRB-approved consent protocol before being allowed to proceed to the full questionnaire. The survey did not collect identifying information about respondents and/or use any type of deception

Note that full information on the approval of the study protocol must also be provided in the manuscript.

Field-specific reporting

Please select the one below that is the best fit for your research. If you are not sure, read the appropriate sections before making your selection.

☐ Life sciences ☒ Behavioural & social sciences ☐ Ecological, evolutionary & environmental sciences

For a reference copy of the document with all sections, see nature.com/documents/nr-reporting-summary-flat.pdf

Behavioural & social sciences study design

All studies must disclose on these points even when the disclosure is negative.

Study description	Quantitative cross-sectional survey.
Research sample	We targeted respondents from seven countries in Latin America (Argentina, Brazil, Chile, Colombia, Ecuador, Peru, and Mexico), which represent over 80% of greenhouse gas emissions (GHG) in the region. Respondents were recruited to match the demographic composition, particularly of gender, age, and education, laid out by the national census of each country surveyed.<br><br>The final sample comprises 5.400 participants, all of them 18 years of age or over. The sample for each country is balanced with respect to sex, closely approximating official statistics.
Sampling strategy	Netquest, the polling company, provides comprehensive national panels in Latin America which allow us to approximate the national representativeness of the participating countries. Netquest builds its online panels through an opt-in recruitment method, where respondents are randomly selected for survey invitation, using population quotas to produce nationally diverse samples.
Data collection	Participants anonymously answered the survey through an online platform on their personal devices (i.e., computer or cellphones). No researcher was present during the surveys.
Timing	This study uses survey data collected between October 2021 and November 2021.
Data exclusions	No data was excluded from the analysis.
Non-participation	No participants dropped out. Each country surveyed featured roughly 830 respondents. The only exception was Ecuador, where just 421 respondents were interviewed due to panel coverage constraints.

Randomization	The study did not require treatment randomization.

Reporting for specific materials, systems and methods

We require information from authors about some types of materials, experimental systems and methods used in many studies. Here, indicate whether each material, system or method listed is relevant to your study. If you are not sure if a list item applies to your research, read the appropriate section before selecting a response.

Materials & experimental systems

n/a	Involved in the study
☒	☐ Antibodies
☒	☐ Eukaryotic cell lines
☒	☐ Palaeontology and archaeology
☒	☐ Animals and other organisms
☒	☐ Clinical data
☒	☐ Dual use research of concern

Methods

n/a	Involved in the study
☒	☐ ChIP-seq
☒	☐ Flow cytometry
☒	☐ MRI-based neuroimaging
